# Activating Interstitial Oxygen Conduction in Sr_3_V_2_O_8_ Palmierite Oxides Through Minimal Mo-Substitution

**DOI:** 10.1021/acs.chemmater.6c01807

**Published:** 2026-10-13

**Authors:** Abdulkadir Olatunbosun Biffo, Anastasia K. Lavrinenko, Hailin Ma, Shengnan Zhang, Till Frümling, Alexandros Vasileiadis, Theodosios Famprikis, Pedro B. Groszewicz

**Affiliations:** † Department of Radiation Science and Technology, Delft University of Technology, Delft 2629JB, Netherlands; ‡ Technical University of Darmstadt, Department of Materials and Earth Science, Darmstadt 64287, Germany; § Inorganic Chemistry Laboratory, University of Oxford, Oxford OX1 3QR, United Kingdom; ∥ 28340 Helmholtz Zentrum Berlin für Materialien und Energie, Berlin 14109, Germany

## Abstract

The palmierite oxides are a promising family of ion conductors, and understanding how compositional modification affects their structure and, in turn, their ionic conductivity is pivotal for unlocking high ionic conductivity in these solid electrolytes. Here, we investigate the Sr_3_V_2‑x_Mo_*x*_O_8+x/2_ series (0 ≤ *x* ≤ 0.4), establishing a solubility limit at x = 0.2, and demonstrating that Mo-substituted compositions exhibit bulk O^2–^ conductivity approximately 2 orders of magnitude higher than the pristine composition at 600 °C. Combined X-ray and neutron diffraction refinement shows little change in the average crystallographic structure, prompting questions about its local structure and dynamics. Using *ab initio* molecular dynamics, bond-valence site-energy calculations, and ^51^V and ^17^O solid-state nuclear magnetic resonance spectroscopy supported by density functional theory calculations, we demonstrate that Mo^6+^ incorporation generates a new oxygen environment consistent with the formation of a charge-compensating interstitial O3 site. While V^5+^ remains tetrahedrally coordinated throughout the series, Mo^6+^ exhibits both MoO_4_ and distorted trigonal bipyramidal MoO_5_ coordination polyhedra, with the latter arising from the occupation of O3. This O3 site is proposed to bridge neighboring MO_n_ polyhedra along the ab-plane, shortening the O^2–^ jump distances and shifting the conduction mechanism from a 3D cooperative rotational mechanism to a 2D interstitial-based one.

## Graphical abstract

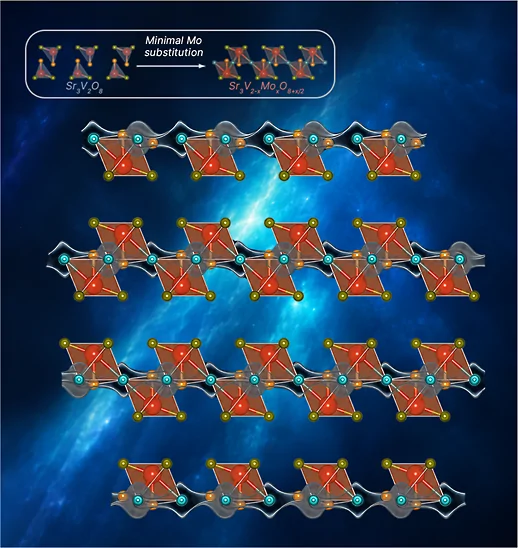

## Introduction

Ionic conductors play a crucial role in hydrogen-based technologies, such as electrolyte materials for solid oxide fuel cells (SOFCs) and solid oxide electrolyzers (SOECs).^1–7^ Currently available electrolyte materials, such as yttria-stabilized zirconia, require operating temperatures above 700 °C, leading to problems including component degradation, longer start-up times, and high material costs.^2,8^ These drawbacks hinder the widespread adoption and commercialization of SOFCs and SOECs, and addressing these challenges requires developing ion conductors for intermediate temperatures (300–600 °C).

Several examples of materials with high ionic conductivity at the intermediate temperature range have been reported, such as silicon and germanium apatites (La_9.33_Si_6‑x_Ge_*x*_O_26_),^9^ LaSrGa_3_O_7_ materials,^10^ La_2_Mo_2_O_9_,^11,12^ Bi_4_V_2_O_11_ compounds,^13^ and perovskites.^14–18^ Understanding how structural features, particularly oxygen defects, govern the dynamics of ion conduction in these oxides is essential for establishing the structure–property correlation to guide the design of better ionic conductors.

Among these, hexagonal perovskite derivatives are a group of materials that have demonstrated significant proton and O^2–^ ion conductivity in the intermediate temperature range, which is substantially higher than that of the most commercially available yttria-stabilized zirconia.^14,16,19^ Among these derivatives, Ba_3_NbMoO_8.5_ ^19^ and Ba_7_Nb_4_MoO_20_,^14^ which contain stacked perovskite- and palmierite-like layers along the *c*-axis of their respective crystal structures, exhibit the highest conductivities.

Given the critical role of palmierite-like layers in these high-performing conductors, the ionic conduction of palmierite oxides with the composition A_3_V_2_O_8_ (A = Sr, Ba) (Figure 1) has been explored.^20^ However, these compositions exhibit substantially lower O^2–^ ion conductivity compared to Ba_3_NbMoO_8.5_ ^19^ and Ba_7_Nb_4_MoO_20_ ^14^ hexagonal perovskite derivatives: with Sr_3_V_2_O_8_ showing higher bulk O^2–^ ion conductivity compared to Ba_3_V_2_O_8_ in the range of (μS cm^–1^)^20^ at 600 °C compared to the mS cm^–1^ range of the hexagonal perovskite derivatives.^14,19^ This lower conductivity can be linked to the mechanism of O^2–^ ion conduction in these palmierite oxides, which has been reported to proceed via the so-called vacancy-driven three-dimensional migration process, where cooperative rotation of both the oxygen-deficient VO_3_ unit and VO_4_ unit allows the migration of an O^2–^ ion via the formation of an intermediate V_2_O_7_ dimer that splits in a repeated manner.^20,21^

**Figure 1:**
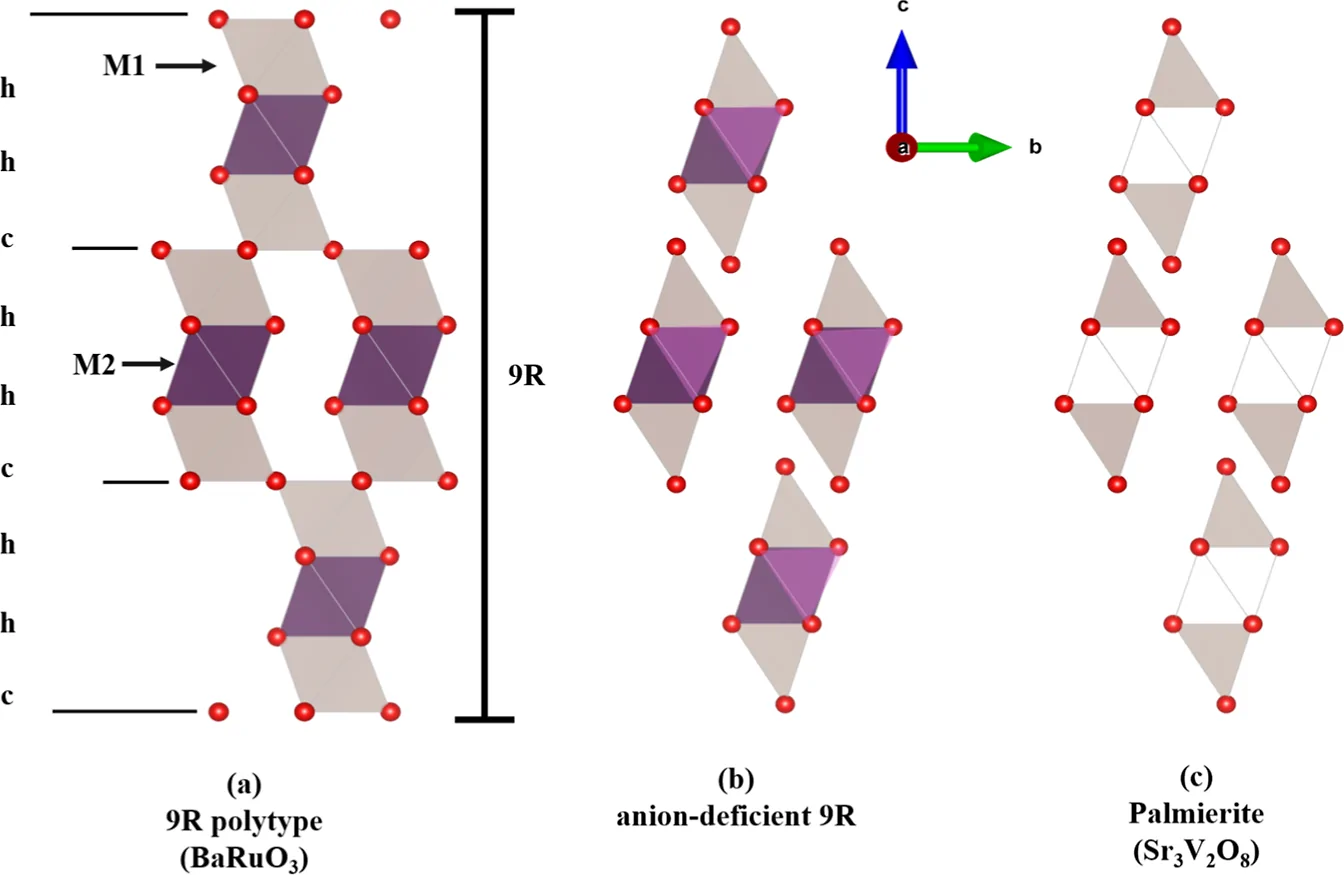
Projection along [010] of the structure of 9R BaRuO_3_ depicts the relationship between (a) 9R hexagonal perovskite polytype and palmierite-type derivative structures; (b) anion deficiency and partially occupied face-sharing octahedra; (c) palmierite structure with empty octahedra. Colored polyhedra denote the different crystallographically equivalent metal-cation sites. The Sr/Ba cationic sites are not included for illustrative purposes.

Recent work suggests that modifying the palmierite structure can significantly enhance conductivity. A-site substitution of La^3+^ for Sr^2+^ in Sr_3‑3x_La_2*x*_V_2_O_8_ (where x = 0.15) has been reported to improve the bulk O^2–^ ion conductivity of palmierite structure by almost an order of magnitude at 700 °C compared to Sr_3_V_2_O_8_.^22^ The incorporation of La into the structure was found to order the cationic vacancies and increase the distortion of the VO_4_ tetrahedra, thereby facilitating the formation of a continuous diffusion network and enhancing the cooperative VO_4_ rotational dynamics, which in turn influence ion transport.^22^ Besides that, the introduction of oxygen interstitials into the palmierite structure via a design principle in which Mo^6+^ and Ti^4+^ replace all V^5+^ cations (Ba_3_Ti_0.9_Mo_1.1_O_8.1_) was demonstrated to significantly improve bulk O^2–^ ion conduction and to alter the conduction mechanism from a 3D cooperative rotational pathway to a 2D interstitial-driven pathway.^23^

Whilst significant, these findings raise a fundamental question regarding the structural requirements for activating this conduction mechanism. Specifically, it remains unclear whether interstitial oxygen transport requires an extended network of transition-metal cations capable of adopting coordination numbers greater than four, or if a small concentration of a highly flexible aliovalent substituent (e.g., Mo^6+^) is sufficient to trigger the same behavior.

Addressing this question is highly pertinent given the narrow compositional range over which the palmierite structure accommodates aliovalent cations, in particular Mo^6+^ substitution.^24^

Here, we report the structural and O^2–^ ionic transport properties of the Sr_3_V_2‑x_Mo_*x*_O_8+x/2_ series (0 ≤ *x* ≤ 0.4). The incorporation of Mo^6+^ into the palmierite structure enhances the bulk O^2–^ conductivity by more than 2 orders of magnitude compared to the pristine Sr_3_V_2_O_8_ composition. A combination of ^17^O ss-NMR and first-principles calculations of NMR parameters resolves new oxygen environments introduced by Mo incorporation, and AIMD simulations identify a crystallographic site (O3) populated by the charge-compensating oxygen. The simulations further reveal that V^5+^ retains tetrahedral coordination geometry throughout the series, whereas Mo^6+^ maintains flexible polyhedral coordination (MoO_4_ and MoO_5_), which dominates oxygen transport via a two-dimensional interstitial-mediated diffusion pathway in the ab-plane and is distinct from the reported VO_4_-rotation-driven mechanism reported for the pristine Sr_3_V_2_O_8_ composition.^20^

## Experimental Section

### Synthesis

The Sr_3_V_2‑x_Mo_*x*_O_8+x/2_ series (0 ≤ *x* ≤ 0.4) was synthesized using the solid-state approach. Stoichiometric mixtures of SrCO_3_ (Merck Sigma, 99.9+ % metals basis), V_2_O_5_ (Merck Sigma, 99.9% metals basis), and MoO_3_ (Merck Sigma, 99% metals basis) were ground using a mortar and pestle until the mixture homogenized. The ground mixture was pressed into a pellet and calcined at 1100 °C for 10 h. The x = 0 composition was phase-pure after this initial step; however, the step was repeated once for the 0.1 and 0.2 samples to further improve phase purity. In contrast, the samples with x = 0.3 and 0.4 showed no improvement after more than one recalcination step. The ^17^O isotopic enrichment of the Sr_3_V_2‑x_Mo_*x*_O_8+x/2_ series (0 ≤ *x* ≤ 0.2) for NMR spectroscopy was performed using a custom-built experimental setup as documented in the SI. The diffraction patterns recorded before and after ^17^O enrichment of the relevant compositions are depicted in Figure S3c–d.

### X-ray and Variable Temperature Neutron Diffraction

Laboratory X-ray powder diffraction (XRD) patterns were collected using a PANalytical X’Pert Pro X-ray diffractometer equipped with a Cu Kα source (1.54 Å) operated at 45 kV, 40 mA. The XRD patterns were recorded over the range of 10° < 2θ < 100°. A LaB_6_ NIST standard (NIST660C) was used for the diffractometer’s calibration and performance qualification.

Neutron powder diffraction (NPD) experiments were performed using the PEARL diffractometer at the TU Delft Reactor Institute (the Netherlands).^25^ Approximately 4.0 g of each sample was loaded into a 6 mm-diameter can made of a V–Ni null-scattering alloy. The sample can was then placed in a neutron-transparent vacuum box connected to a primary vacuum (∼10–3 mbar). Diffraction patterns were measured over approximately 18 h using a wavelength of 1.667 Å, which was selected using the (533) reflection of a single-crystal Ge [533] monochromator. For high-temperature measurements, the temperature was controlled via a resistive heater wrapped around the sample can.

### Nuclear Magnetic Resonance (NMR) Spectroscopy

Magic Angle Spinning (MAS) ^51^V NMR spectra were recorded on a Bruker Ascend 500 spectrometer (B_0_ = 11.7 T) with a NEO console at a resonance frequency of 131.557 MHz (^51^V). Samples were spun at the desired frequencies in a 1.9 mm rotor. A Bruker two-channel DVT 1.9 mm MAS probe was used for all measurements. The chemical shifts were referenced with respect to V_2_O_5_ (99.99%, Aldrich), which has an apparent chemical shift of −613.2 ppm. A rotor-synchronized-solid-echo (π/2–τ–π/2) pulse sequence, with a solid π/2 pulse length of 1.5 ms, was used for data acquisition. The acquired ^51^V spectra were simulated using the mrsimulator Python package.^26^

^17^O NMR spectra were recorded on Bruker Ascend 500 MHz (B_0_ = 11.7 T) and 850 MHz (B_0_ = 20 T) with a NEO console operating at 67.8 and 115.26 MHz (^17^O). The chemical shift was referenced with respect to ^17^O-enriched CeO_2_, which has an isotropic ^17^O chemical shift of +877 ppm. The sample was spun at the desired frequency in a 1.9 mm rotor in a Bruker two-channel MAS probe.

### Computational Details

Periodic density functional theory (DFT) calculations were performed in CASTEP^27^ utilizing the PBE functional.^28^ A planewave energy cutoff of 570 eV was used. The first Brillouin zone was sampled using a 3 × 1 × 3 Monkhorst–Pack grid. Rhombohedral 1 × 2 × 1 Sr_3_V_2_O_8_ and Sr_3_VMoO_8.5_ supercells were created and optimized before the calculation of NMR parameters, with all atomic coordinates and cell parameters allowed to vary. Sr_3_V_2‑x_Mo_*x*_O_8+x/2_ with x = 1 was used to probe the effect of Mo-substitution on the local environment of oxygen anions in a less computationally intensive manner. Initial structures were obtained starting from experimentally derived crystallographic positions obtained from the combined refinement of neutron and X-ray diffraction patterns. Because CASTEP requires integer site occupancies, a set of Sr_3_VMoO_8.5_ configurations without partial occupancies was created from the refined average structure. The rhombohedral 1 × 2 × 1 supercell contains two formula units, in which four sites accommodate two V and two Mo, and the excess oxygen occupies one of the partially occupied O3 positions, giving 36 combinations. Enumeration under the symmetry of the parent framework and the supercell translations reduced these to eight inequivalent configurations, which are shown in Figure S1 after geometry optimization. NMR parameters were then computed employing the gauge-including projector augmented wave (GIPAW)^29^ approach to reconstruct the all-electron wavefunction in the presence of a magnetic field. Chemical shielding was calculated using the linear response method and converted to chemical shift values by comparison with reference compounds (Figure S2a and Table S1).^29,30^ The spectral intensities were weighted by the Boltzmann distribution and by the number of each configuration repetition in the full configurational space.^31,32^ The synthesis temperature of 1373 K was used for the Boltzmann probabilities because cation redistribution and configurational equilibrium only occur at elevated temperatures.^32,33^ The averaged spectrum was constructed by representing each calculated shift as a Gaussian of 30 ppm full width at half maximum and summing these over all sites and configurations.

*Ab initio* molecular dynamics (AIMD) simulations based on the Perdew–Burke–Ernzerhof functional for solid-state systems (PBEsol)^28,34^ were performed using the Vienna *Ab initio* Simulation Package (VASP).^35^ Initial structures were built from experimentally-derived crystallographic positions. The ordering of Mo and V cations, along with the occupancies of partially filled oxygen sites in Sr_3_VMoO_8.5_, was assigned randomly. Hexagonal 2 × 2 × 1 supercells were optimized before AIMD and simulated using the canonical (NVT) ensemble with the Nosé–Hoover thermostat.^36,37^ The energy cutoff was reduced to 400 eV, and a gamma-only *k*-point mesh was used. The selected time step was 2 fs for a total computational time of 100 ps.

Bond Valence Site Energy calculations were performed using the *softBV* program and the BVlain Python package, where the latter is modified to fix specific O^2–^ sites as a part of the rigid lattice framework during BVSE calculations.^38,39^ The bond-valence parameters (R_0_ and b) were taken from the standard parameter sets implemented in the *softBV* program. The refinement-derived crystallographic models were used for the calculations, and the migration pathways and barriers for O^2–^ were computed on a dense grid with a resolution of 0.1 Å. Diffusion pathways were visualized in VESTA^40^ by examining the connectivity of the energy isosurface, and by plotting the models for migration barriers from the calculated energy profiles of the pathway segments.

Pellet Preparation for Impedance Measurement.

Pellets of the Sr_3_V_2‑x_Mo_*x*_O_8+x/2_ series (0 ≤ *x* ≤ 0.2) were made by pressing ∼0.32 g of powder using an isostatic press and a die set of 6 mm. The resulting green pellets were sintered at 1200 °C for 6 h. The sintered pellet has a thickness of ∼2.3 mm, a diameter of ∼6 mm, and a relative density of >90% estimated using Archimedes’ method. Symmetrical cells were fabricated by sputtering a Platinum electrode on both sides of the pellets (Pt | ∼90% dense pellet | Pt).

### Electrochemical Impedance Spectroscopy (EIS)

Electrochemical impedance measurements for the fabricated symmetrical Pt | pellet | Pt cells were recorded under O_2_ gas (identical O_2_ atmosphere on both sides of the pellet to avoid chemical-potential gradient imposition across the cell) using an Alpha-A High Performance Frequency Analyzer (Novocontrol Technologies, Germany) in combination with a conventional tube furnace. The environment around the pellets was evacuated at room temperature, pressurized to 200 mbar with O_2_, and the process was repeated at approximately 200 °C during heating. Measurements were performed exclusively in an O_2_-containing atmosphere to suppress any protonic contribution to the measured conductivity (c.f. the proton conduction reported for related palmierite-type compositions under a humidified atmosphere^20,22^ ). Spectra were collected at a signal amplitude of 0.1 V over the frequency range 100 mHz to 3 MHz, from 300 °C to 700 °C in 25 °C temperature steps, at a heating rate of 5 K/min with a 200 s stabilization per step.

The linear Kramers-Kronig (KK) method from Schönleber et al.^41^ was used to perform the KK validation test on measured impedance data using the impedance.py package (Figure S5).^42^ Thus, the KK validation indicates that the collected impedance data are valid and can be modeled for data analysis, which was performed using a combination of RelaxIS software^43^ and the Recursis impedance analysis web app.^44^ The results of the validation test and the fitting process are illustrated in the Electrochemical Impedance spectroscopy section in the SI.

### Inductively Coupled Plasma–Mass Spectroscopy (ICP–MS)

ICP–MS analyses were performed using a Nexio 2000 (Perkin Elmer) Model B, equipped with a Cyclonic spray chamber, a quartz torch, and a quadrupole detector connected to a Dual Rinse Autosampler. An internal standard was used for quantification, and the external calibration was diluted to 0, 10, 20, 50, and 100 ppb.

Approximately 25 mg of each sample was digested in a mixture of 1.5 mL of 65% HNO3 and 4.5 mL of 30% HCl.

With ICP–MS analysis, the elemental composition of all the Sr_3_V_2‑x_Mo_*x*_O_8+x/2_ series (0 ≤ *x* ≤ 0.2) was estimated, and this analysis confirmed a reasonable agreement between the nominal and the measured compositions. Table S2 highlights the results of this analysis.

## Results and Discussion

Figure S3a and b illustrate the evolution of the diffraction patterns and volume of the unit cell of the Sr_3_V_2‑x_Mo_*x*_O_8+x/2_ (0 ≤ *x* ≤ 0.4) series, respectively. With increasing x content, *i.e.*, Mo-substitution, a V_2_O_5_ impurity was observed at x = 0.2 with ∼1.5% weight composition (Figure S3a). This impurity is present at x = 0.3 and 0.4, alongside a SrMoO_4_ impurity with a weight fraction of around 3.5% at x = 0.4.

The volume of the unit cell expands up to x = 0.2, as expected, and this expansion is anisotropic (see Figures S3–S4). However, this trend does not persist for the x = 0.3 and 0.4 compositions, where the parameters remain constant (Figures S3–S4). We thus hypothesize that the solubility limit of the Sr_3_V_2‑x_Mo_*x*_O_8+x/2_ series is approached at x = 0.2. The trend in the lattice parameters from x = 0 to 0.2 is consistent with the Shannon radii of Mo^6+^ (0.410 Å) and V^5+^ (0.355 Å) in a tetrahedral coordination geometry,^45^ as the lattice parameter is expected to increase with increasing x content. We propose that the anisotropic decrease in the c parameter (Figure S4) with increasing x content can be correlated to the higher charge of Mo^6+^ compared to V^5+^, which creates a stronger electrostatic attraction to the surrounding O^2–^ ions and contracts the lattice in the *c* direction.

Figure 2a illustrates the fitted impedance spectra at 600 °C for x = 0.2. The spectra of the Sr_3_V_2‑x_Mo_*x*_O_8+x/2_ compositions were modeled using two R-CPE elements connected in series, representing the deconvolution of bulk and grain-boundary charge-transfer processes (Figure 2a). The electrode contribution, which is a low-frequency tail in the data showing a Warburg-like behavior, was not included in the fit. The bulk and grain-boundary contributions were assigned based on permittivity (see electrochemical impedance spectroscopy section in the SI). This deconvolution process was applied to all spectra of the Sr_3_V_2‑x_Mo_*x*_O_8+x/2_ series (0 ≤ *x* ≤ 0.2) recorded under O_2_ gas over the temperature range of 300 – 700 °C. The evolution of permittivity and the associated RC frequency as a function of inverse temperature is illustrated in Figures S6 and S7. Figure 2b shows the Arrhenius plot of the deconvoluted bulk conductivity of the Sr_3_V_2‑x_Mo_*x*_O_8+x/2_ series (0 ≤ *x* ≤ 0.2) as a function of the inverse temperature, recorded during heating; the corresponding analysis on cooling is shown in Figure S8.

**Figure 2:**
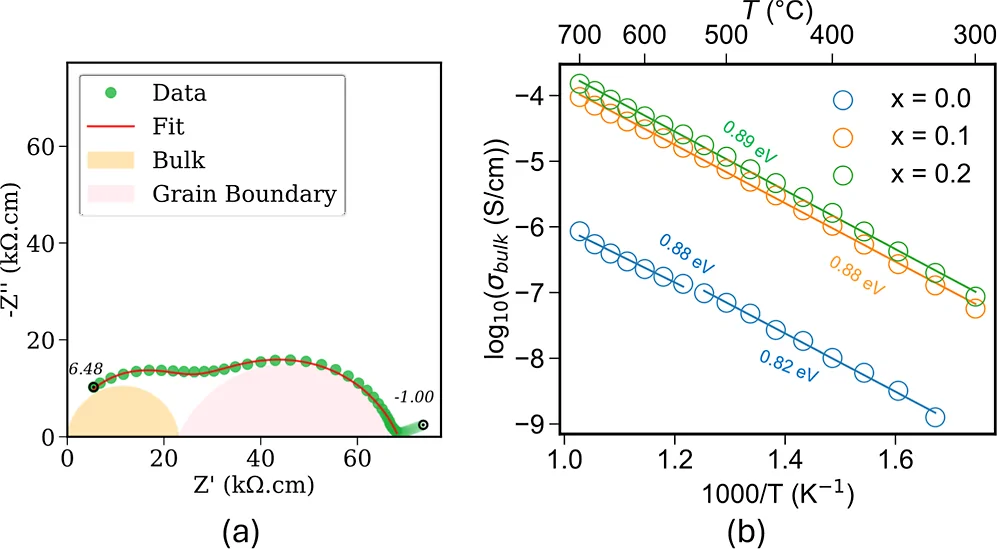
(A) Illustrates the Nyquist plot of the x = 0.2 composition recorded under O_2_ gas at 600 °C. The bulk and grain boundary are deconvoluted with two parallel resistances and constant phase elements (CPEs) (R-CPE) connected in series. (b) Arrhenius plot of the bulk conductivity of the Sr_3_V_2‑x_Mo_*x*_O_8+x/2_ (0 ≤ *x* ≤ 0.2) series (during the heating up under O_2_ gas). The corresponding Arrhenius plots on the cooling are shown in the supplemental file and do not significantly deviate from those for the heating-up processes.

Under O_2_ gas at approximately 600 °C, the bulk conductivity of Sr_3_V_1.8_Mo_0.2_O_8.1_ (x = 0.2) reaches ∼0.05 mS cm^–1^, which is more than 2 orders of magnitude higher than that of Sr_3_V_2_O_8_ (x = 0, ∼0.2 μS cm^–1^), and comparable with Sr_3_V_1.9_Mo_0.1_O_8.05_ (x = 0.1, ∼0.03 mS cm^–1^) (see Figure S8–S9). To identify whether this enhancement originates from a change in the migration barrier or from the pre-exponential term, the bulk conductivity data were fitted to $\sigma =\sigma_{0}\exp\left(\frac{-E_{a}}{k_{B}T}\right)$ ; the resulting activation energies and pre-exponential factors are summarized in Table 1. The activation energies are equal within their fitted uncertainties across the series, with the pristine composition additionally exhibiting a distinct low-temperature regime that is absent once Mo is incorporated (Table 1 and Figure 2b). The pre-exponential factor (σ_0_), by contrast, increases by roughly 2 orders of magnitude over the same interval (Table 1). The macroscopic activation energy of bulk O^2–^ transport is therefore essentially unchanged by Mo-substitution, and the conductivity enhancement is carried almost entirely by σ_0_. The structural origin of this enhancement in conductivity is examined in the analyses that follow.

**Table 1:** Arrhenius Parameters for Bulk O^2–^ Conductivity of the Sr_3_V_2–x_Mo_*x*_O_8+x/2_ Series (0 ≤ *x* ≤ 0.2), Obtained From $\sigma =\sigma_{0}\exp\left(\frac{-E_{a}}{k_{B}T}\right)$ Fits of the Heating Data Measured Under O_2_ ^a^

*composition (x)*	*regime*	*E*_*a*_ *(eV)*	σ_*0*_ *(S cm* ^*–1*^ *)*	σ_0_/σ_0_ *(x = 0)*
0	high-T	0.878 ± 0.019	(3.73 ± 1.22) × 10^–2^	1
0	low-T	0.819 ± 0.054	(1.27 ± 0.88) × 10^–2^	0.3
0.1	single	0.882 ± 0.009	3.83 ± 0.54	103
0.2	single	0.888 ± 0.009	6.61 ± 0.87	177

a The pristine composition (x = 0) shows two Arrhenius regimes; the high-temperature regime is taken as the reference for the σ_0_ ratio. Uncertainties are the standard errors of the linear regression.

To understand the structural change induced by Mo-substitution in the Sr_3_V_2‑x_Mo_*x*_O_8+x/2_ series that underpins the observed enhancement in O^2–^ conductivity, we probed both the average and local structures using diffraction and NMR techniques, respectively.

A combined Rietveld refinement was performed on the room-temperature X-ray and neutron powder diffraction data for pristine Sr_3_V_2_O_8_, using the crystallographic Sr_3_V_2_O_8_ model reported by Fop et al.^20^ as the starting model. Figure 3a depicts the plot of the combined refinement of both X-ray and neutron diffraction patterns of Sr_3_V_2_O_8_ (x = 0). The refinement-derived crystallographic model is illustrated in Figure 3b, with relevant fit parameters and crystallographic information presented in Table S5. This model comprises a framework of isolated VO_4_ units stacked along the *c*-axis, with V surrounded by two types of O^2–^ ions, designated the O1 and O2 sites. The O2 site forms the base of the VO_4_ geometry, while the O1 is the apical site (Figure 3b).^20^

**Figure 3:**
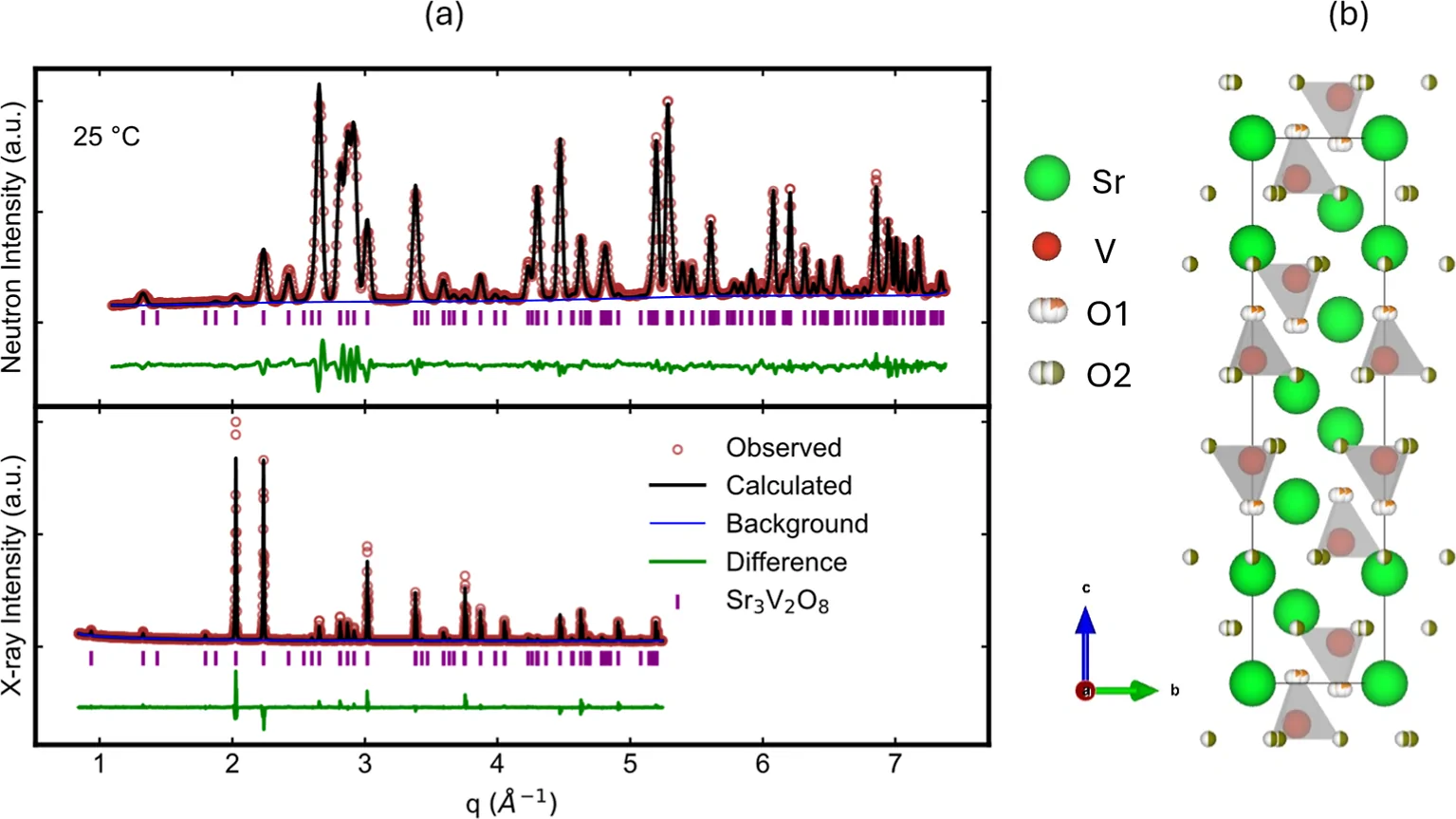
Rietveld (a) fit, and (b) model derived from the combined refinement of room temperature X-ray and neutron diffraction patterns of Sr_3_V_2_O_8_. The O1 site exhibits a 6-fold split, while the O2 shows a 2-fold split due to tetrahedral distortion induced by thermal oscillation of these oxygen sites.

After Le Bail intensity extraction of the powder patterns of Sr_3_V_2_O_8_, Rietveld refinement yielded an overall combined weighted residual (R_wp_) of 7.49% for the X-ray and neutron patterns. High U_iso_ parameters of 0.0137(8) Å^2^ and 0.0190(4) Å^2^ were observed for the O1 and O2 sites, respectively. A difference Fourier map was generated for the neutron patterns (Figure S10), revealing smeared scattering density around the O1 and O2 sites (Figure S10a–d). This observation, coupled with the high U_iso_, motivated the application of 6- and 2-fold splits to O1 and O2, respectively, to better account for O^2–^ ion disorder. These splits, after repeating the refinement, transformed the Wyckoff position of O1 from 6c [0, 0, 0.32242(2)] to 36i [0.0387(2), 0.031(4), 0.32245(1)], and O2 from 18 h [0.49861(2), 0.50139(2), 0.23143(1)] to 36i [0.5276(4), 0.5301(4), 0.2317(8)] with R_wp_ of 6.80%, a significant improvement. The refined U_iso_ value decreased to 0.0032(9) and 0.0062(4) Å^2^ for the average O1 and O2 sites, respectively, followed by a significant reduction in the residual smeared neutron scattering density around these positions (Figure S10a–d). It is worth noting that, to conserve the oxygen stoichiometry, the split atoms were assigned fractional occupancies of 0.167 (O1) and 0.500 (O2), i.e., 6/36 and 18/36, respectively.

This refinement approach was subsequently applied to both room- and high-temperature diffraction patterns recorded across the entire Sr_3_V_2‑x_Mo_*x*_O_8+x/2_ series (0 ≤ *x* ≤ 0.2). Notably, for the x = 0.2 composition, the combined refinement of room-temperature diffraction patterns revealed additional scattering density at Wyckoff position 18 h [0.75, 0.25, 0.24232] (labelled O2_s), approximately 1.06(5) Å from the O2 site. This distance is well below the ionic radii of O^2–^ in a two-fold coordination (1.35 Å),^45^ which rules out simultaneous occupation of both sites and indicates that both O2 and O2_s sites constitute a split-site description of a single O^2–^, distributed between two closely spaced positions. The additional site was tested in the refinement as an independent site. Although stable optimization of the U_iso_, fractional coordinates, and occupancy was obtained for the O2_s site upon its inclusion in the crystallographic model, its inclusion did not significantly improve the χ^2^ or the goodness-of-fit (GOF) parameters compared to the simpler model in which the scattering density is treated as positional disorder of O2 (see Figure S12). In accordance with Occam’s razor, the simpler model without an independent O2_s site is adopted as the refined structure reported. The complete crystallographic information and Rietveld refinements for the Sr_3_V_2‑x_Mo_*x*_O_8+x/2_ series (0 ≤ *x* ≤ 0.2) are presented in Figures S13–S16 and Tables S3–S9. Furthermore, it was observed across the series that the relative occupation of the O^2–^ ion sites remains uniform with both Mo-substitution and increasing temperatures, especially in the x = 0.2 composition, where the observed O2_s site at room temperature is not discernible at 507 °C (Figure S17–18).

An additional neutron diffraction pattern was collected for the x = 0.2 composition at 4 K (Table S10 and Figure S19). At this temperature, the apical O1 site (6c Wyckoff position) refined without the 6-fold split required at room temperature, whereas the basal O2 site (18 h) retained its 2-fold split with a reduced U_iso_ of ∼0.0027(8) Å^2^ (c.f. 0.0111(6) Å^2^), whereas the O2_s site evidenced at RT was no longer discernible. The fact that the 6-fold split is no longer required to model the O1 site at 4 K indicates that the origin of the disorder of the apical-oxygen site is predominantly dynamic. In contrast, the persistence of the 2-fold split at the O2 site indicates that the basal-oxygen displacement is a static positional disorder intrinsic to the structure rather than a thermal effect. The 4 K refinement, therefore, distinguishes a dynamic (apical O1) contribution from a static (basal O2) contribution to the room-temperature oxygen disorder; this distinction is examined further in the sections below.

Given the absence of significant changes in the average structure, the local structures and coordination environments of the involved atoms were examined. Figure 4 shows the ^51^V MAS NMR spectra of Sr_3_V_2‑x_Mo_*x*_O_8+x/2_ series (0 ≤ *x* ≤ 0.2), and Figure S20 illustrates the fitting of the ^51^V MAS NMR spectrum of the series with chemical shift anisotropy and quadrupolar nuclear interaction models.

**Figure 4:**
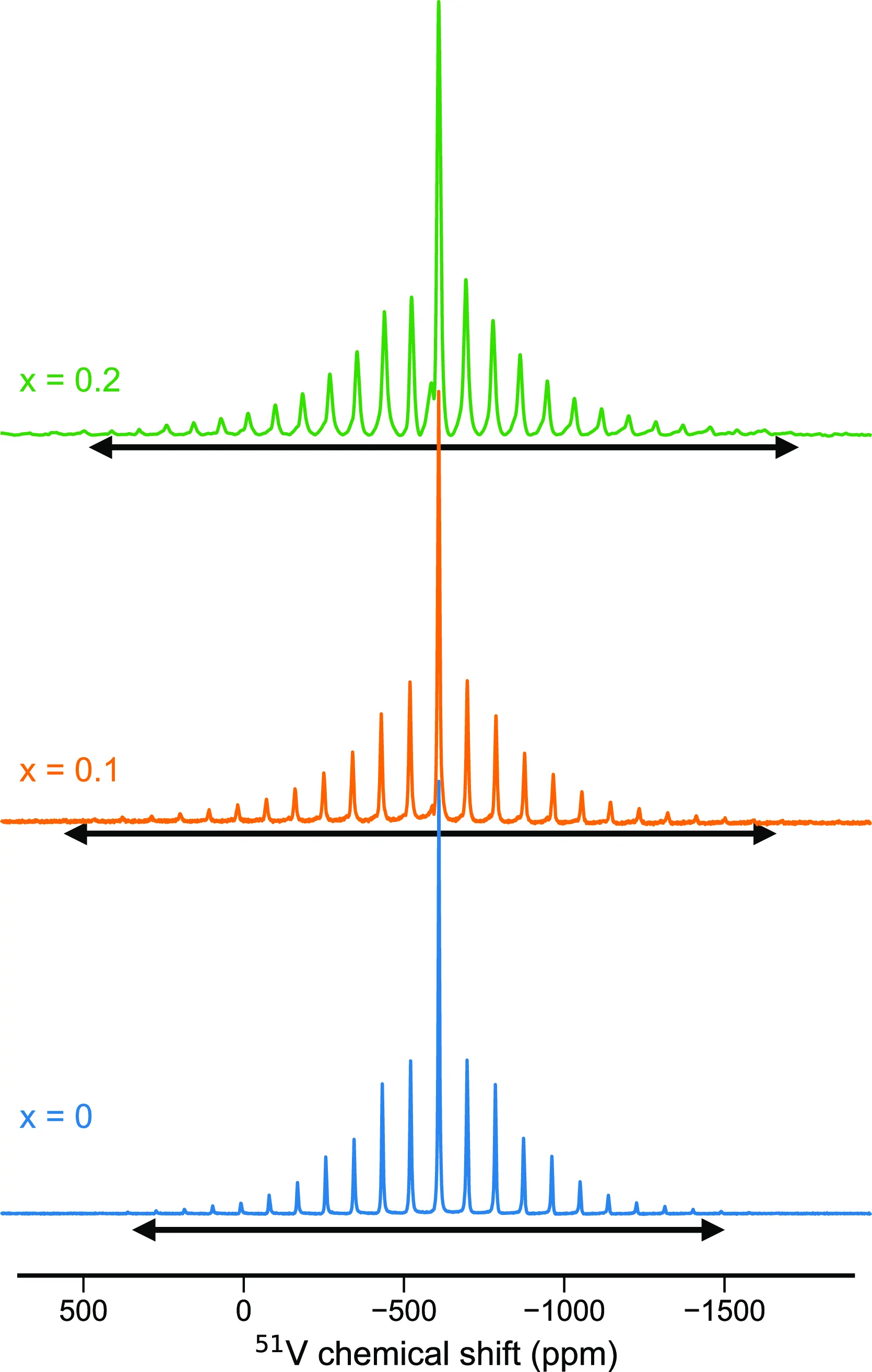
^51^V MAS NMR spectra across the Sr_3_V_2‑x_Mo_*x*_O_8+x/2_ series (0 ≤ *x* ≤ 0.2). The double-headed arrows depict the span of the spinning sidebands as a measure of the magnitude of the probed interaction, e.g., chemical shift or quadrupolar coupling.

For the pristine composition (x = 0), the ^51^V NMR spectrum exhibits a center band at approximately −610 ppm, flanked by spinning sidebands spanning 2500 ppm (Figure 4). In the x = 0.1 and 0.2 compositions, an additional peak is observed at approximately −590 ppm, which increases in relative peak area from 0.76% at x = 0.1 to 5.58% at x = 0.2 (Figures 4 and S21, and Table S12). The peak with apparent chemical shift peak at −610 ppm can be assigned to vanadium in the Sr_3_V_2‑x_Mo_*x*_O_8+x/2_ phase. In comparison, the signal at −590 ppm corresponds to vanadium in the V_2_O_5_ impurity (Figure S3c–d), which manifests from the ^17^O enrichment process (Figure S3c–d). Given the minimal contribution of this impurity, our subsequent analysis focuses on modeling the main Sr_3_V_2‑x_Mo_*x*_O_8+x/2_ phase. The modeling approach used herein is similar to the one reported by Biffo et al.^17^ Modeling of ^51^V spectra across the Sr_3_V_2‑x_Mo_*x*_O_8+x/2_ series provides insight into the local coordination environment of V and how Mo-substitution affects the coordination polyhedra of V. From CSA modeling, the V^5+^ cation is found to occupy a weakly distorted isolated tetrahedral (VO_4_) environment, but no higher coordination numbers, across the Sr_3_V_2‑x_Mo_*x*_O_8+x/2_ series (see the ^51^V section in the SI for more details).

To probe the local O^2–^ environment of the Sr_3_V_2‑x_Mo_*x*_O_8+x/2_ series, we resort to ^17^O NMR spectroscopy. Figure 5 illustrates the ^17^O NMR spectra of the Sr_3_V_2‑x_Mo_*x*_O_8+x/2_ series (0 ≤ *x* ≤ 0.2) recorded at a 20 T magnetic field. The pristine composition (x = 0) exhibits two ^17^O signals at ∼580 and 660 ppm, with relative peak areas of 57% and 43%, respectively, similar to the spectrum recorded at 11.7 T (Figure S22 and Table S13). Upon Mo-substitution (x = 0.1), an additional signal appears at ∼646 ppm, while the initially observed peaks broaden significantly. At x = 0.2, the spectrum displays four ^17^O signals, including a new peak at ∼553 ppm, all of which show noticeable broadening (Figure 5 and Table S14). While further shoulders flanking the main resonances may be surmised for the ^17^O NMR spectra, a finer structure cannot be unequivocally distinguished in light of the limited sensitivity and resolution of the current data sets; the number of fitted lines represents the minimum required to account for the additional spectral features observed upon Mo-substitution. Furthermore, comparison of the 20 T lineshape analyses with the 11.7 T data shows that the line widths scale linearly with B_0_ in Hz (Table S15), indicating that the broadening arises from a distribution of isotropic chemical shifts rather than a quadrupolar effect, in contrast to the pristine composition, whose resonances narrow at higher field. We hypothesize that the broadening of ^17^O resonances upon Mo-substitution could correspond to the formation of new O^2–^ environments arising from differences in Mo—O and V—O bond lengths.

**Figure 5:**
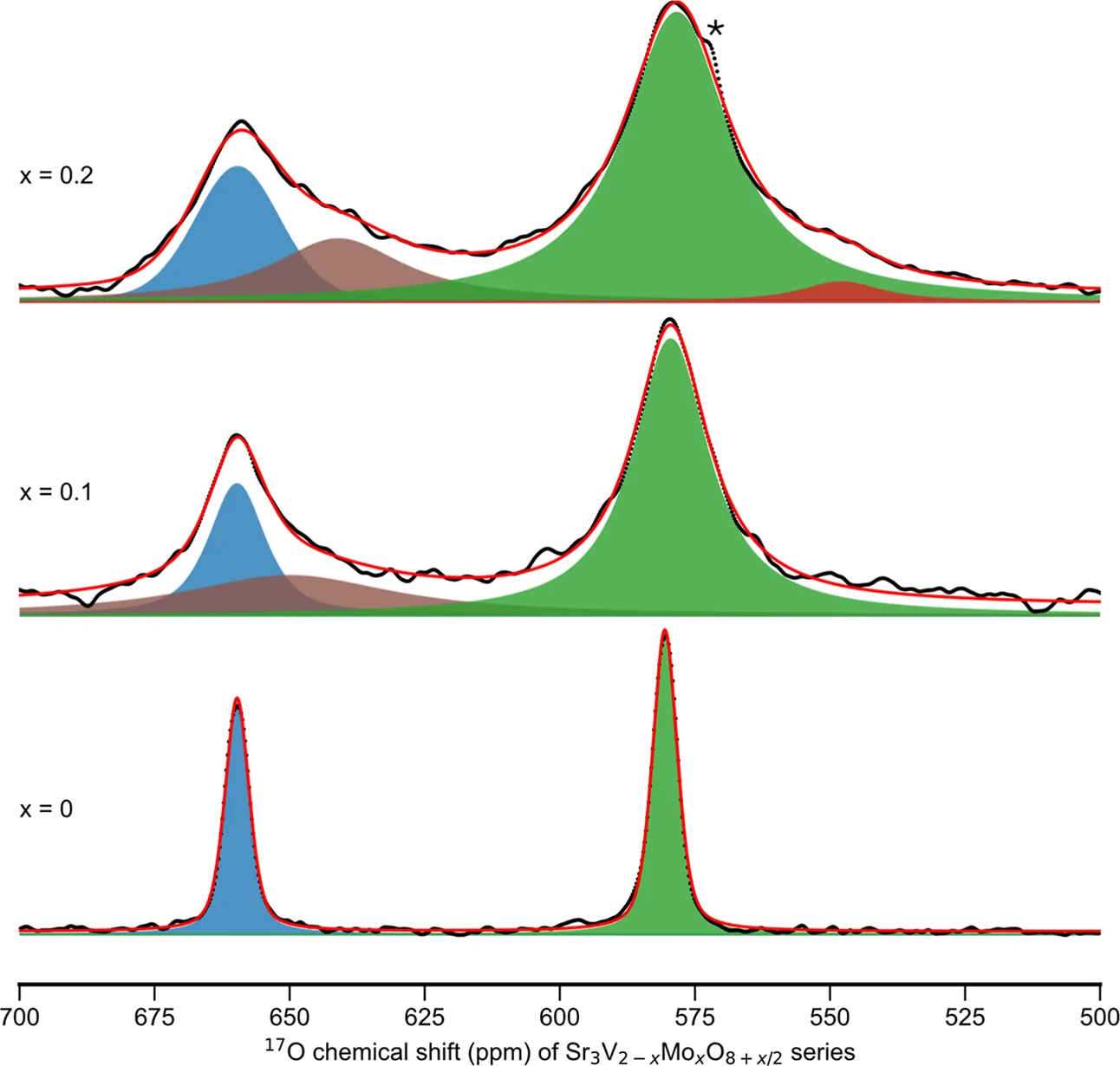
Evolution of ^17^O MAS NMR spectra of the Sr_3_V_2‑x_Mo_*x*_O_8+x/2_ series (0 ≤ *x* ≤ 0.2) with increasing x at 20 T. The plot shows the center band of the respective NMR spectrum, and the peaks were deconvoluted using the IonHop NMR lineshape analysis web-based software.^46^ The asterisk (*) denotes the narrow shoulder at ∼572 ppm in the x = 0.2 spectrum, assigned to the V_2_O_5_/SrMoO_4_ impurity detected after ^17^O enrichment (Figure S3c–d).

To further understand how the ^17^O chemical shift responds to V/Mo cation disorder, we performed first-principles NMR calculations. Using refinement-derived crystallographic models, we generated eight supercells in which the V/Mo ordering was varied while maintaining the Sr_3_VMoO_8.5_ stoichiometry, as described in Computational Details and shown in Figure S1. Sr_3_V_2‑x_Mo_*x*_O_8+x/2_ with x = 1 was used to probe the effect of Mo-substitution on the local environment of oxygen anions in a less computationally intensive manner. Upon Mo incorporation, the additional oxygen introduced to maintain charge balance relaxes into a crystallographic site distinct from the O1 and O2 positions, referred to throughout this manuscript as the O3 position (Figure S1). AIMD simulations at 600 K further confirm this assignment, with peaks in the oxygen probability density distribution attributed to the O3 position at fractional coordinates (0.0, 0.5, 0.0) in the 9e Wyckoff position (Figures 6 and 7).

**Figure 6:**
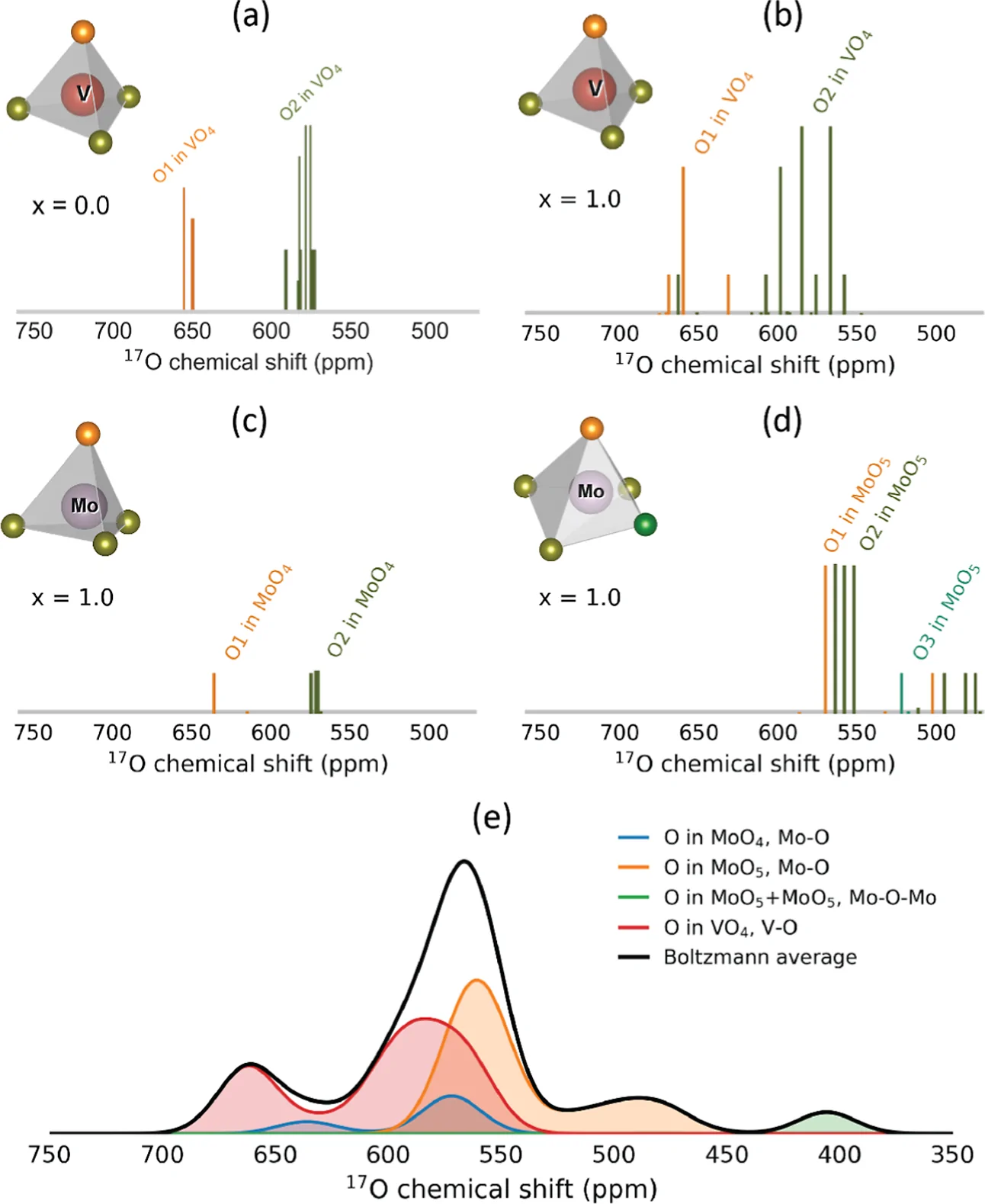
Calculated ^17^O NMR spectra of Sr_3_V_2‑x_Mo_*x*_O_8+x/2_, where peak heights represent the Boltzmann weight of each site. (a) ^17^O NMR peaks of the Sr_3_V_2_O_8_ structure across multiple configurations. (b) ^17^O NMR peaks of the Sr_3_VMoO_8.5_ structure across multiple configurations for VO_4_ tetrahedra. (c) ^17^O NMR peaks of the Sr_3_VMoO_8.5_ structure across multiple configurations for MoO_4_ tetrahedra. (d) ^17^O NMR peaks of the Sr_3_VMoO_8.5_ structure across multiple configurations for MoO_5_ polyhedra. (e) Boltzmann-averaged spectrum of the Sr_3_VMoO_8.5_ structure with each calculated shift represented as a Gaussian of 30 ppm full width at half maximum, decomposed into contributions from each polyhedron type.

**Figure 7:**
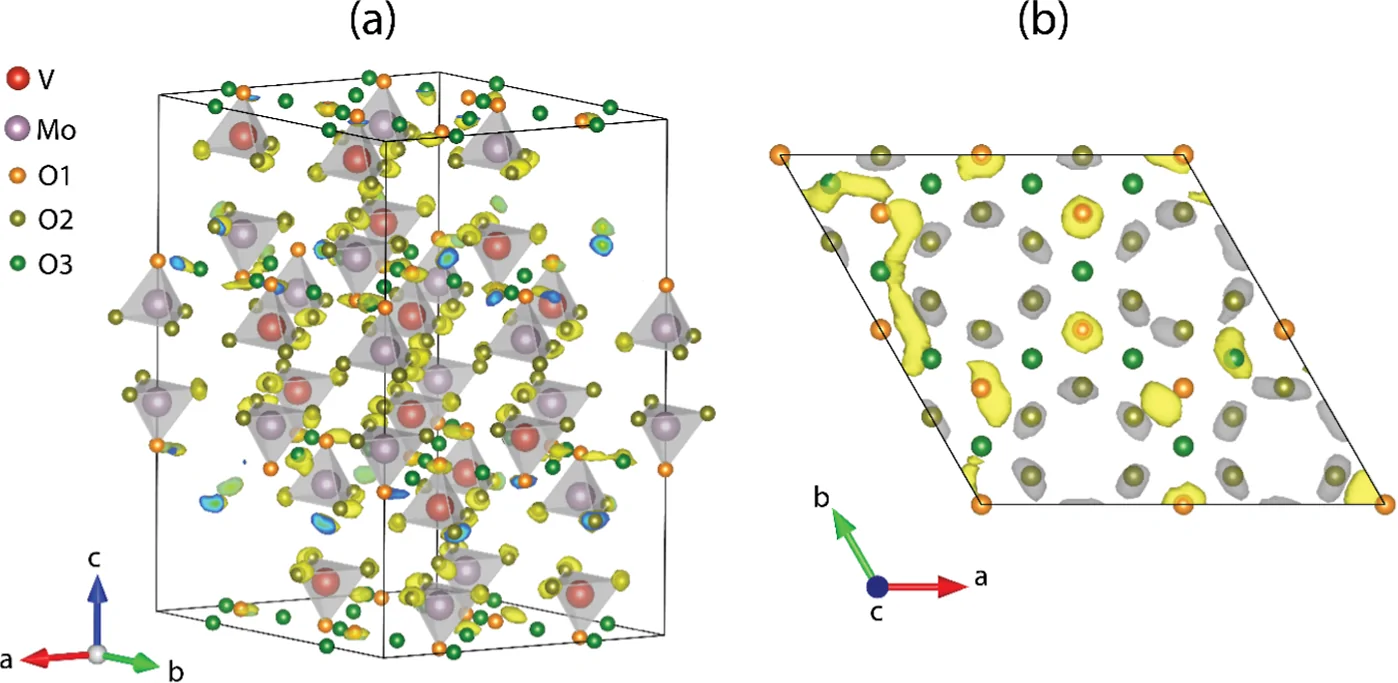
Oxygen mobility in Sr_3_VMoO_8.5_. Crystallographic oxygen sites available for occupation are indicated by orange (O1), olive (O2), and green (O3) spheres. (a) Oxygen probability density distribution from AIMD simulations at 600 K (yellow isosurface) visualized within the full supercell. Sr atoms are omitted for clarity. (b) Oxygen probability density distribution from AIMD simulations at 600 K across three representative oxygen ab-plane: grey isosurface indicates oxygen localized at O2 sites, while yellow isosurface highlights oxygen diffusion pathways involving O1 and O3 sites. Sr, V, and Mo atoms are omitted for clarity.

Based on the calculated ^17^O chemical shift values for the Sr_3_V_2_O_8_ model, which yield a result similar to the experimental spectrum, the two signals in the spectrum for this composition (Figures 5 and 6a) can be assigned to oxygen atoms occupying the apical O1 (∼660 ppm) and basal O2 (∼580 ppm) positions that make up the VO_4_ tetrahedra. Minimal distortion of the VO_4_ units was observed in this composition, consistent with the narrow peak widths in the experimental spectrum (Figure 5). Upon Mo-substitution, the calculated spectra exhibit greater diversity in oxygen environments (Figure 6b), reflecting increased polyhedral disorder. The chemical shifts of the O1 (660 ppm) and O2 (580 ppm) sites observed in the pristine compositions shift to slightly higher values, accompanied by a significant peak broadening, in agreement with the experimental results (Figure 5). The additional peaks observed in the experimental spectra for Mo-substituted compositions can be assigned to oxygen atoms occupying O1 in MoO_4_ (∼647 ppm) and O2 in MoO_4_ (∼548 ppm) (Figure 6c). Peaks corresponding to oxygen atoms at the O1, O2, and O3 positions in MoO_5_ were identified in the calculated spectra within the 500–600 ppm range (Figure 6d), and can also contribute to the experimental peak at ∼548 ppm.

The Boltzmann-averaged spectrum (Figure 6e) illustrates these features and can be contrasted with the experimental one, and it displays a higher contribution from MoO_5_ polyhedra than the experimental spectrum, together with an additional peak at 470–500 ppm. This discrepancy is expected since the calculations were performed at x = 1, whereas the measured compositions are considerably more dilute in Mo. The lowest-energy configurations place the interstitial oxygen bridging two Mo, which converts both cations to MoO_5_ and reduces the MoO_4_ population, accounting for its weak contribution to the averaged spectrum in Figure 6e. Notably, peaks corresponding to O3 oxygen atoms when shared between two MoO_5_ polyhedra appear at a lower chemical shift of ∼400–440 ppm in the calculated spectra, a range where no additional resonances are observed in the experimental spectra. This species requires two neighboring Mo cations, and its probability therefore falls steeply with decreasing Mo content, so its absence from the experimental spectra is consistent with the low Mo stoichiometry. Albeit some discrepancies are expected in peak positions due to the finite set of static 0 K supercell configurations relative to the thermally averaged experimental measurements,^47^ the calculated and experimental average peak positions showed good overall agreement.

Analysis of the V—O and Mo—O radial distribution functions and oxygen coordination numbers (CN) from AIMD simulations at 600 K (Figure S2b) reveals that V retains tetrahedral coordination throughout (CN = 4.0), while Mo exhibits CN values between 4.0 and 5.0, corresponding to a coexistence of MoO_4_ tetrahedra and distorted MoO_5_ polyhedra. This coordination flexibility is consistent with the larger ionic radius (Mo^6+^: 0.41 Å vs V^5+^: 0.355 Å in tetrahedral coordination)^45^ and the more diffuse 4d valence shell of Mo^6+^, both of which are characteristic of second-row d^0^ transition-metal cations and underlie their well-documented variable coordination chemistry in oxides.^48^ A similar effect has been reported in Ba_3_Ti_0.9_Mo_1.1_O_8.1_,^22^ where O3 occupation transforms the M1 cationic site from isolated tetrahedral polyhedra to a mix of four-, five-, and six-fold coordination environments. The associated bond-length analysis shows that V—O distances are shorter than Mo—O at equivalent sites. The analysis of average metal–O bond lengths further reveals that O1 forms shorter bonds with both cations than O2 or O3. These trends correlate directly with the ^17^O chemical shifts (Figure S2c), in which oxygen atoms bonded to V appear at a higher chemical shift than those bonded to Mo, and apical O1 oxygens appear at a higher chemical shift than basal O2 or interstitial O3. The MoO_5_ polyhedra display the broadest metal–O bond-length distributions, corresponding to a larger spread of ^17^O chemical shifts (Figure S2c) and greater polyhedral distortions than the MO_4_ or VO_4_ units. Across the configurations examined, these MoO_5_ polyhedra occur as both isolated units and corner-sharing polyhedra (Figure S1).

Beyond the coordination analysis, we also performed AIMD simulations to elucidate the role of each site in oxygen diffusion. O^2–^ occupying the O3 site acts as a bridging species between the O1 sites of neighboring polyhedra and facilitates long-range transport, while the oxygen density at O2 remains localized with limited contribution to diffusion (Figure 7). This observation is consistent with variable-temperature diffraction, in which the apical O1 is dynamically disordered, whereas the statically disordered basal O2 remains localized. Oxygen transport in the AIMD models is dominated by the Mo-centered polyhedra, whose variable coordination provides the local structural rearrangements required for oxygen hopping. In contrast, the VO_4_ units, constrained to tetrahedral coordination, contribute less to ionic transport. This preferential involvement of Mo-centered polyhedra explains the experimentally observed enhancement in bulk O^2–^ conductivity, as Mo-substitution introduces oxygen-occupational disorder in the palmierite layers and opens two-dimensional diffusion pathways in the ab-plane (Figure 7b).

As a complementary probe of the migration landscape, BVSE calculations were performed on the refined crystallographic model for the Sr_3_V_2‑x_Mo_*x*_O_8+x/2_ series, where in the pristine composition (x = 0) all the O^2–^ sites were mobile, whereas for the x = 0.2 composition, the O2 site was assigned to the rigid-lattice framework (*n.b.* the static force-field interaction potential of the O2 site was nonetheless included to capture its influence on migrating O^2–^).

The O^2–^ conduction pathway in Sr_3_V_2_O_8_ is 3-dimensional, involving all oxygen sites and exhibiting the 3D cooperative rotational mechanism, where the rate-limiting migration pathway involves the hopping of O^2–^ between a BVSE-generated interstitial site (O_int_) and the O2 site, which is responsible for long-range inter-polyhedral 3D diffusion (see the BVSE section in the SI: Figure S23a,b). For the Sr_3_V_1.8_Mo_0.2_O_8.1_ structure, the O2 site was first allowed to be part of the dynamic framework and later constrained within the rigid lattice, consistent with AIMD simulations at 600 K. The landscape of the unconstrained model captures high-barrier 3D O^2–^ jumps between the O1 and O2 sites, which is similar to the one observed in the 3D cooperative rotational pathway in the pristine composition (Figure S23c,d).

In contrast, Figure 8 illustrates the calculated BVSE O^2–^ conduction landscapes and maps for the constrained model with the Sr_3_V_1.8_Mo_0.2_O_8.1_ composition, where a 2-dimensional conduction pathway is evident along the ab-plane, consistent with AIMD observations.

**Figure 8:**
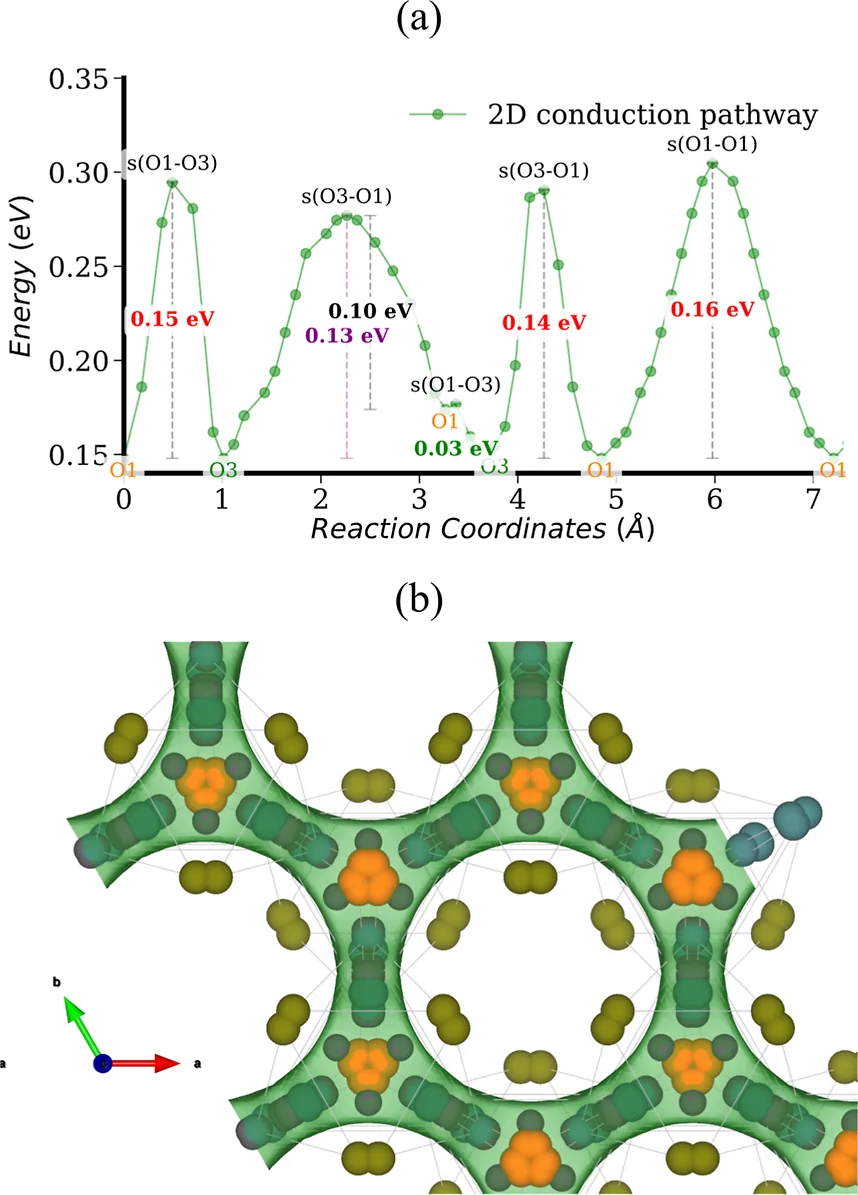
(A) The BVSE landscape of Sr_3_V_1.8_Mo_0.2_O_8.1_ composition, alongside its corresponding map in (b), illustrating the O^2–^ conduction pathways in the composition Sr_3_V_1.8_Mo_0.2_O_8.1_. The Sr and V/Mo atoms are not shown in (b) for visualization purposes.

Three interstitial sites emerge from the calculations: i1 at 36i Wyckoff (0.259, 0.130, 0.657), i2 at 18 h Wyckoff (0.870, 0.435, 0), and i3 at 36i Wyckoff (0.593, 0.806, 0.323). These sites transform the isolated tetrahedral units of the palmierite structure into corner-sharing polyhedral units with mixed coordination, separated by octahedral M2 vacancies (see Figure S24). Furthermore, these interstitial sites are closely related to the AIMD-generated O3 site, differing in their x-, y-, and z-displacements, resulting in a triple multiplicity of the AIMD-derived O3 site, i.e., i1, i2, and i3; nonetheless, for the sake of simplicity, the BVSE-derived interstitial sites are labeled as O3. Once O2 is assigned to the rigid-lattice framework, the lowest-barrier connectivity that remains is a two-dimensional O1–O3 pathway in the ab-plane (Figure 8a,b). This observed 2D conduction pathway in Sr_3_V_1.8_Mo_0.2_O_8.1_ exhibits substantially lower migration barriers than the unconstrained model and the undoped structure (Figure S23), with a maximum barrier of 0.16 eV along the O1–O3 pathway, compared to the 0.66 eV rate-limiting barrier of the 3D cooperative rotational pathway in Sr_3_V_2_O_8_. This near four-fold reduction in the single-hop BVSE barrier highlights the beneficial role of Mo-substitution in enhancing O^2–^ conduction by stabilizing interstitial O3 sites and confining the diffusion pathway to the ab-plane. In addition, the static force-field contribution of the constrained O2 site modifies the local energy landscape, which suppresses the 3D pathway and promotes a 2D interstitial mechanism in which long-range O^2–^ conduction proceeds via cooperative hopping between lattice O1 and interstitial O3 sites in the ab-plane, thereby enhancing bulk O^2–^ conductivity.

We therefore propose that the introduction of the interstitial O3 site via charge compensation, coupled with the connectivity between oxygen sites in the ab-plane, is correlated with the two-orders-of-magnitude enhancement in bulk O^2–^ ion conductivity observed in Mo-substituted compositions at 600 °C. This effect arises from molybdenum’s greater ability to accommodate longer M—O bonds and a higher coordination number than vanadium, as demonstrated by AIMD and DFT calculations, thereby enabling occupation of the under-bonded charge-compensating O3 site. This site contributes to the observed increase in ionic conductivity for O^2–^ not only by increasing the number of charge carriers, but also by driving a mechanistic shift from the 3D cooperative rotational pathway to a 2D interstitial-based mechanism. Given O3’s position between two adjacent MoO_4_ tetrahedra, it facilitates inter-polyhedral O1–O1 ionic jumps by providing an intermediate position, thereby shortening jump distances and reducing the associated single hop energy barriers.

It is worth noting the ionic vs electronic nature of the observed conductivity enhancement, given that V^5+^ and Mo^6+^ are both reducible cations (V^5+^/V^4+^, Mo^6+^/Mo^5+^) capable of supporting electronic-polaron conductivity under reducing atmospheres. The pristine palmierite Sr_3_V_2_O_8_ has been independently characterized as a predominantly ionic conductor under high and intermediate oxygen partial pressure,^20^ indicating that V^5+^ does not contribute appreciable electronic-polaron conductivity in this structure. Moreover, the EIS-derived macroscopic activation barrier is essentially unchanged across the Sr_3_V_2‑x_Mo_*x*_O_8+x/2_ series (0.88–0.89 eV), whereas an electronic-polaron contribution that grew with Mo incorporation would, in general, shift the barrier with increasing Mo, which is not observed. We therefore attribute the enhancement in conductivity to an increase in bulk O^2–^ conductivity. The enhancement is expressed through the pre-exponential factor rather than the macroscopic activation energy: unlike the substitution series^49^ in which σ_0_ and *E*_a_ co-vary through the Meyer–Neldel relation,^50^ aliovalent Mo^6+^ substitution increases the carrier concentration at a fixed macroscopic barrier (Figure S25).

Finally, the DFT-NMR calculations show that, for Sr_3_V_2_O_8_-based structures, ^17^O chemical shifts near 530 ppm arise exclusively from MoO_5_ coordination, which requires occupation of the interstitial O3 site. This provides compelling evidence for O3 occupation and, together with the AIMD and BVSE results, supports the proposed interstitial-based conduction mechanism in the Mo-substituted palmierite series.

## Conclusion

The Sr_3_V_2‑x_Mo_*x*_O_8+x/2_ series (0 ≤ *x* ≤ 0.4) has been synthesized and characterized to test whether the 2D interstitial oxide ion conduction pathway can be activated by an aliovalent substituent with a flexible coordination environment (Mo^6+^) while the majority of transition-metal cations retain exclusive four-fold coordination (V^5+^), or whether it requires an extended network of transition-metal cations able to adopt higher coordination numbers.^23^ X-ray diffraction places the solubility limit at x = 0.2, and impedance spectroscopy under O_2_ gas reveals a bulk conductivity at 600 °C approximately 2 orders of magnitude higher in the Mo-substituted compositions than in the pristine Sr_3_V_2_O_8_. Combined X-ray and neutron diffraction refinement shows only minimal change in the average crystallographic structure across the series, indicating a local origin for this enhancement. AIMD simulations performed at the x = 1 composition identify a new crystallographic site, O3, at the Wyckoff 9e position, populated by charge-compensating interstitial oxygen as a direct consequence of Mo^6+^ incorporation. Long-range O^2–^ transport is mediated by oxygen bonded to Mo and proceeds between O1 and O3 sites in the ab-plane, while the VO_4_ tetrahedra show only limited contribution to the overall O^2–^ dynamics. Consistently, V^5+^ retains a tetrahedral coordination (CN = 4.0) throughout the series, while Mo^6+^ adopts a coexistence of MoO_4_ and distorted trigonal bipyramidal MoO_5_ polyhedra, the latter arising from the occupation of the charge-compensating O3 site. BVSE calculations corroborate this observation and confirm a mechanistic transition from 3D rotation-mediated diffusion to 2D interstitial-based O^2–^ transport along the ab-plane. ^17^O NMR at 20 T, interpreted through DFT-NMR, provides independent evidence consistent with MoO_5_ and therefore with O3 occupation. The conductivity enhancement reflects two coupled contributions arising from the contrasting coordination preferences of V^5+^ and Mo^6+^: an increase in mobile charge-carrier concentration via the interstitial O3 population, and the formation of a connected O1–O3 pathway in the ab-plane. This mechanism is established at low Mo content, with the V^5+^ sublattice retaining fourfold coordination throughout, indicating that coordination flexibility of the substituent cation is sufficient to activate 2D interstitial-based O^2–^ conduction and that an extended network of higher-coordination-number transition-metal cations is not required. These findings establish aliovalent B-site substitution as a mechanistically distinct route to interstitial-based O^2–^ conduction in palmierite-type oxides.

## Supplementary Material



## Notes

The Supporting Information provides further details on the composition, crystal structure, and electrical properties of the Sr_3_V_2‑x_Mo_*x*_O_8+x/2_ series. It includes additional diffraction and refinement results, impedance and conductivity data, and ^51^V and ^17^O NMR spectra, together with supporting calculations and simulations. Structural models, oxide ion migration pathways, and an analysis of Meyer–Neldel compensation behavior are also presented (PDF)

## References

[1] Haile S. M. (2003). Fuel cell
materials and components. Acta Mater..

[2] Steele B. C. H., Heinzel A. (2001). Materials for fuel-cell
technologies. Nature.

[3] Coduri M., Karlsson M., Malavasi L. (2022). Structure–property correlation
in oxide-ion and proton conductors for clean energy applications:
recent experimental and computational advancements. J. Mater. Chem. A.

[4] Shim J. H. (2018). Ceramics
breakthrough. Nat. Energy.

[5] Brisse A., Schefold J., Zahid M. (2008). High temperature water
electrolysis
in solid oxide cells. Int. J. Hydrog. Energy.

[6] Laguna-Bercero M. A. (2012). Recent
advances in high temperature electrolysis using solid oxide fuel cells:
A review. J. Power Sources.

[7] Jacobson A. J. (2010). Materials
for Solid Oxide Fuel Cells. Chem. Mater..

[8] Wachsman E. D., Lee K. T. (2011). Lowering the Temperature of Solid Oxide Fuel Cells. Science.

[9] Slater P. r., Sansom J. e. h., Tolchard J. r. (2004). Development
of apatite-type oxide
ion conductors. Chem. Rec..

[10] Morales M., Roa J. J., Tartaj J., Segarra M. (2016). A review of doped lanthanum
gallates as electrolytes for intermediate temperature solid oxides
fuel cells: From materials processing to electrical and thermo-mechanical
properties. J. Eur. Ceram. Soc..

[11] Georges S., Goutenoire F., Bohnke O., Steil M. C., Skinner S. J., Wiemhoefer H., Lacorre P. (2004). The LAMOX Family of Fast Oxide-Ion
Conductors: Overview and Recent Results. J.New
Mater. Electrochem. Syst..

[12] Olatunbosun Biffo, A. ; Interplay of Oxygen Disorder and Superionic Conductivity in La2Mo2O9. ChemRxiv 2026, 09–16. 10.26434/chemrxiv.15002465/v1

[13] Abraham F., Boivin J. C., Mairesse G., Nowogrocki G. (1990). The bimevox
series: A new family of high performances oxide ion conductors. Solid State Ion.

[14] Fop S. (2020). High oxide ion and proton
conductivity in a disordered hexagonal
perovskite. Nat. Mater..

[15] Fop S., McCombie K., Smith R. I., Mclaughlin A. C. (2020). Enhanced
Oxygen Ion Conductivity and Mechanistic Understanding in Ba3Nb1–xVxMoO8.5. Chem. Mater..

[16] Yashima M., Tsujiguchi T., Sakuda Y., Yasui Y., Zhou Y., Fujii K., Torii S., Kamiyama T., Skinner S. J. (2021). High oxide-ion
conductivity through the interstitial oxygen site in Ba7Nb4MoO20-based
hexagonal perovskite related oxides. Nat. Commun..

[17] Biffo A. O., Famprikis T., Groszewicz B. (2024). P. The role
of vanadium substitution
in the oxygen sublattice disorder of Ba 7 Nb 4 MoO 20 -based hexagonal
perovskite oxide-ion conductors. J. Mater. Chem.
A.

[18] Fop S., McCombie K. S., Wildman E. J., Skakle J. M. S., Mclaughlin C. A. (2019). Hexagonal
perovskite derivatives: a new direction in the design of oxide ion
conducting materials. Chem. Commun..

[19] Fop S. (2016). Oxide Ion Conductivity in the Hexagonal Perovskite Derivative Ba3MoNbO8.5. J. Am. Chem. Soc..

[20] Fop S. (2022). Proton
and Oxide Ion Conductivity in Palmierite Oxides. Chem. Mater..

[21] Kendrick E., Kendrick J., Knight K. S., Islam M. S., Slater P. R. (2007). Cooperative
mechanisms of fast-ion conduction in gallium-based oxides with tetrahedral
moieties. Nat. Mater..

[22] Watson V. (2025). Enhanced Oxide Ion Diffusion by Lanthanum Substitution in the Palmierite
Sr3–3xLa2xV2O8 via Increased Tetrahedral Distortion and Cation
Vacancies. Chem. Mater..

[23] Tawse D. N. (2025). Unlocking the Potential of Palmierite Oxides: High Oxide Ion Conductivity
via Induced Interstitial Defects. J. Am. Chem.
Soc..

[24] Mössner B., Kemmler-Sack S. (1985). 9R-Stapelvarianten
vom Typ Ba3­(B,B′)­2O9–y
mit B,B′ ≡ Mo, W, V, Ti. J. Common
Met..

[25] van
Eijck L. (2016). Design and performance of a novel neutron powder diffractometer:
PEARL at TU Delft. J. Appl. Crystallogr..

[26] Srivastava D. J., Giammar M., Venetos M. C., McCarthy-Carney L., Grandinetti P. J. (2024). MRSimulator: A cross-platform, object-oriented
software
package for rapid solid-state NMR spectral simulation and analysis. J. Chem. Phys..

[27] Clark S. J. (2005). First principles methods
using CASTEP. Z. Für
Krist. - Cryst. Mater..

[28] Perdew J. P., Burke K., Ernzerhof M. (1996). Generalized
Gradient Approximation
Made Simple. Phys. Rev. Lett..

[29] Pickard C. J., Mauri F. (2001). All-electron magnetic
response with pseudopotentials: NMR chemical
shifts. Phys. Rev. B.

[30] Yates J. R., Pickard C. J., Mauri F. (2007). Calculation of NMR chemical shifts
for extended systems using ultrasoft pseudopotentials. Phys. Rev. B.

[31] Grau-Crespo R., Hamad S., Catlow C. R. A., Leeuw N. H. d. (2007). Symmetry-adapted
configurational modelling of fractional site occupancy in solids. J. Phys. Condens. Matter.

[32] Moran R. F. (2019). Ensemble-Based Modeling
of the NMR Spectra of Solid Solutions: Cation
Disorder in Y2­(Sn,Ti)­2O7. J. Am. Chem. Soc..

[33] Grau-Crespo R. (2025). Capturing local compositional fluctuations
in NMR modelling of solid
solutions. Chem. Sci..

[34] Perdew J. P. (2008). Restoring the density-gradient expansion for exchange in solids and
surfaces. Phys. Rev. Lett..

[35] Kresse G., Furthmüller J. (1996). Efficiency of ab-initio total energy calculations for
metals and semiconductors using a plane-wave basis set. Comput. Mater. Sci..

[36] Nosé S. (1984). A unified
formulation of the constant temperature molecular dynamics methods. J. Chem. Phys..

[37] Hoover W. G. (1985). Canonical
dynamics: Equilibrium phase-space distributions. Phys. Rev. Gen. Phys..

[38] Chen H., Wong L. L., Adams S. (2019). SoftBV – a software tool for
screening the materials genome of inorganic fast ion conductors. Acta Crystallogr. Sect. B Struct. Sci. Cryst. Eng. Mater..

[39] Dembart, V. bvlain 0.24.8 documentation. Github Repository. https://bvlain.readthedocs.io/en/latest/index.html(2026-09-16.

[40] Momma K., Izumi F. (2011). VESTA 3 for three-dimensional
visualization of crystal, volumetric
and morphology data. J. Appl. Crystallogr..

[41] Schönleber M., Klotz D., Ivers-Tiffée E. (2014). A Method for
Improving the Robustness
of linear Kramers-Kronig Validity Tests. Electrochim.
Acta.

[42] Murbach M., Gerwe B., Dawson-Elli N., Tsui L. (2020). impedance.py: A Python
package for electrochemical impedance analysis. J. Open Source Softw..

[43] rhd instruments, G. ; Co, K. RelaxIS 3: THE Software for Impedance Spectroscopy Analysis. https://rhd-instruments.de/solutions-and-products/for-eis-data-analysis/relaxis/.(accessed 16 09 2026).

[44] Chukwu, R. Recursis. https://recursis.app/.(accessed 16 09 2026).

[45] Shannon R. D., Prewitt C. T. (1970). Revised values of
effective ionic radii. Acta Crystallogr. B.

[46] Biffo, A. O. IonHop: NMR lineshape and relaxation analysis. IonHop. https://www.ionhop.com/.(accessed 16 09 2026)

[47] Ashbrook E.
S., McKay D. (2016). Combining
solid-state NMR spectroscopy with first-principles calculations
– a guide to NMR crystallography. Chem.
Commun..

[48] Waroquiers D. (2017). Statistical
Analysis of Coordination Environments in Oxides. Chem. Mater..

[49] Krauskopf T., Pompe C., Kraft M. A., Zeier W. G. (2017). Influence of Lattice
Dynamics on Na+ Transport in the Solid Electrolyte Na3PS4–xSex. Chem. Mater..

[50] Ngai K. L. (1998). Meyer–Neldel
rule and anti Meyer–Neldel rule of ionic conductivity: Conclusions
from the coupling model. Solid State Ion.

