# Supplementary material for: Activating Interstitial Oxygen Conduction in Sr3V2O8 Palmierite Oxides Through Minimal Mo-Substitution
Source: Chem Mater. 2026 Oct 13;38(21):11005–16. doi: 10.1021/acs.chemmater.6c01807 (PMC13757697; doi:10.1021/acs.chemmater.6c01807)
Supplement: Supplementary file 1 [file cm6c01807_si_001.pdf]

Activating Interstitial Oxygen Conduction in $Sr_3V_2O_8$ Palmierite Oxides through Minimal Mo Substitution – Supporting Information

List of Authors

Abdulkadir Olatunbosun Biffo [a], Anastasia K. Lavrinenko [a], Hailin Ma [a], Shengnan Zhang [a], Till Frömling [b], Alexandros Vasileiadis [a], Theodosios Famprikis [ad*], Pedro B. Groszewicz [ac*]

Affiliation

[*] Corresponding authors

[a] Department of Radiation Science and Technology, Delft University of Technology, Delft 2629JB, Netherlands

[b] Technical University of Darmstadt, Department of Materials and Earth Science, Darmstadt, Germany

[c] Helmholtz Zentrum Berlin für Materialien und Energie, Berlin 14109, Germany

[d] Inorganic Chemistry Laboratory, University of Oxford, Oxford OX1 3QR, United Kingdom

E-Mail:

theodosios.famprikis@chem.ox.ac.uk, p.groszewicz@tudelft.nl

Activating Interstitial Oxygen Conduction in Sr3V2O8 Palmierite Oxides through Minimal Mo Substitution .... **Error! Bookmark not defined.**

List of Authors 1

Affiliation 1

^{17}O Enrichment Process 6

Density Functional Theory 7

X-ray Diffraction and Lattice Parameters 10

ICP-MS 12

Electrochemical Impedance Spectroscopy (EIS) 13

Rietveld Refinement 19

^{51}V Solid-state Nuclear Magnetic Resonance 34

^{17}O Solid-state Nuclear Magnetic Resonance 37

Bond Valence Site Energy Calculations 39

References 43

Figure S1. Rhombohedral 1 × 2 × 1 $Sr_3VMoO_{8.5}$ supercells used for first-principles NMR calculations....7
Figure S2. (a) Plot of experimental ^{17}O chemical shift against calculated values for compounds from Table S1. (b) Radial distribution function (solid line) between V/Mo and O atoms and oxygen coordination number (dashed line) of V/Mo in $Sr_3VMoO_{8.5}$ from AIMD simulations at 600 K. (c) Average V/Mo-O bond length (blue dots) and ^{17}O chemical shift (orange dots) depending on polyhedra and oxygen crystallographic position....8
Figure S3. The evolution of (a) diffraction patterns, and (b) unit cell volume of the $Sr_3V_{2-x}Mo_xO_{8+x/2}$ series as a function of increasing Mo content....10
Figure S4. The evolution of the lattice parameters of the $Sr_3V_{2-x}Mo_xO_{8+x/2}$ ($0 \leq x \leq 0.4$) series with increasing x content. 11
Figure S5. The lin-KK residual plots of the representative impedance data for the $Sr_3V_{2-x}Mo_xO_{8+x/2}$ series ($0 \leq x \leq 0.2$) measured at 600 °C under O_2 gas 13
Figure S6. Evolution of bulk and grain-boundary permittivity of the $Sr_3V_{2-x}Mo_xO_{8+x/2}$ series ($0 \leq x \leq 0.2$) measured under O_2 gas as a function of inverse temperature....15
Figure S7. Evolution of bulk and grain-boundary RC-frequency of the $Sr_3V_{2-x}Mo_xO_{8+x/2}$ series ($0 \leq x \leq 0.2$) measured under O_2 gas as a function of inverse temperature. 16
Figure S8. The bulk, grain-boundary, and total conductivities of the $Sr_3V_{2-x}Mo_xO_{8+x/2}$ series ($0 \leq x \leq 0.2$) under O_2 gas as a function of inverse temperature....17
Figure S9. Comparison of the conductivities in the $Sr_3V_{2-x}Mo_xO_{8+x/2}$ series ($0 \leq x \leq 0.2$) series at 600 °C upon heating and cooling under O_2 gas. 18
Figure S10. The difference Fourier map analysis illustrating the static disorder on O1 (a-b) and O2 (b-c) sites and the need to split the sites into multiple folds to minimize the high atomic displacement parameter imposed by this static disorder in $Sr_3V_2O_8$. The (a) and (c) show the maps before applying site split, while (b) and (d) show the maps after the application of site split....19
Figure S11. The observation of the O2_s site within the proximity of the O2 site (a-b) in $Sr_3V_{1.8}Mo_{0.2}O_{8.1}$. The (a) shows the map before accounting for the O2_s site in the crystallographic model, while (b) shows the map after accounting for the O2_s site in the crystallographic model....19
Figure S12. Combined refinement of the $Sr_3V_{1.8}Mo_{0.2}O_{8.1}$ neutron and X-ray diffraction patterns (a) without including the O2_s site, and (b) with the inclusion of the O3 site....21
Figure S13. Rietveld refinement plot of the neutron pattern of $Sr_3V_2O_8$ recorded at 507 °C. .22
Figure S14. The Rietveld fit derived from the combined refinement of x-ray and neutron diffraction patterns of $Sr_3V_{1.9}Mo_{0.1}O_{8.05}$ recorded at room temperature. 23
Figure S15. Rietveld refinement plot of the neutron pattern of $Sr_3V_{1.9}Mo_{0.1}O_{8.05}$ recorded at 507 °C. 25
Figure S16. Rietveld refinement plot of the neutron pattern of $Sr_3V_{1.8}Mo_{0.2}O_{8.1}$ recorded at 507 °C. 29
Figure S17. The evolution of the relative percentage occupancy of the oxide ion sites across the $Sr_3V_{2-x}Mo_xO_{8+x/2}$ series ($0 \leq x \leq 0.2$) with increasing x content and temperature. 29
Figure S18. The refinement-derived crystallographic model of the $Sr_3V_{2-x}Mo_xO_{8+x/2}$ series ($0 \leq x \leq 0.2$). 30

Figure S19. Refinement of $Sr_3V_{1.8}Mo_{0.2}O_{8.1}$ neutron diffraction pattern at 4K. 32
Figure S20. Simulation of the ^{51}V MAS NMR spectra of the $Sr_3V_{2-x}Mo_xO_{8+x/2}$ series with CSA and CQ models. The simulations were performed using the MRsimulator Python package.[6] 34
Figure S21. The ^{51}V MAS NMR spectra of $Sr_3V_{2-x}Mo_xO_{8+x/2}$ series (x = 0.1 and 0.2) zoomed in on the center band, illustrating two isotropic chemical shifts at approximately -586 (brown) and 608 ppm (green), respectively. 36
Figure S22. The evolution of ^{17}O MAS NMR spectra of the $Sr_3V_{2-x}Mo_xO_{8+x/2}$ series ($0 \leq x \leq 0.2$) with increasing x at 11.7 T. It is worth noting that the plot illustrates the center band of the respective NMR spectrum. 37
Figure S23. $Sr_3V_2O_8$ (a) Bond-valence site energy landscape and (b) conduction map. $Sr_3V_{1.8}Mo_{0.2}O_{8.1}$ (c) BVSE migration energy landscape in the unconstrained model and (d) the conduction map. 39
Figure S24. BVSE-derived crystallographic structure illustrating how the interstitial sites transformed the isolated VO4 units into isolated corner-sharing polyhedra with mixed coordination geometries separated by octahedral M2 vacancies. 41
Figure S25. Meyer-Nédel (M-N) compensation plot contrasting a schematic M-N relationship (green line) with experimental data (brown data points). An energy landscape diagram illustrating where the migration barrier height remains constant across different compositions, while the pre-exponential factor varies with carrier density and available pathways rather than with barrier geometry. The carrier density is slightly overestimated, as incorporating x amount of Molybdenum into the palmarite structure introduces x/2 charge-compensating oxygen per formula unit ($Sr_3V_{2-x}Mo_xO_{8+x/2}$). 42

Table S1. Experimental and calculated values of ^{17}O chemical shift for reference compounds. 9
Table S2. Nominal and ICP-MS determined compositions of $Sr_3V_{2-x}Mo_xO_{8+x/2}$ series ($0 \leq x \leq 0.2$). 12
Table S3. The crystallographic information of $Sr_3V_2O_8$ derived from the combined refinement of X-ray and neutron diffraction powder patterns at room temperature. 20
Table S4. The crystallographic information derived from the refinement of the neutron pattern of $Sr_3V_2O_8$ recorded at 507 °C. 23
Table S5. The crystallographic information of $Sr_3V_{1.9}Mo_{0.1}O_{8.05}$ derived from the combined refinement of X-ray and neutron diffraction powder patterns at room temperature. 24
Table S6. The crystallographic information derived from the refinement of the neutron pattern of $Sr_3V_{1.9}Mo_{0.1}O_{8.05}$ recorded at 507 °C. 26
Table S7. The crystallographic information of $Sr_3V_{1.8}Mo_{0.2}O_{8.1}$ derived from the combined refinement of X-ray and neutron diffraction powder patterns at room temperature with the inclusion of O2_s in the crystallographic model 27
Table S8. The crystallographic information of $Sr_3V_{1.8}Mo_{0.2}O_{8.1}$ was derived from the combined refinement of X-ray and neutron diffraction powder patterns at room temperature without the inclusion of O2_s in the crystallographic model 28
Table S9. The crystallographic information derived from the neutron diffraction pattern of $Sr_3V_{1.8}Mo_{0.2}O_{8.1}$, recorded at 507 °C. 31
Table S10. The crystallographic information derived from the neutron diffraction pattern of $Sr_3V_{1.8}Mo_{0.2}O_{8.1}$, recorded at 4 K. 33
Table S11. A table of the optimized parameters obtained from the modeling of the ^{51}V MAS NMR spectra across the $Sr_3V_{2-x}Mo_xO_{8+x/2}$ series ($0 \leq x \leq 0.2$). 35
Table S12. Estimation of the percentage area of the two isotropic chemical shifts observed in the center band of the ^{51}V MAS NMR spectra of the $Sr_3V_{2-x}Mo_xO_{8+x/2}$ series ($0.1 \leq x \leq 0.2$). 35
Table S13. ^{17}O NMR peak parameters for $Sr_3V_{2-x}Mo_xO_{8+x/2}$ series ($0 \leq x \leq 0.2$) at 11.7T. 38
Table S14. ^{17}O NMR peak parameters for $Sr_3V_{2-x}Mo_xO_{8+x/2}$ series ($0 \leq x \leq 0.2$) at 20T. 38

^{17}O Enrichment Process

The setup comprises a network of gas-tight metallic pipes equipped with precision control valves connected to a pressurized cylinder containing oxygen-17 gas. The central component of the experimental setup is a two—perpendicular—outlet system, connected by a valve, which incorporates an oven-integrated sample chamber (ampoule) and a vacuum pump system for environmental control, respectively. The enrichment process begins by connecting the sample chamber to a vacuum pump at its designated outlets, then evacuating the chamber. Once the desired vacuum level is achieved, the chamber is completely sealed by closing the sample-vacuum valve to maintain the controlled environment. Following this step, oxygen-17 gas is carefully introduced into the chamber with the gas pressure precisely regulated to 0.15 atmospheres. The oven is then operated using thermal treatment protocols with an initial phase where temperature is increased from ambient conditions to 900 °C at a controlled ramp rate of 87.5 $Kmin^{-1}$, followed by maintaining 900 °C temperature for an extended period of 3 days to ensure complete isotopic exchange, and finally cooling the temperature gradually to room temperature at a controlled rate of 5 $Kmin^{-1}$.

Density Functional Theory

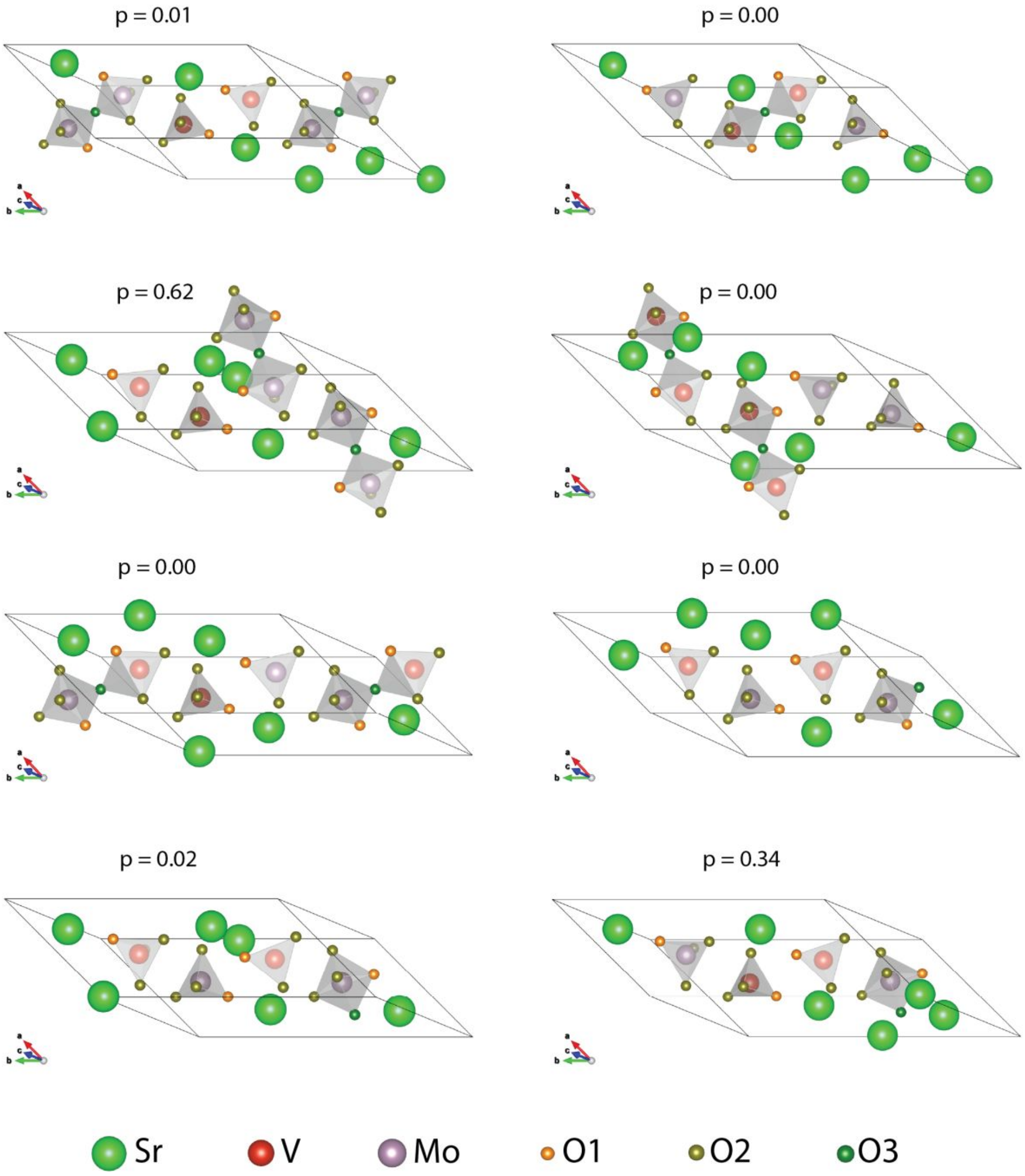

Figure S1. Rhombohedral 1 × 2 × 1 $Sr_3VMoO_{8.5}$ supercells used for first-principles NMR calculations, with p indicating the Boltzmann weight of each configuration at the synthesis temperature of 1373 K.

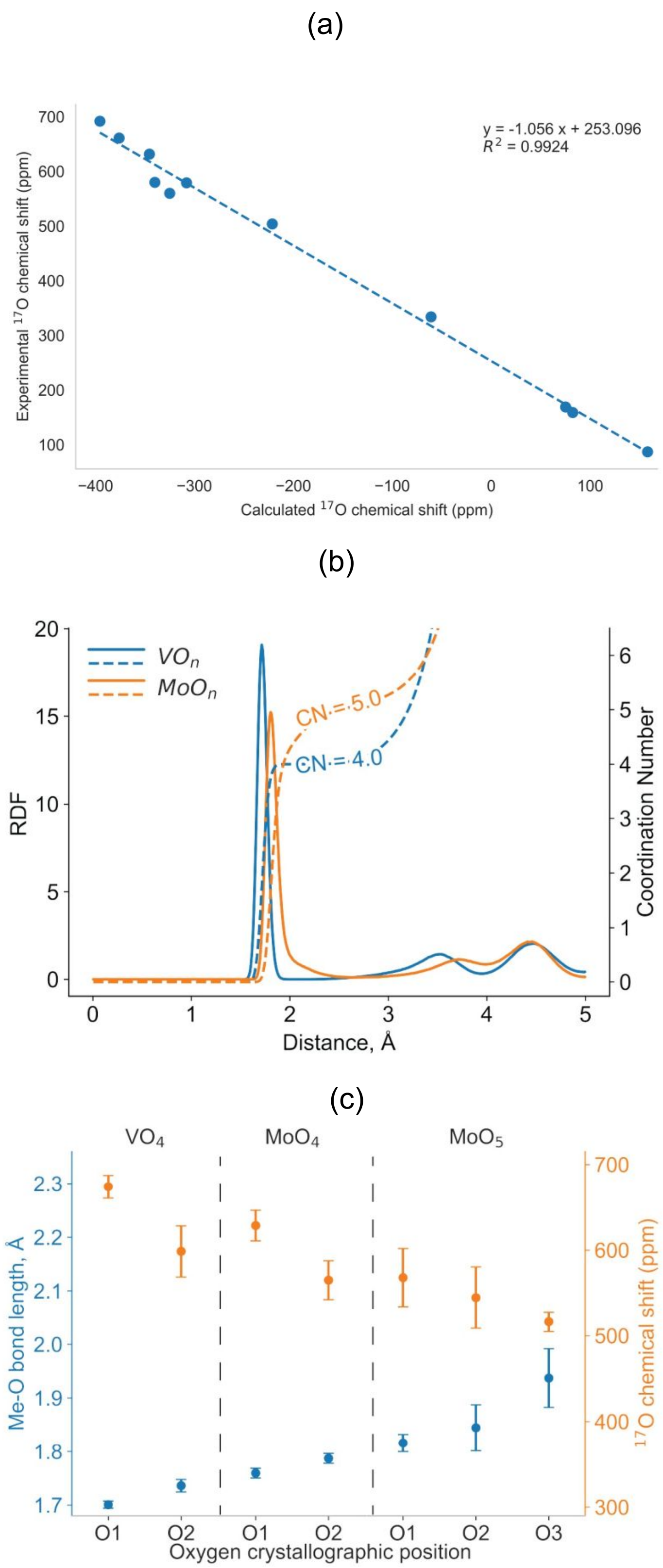

Figure S2. (a) Plot of experimental ^{17}O chemical shift against calculated values for compounds from Table S1. (b) Radial distribution function (solid line) between V/Mo and O atoms and oxygen coordination number (dashed line) of V/Mo in $Sr_3VMoO_{8.5}$ from AIMD simulations at 600 K. (c) Average V/Mo-O bond length (blue dots) and ^{17}O chemical shift (orange dots) depending on polyhedra and oxygen crystallographic position.

Table S1. Experimental and calculated values of ^{17}O chemical shift for reference compounds.

Compound	Calculated ^{17}O chemical shift	Experimental ^{17}O chemical shift
$Ba_3V_2O_8$	-395.57	691 [this work]
Ba_3V_2O8	-345.54	631 [this work]
$Sr_3V_2O_8$	-376.13	661 [this work]
$Sr_3V_2O_8$	-308.00	579 [this work]
$LiNbO_3$	-221.32	504[1]
BaO_2	-60.85	334[2]
$BaSiO_3$	75.19	169[2]
$BaSiO_3$	82.38	159[2]
$BaSiO_3$	158.04	87[2]
$Sc_2(MoO_4)_3$	-340.00	580[3]
$Sc_2(MoO_4)_3$	-325.00	560[3]

X-ray Diffraction and Lattice Parameters

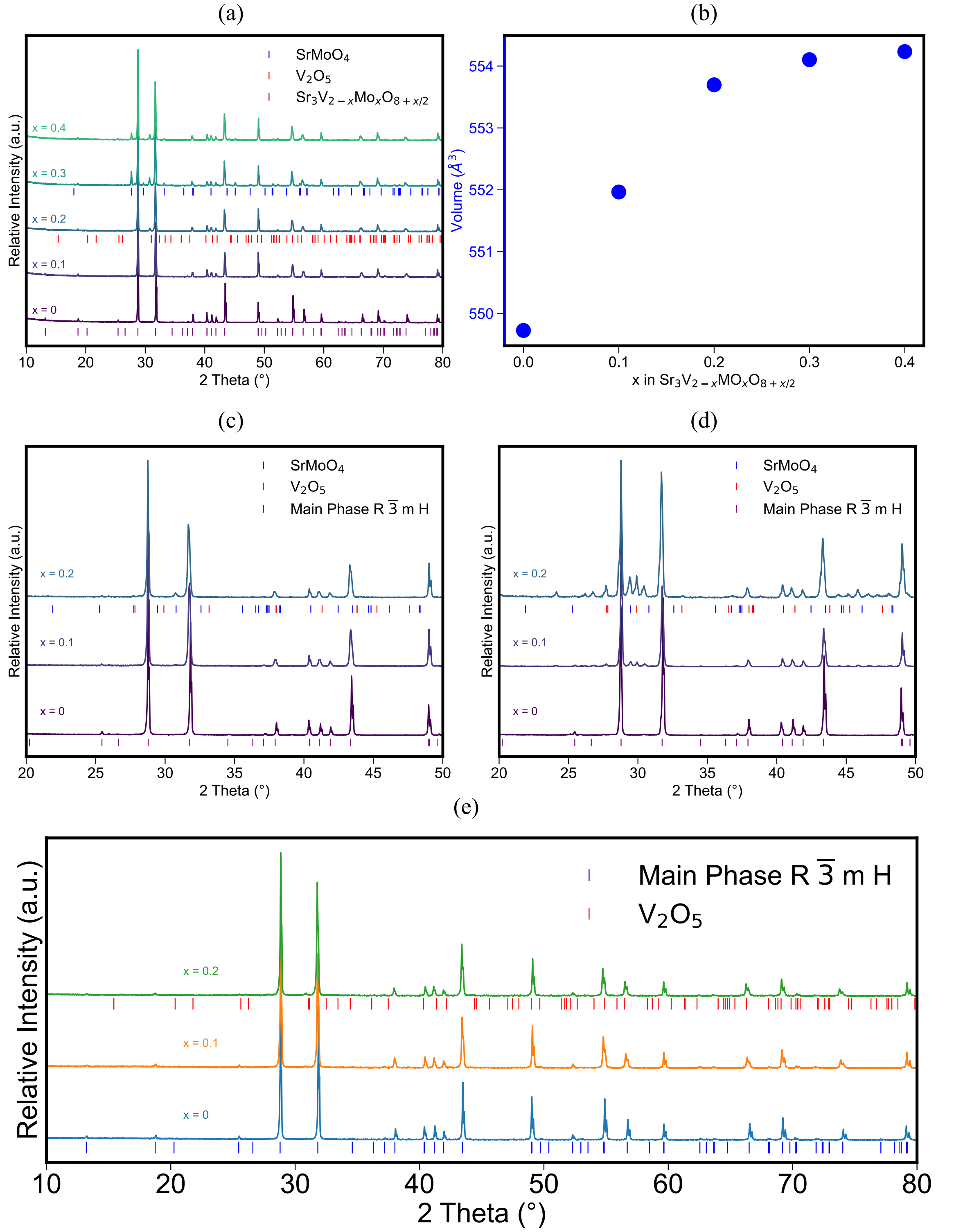

Figure S3. The evolution of (a) diffraction patterns, and (b) unit cell volume of the $Sr_3V_{2\text{-}x}Mo_xO_{8+x/2}$ series as a function of increasing Mo content. (c) and (d) illustrate the diffraction patterns recorded before and after ^{17}O enrichment of the $Sr_3V_{2\text{-}x}Mo_xO_{8+x/2}$ ($0 \leq x \leq 0.2$) compositions. (e) Diffraction patterns of sintered pellets of $Sr_3V_{2\text{-}x}Mo_xO_{8+x/2}$ ($0 \leq x \leq 0.2$) used for impedance measurements.

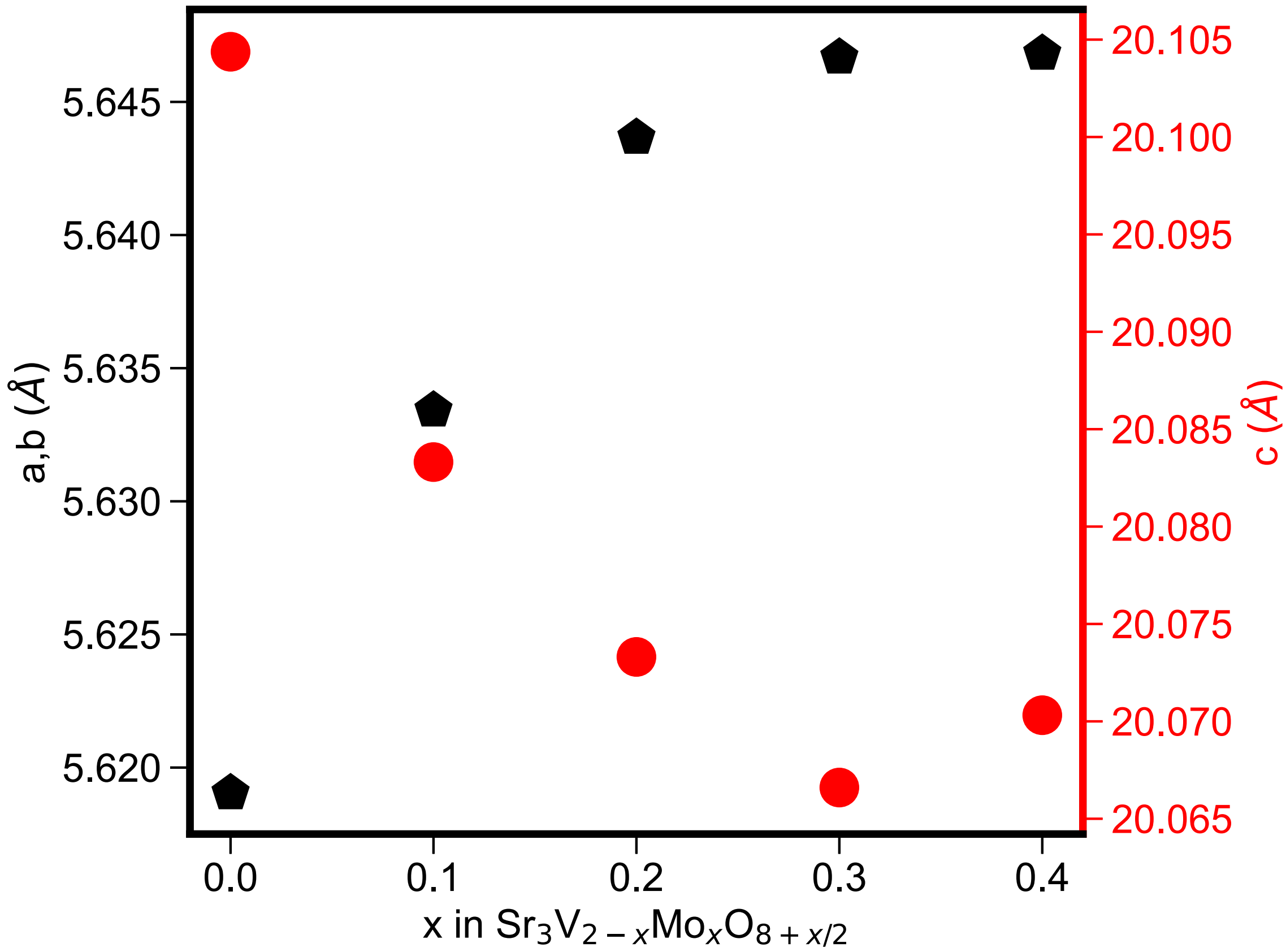

Figure S4. The evolution of the lattice parameters of the $Sr_3V_{2-x}Mo_xO_{8+x/2}$ ($0 \leq x \leq 0.4$) series with increasing x content.

ICP-MS

Table S2. Nominal and ICP-MS determined compositions of $Sr_3V_{2-x}Mo_xO_{8+x/2}$ series ($0 \leq x \leq 0.2$).

Nominal composition	*Element*	*Nominal Stoichiometry*	*A-site normalization*	*Deviation (%)*	*B-site normalization*	*Deviation (%)*
$Sr_3V_2O_8$	Sr<br>V	3<br>2	3.000<br>2.044	0.0<br>+2.2	2.936<br>2	-2.1<br>0
$Sr_3V_{1.9}Mo_{0.1}O_{8.05}$	Sr<br>V<br>Mo	3<br>1.9<br>0.1	3.000<br>1.971<br>0.131	0.0<br>+3.7<br>+30.8	2.854<br>1.876<br>0.124	-4.9<br>-1.3<br>+24.0
$Sr_3V_{1.8}Mo_{0.2}O_{8.1}$	Sr<br>V<br>Mo	3<br>1.8<br>0.2	3.000<br>1.918<br>0.197	0.0<br>+6.5<br>-1.5	2.837<br>1.813<br>0.187	-5.4<br>+0.7<br>-6.5

The cation stoichiometries, normalized to the A- (Sr = 3) and B-site (V + Mo = 2), showed V and Mo content within ~ ≤ 7 % of targeted values, except for the low Mo-substitution (x = 0.1), where a large deviation within the range of 24 and 31 % for the Mo composition reflects uncertainty in quantifying low Mo concentration.

Electrochemical Impedance Spectroscopy (EIS)

This section describes the validation of the EIS spectra using Schönleber *et al.*'s linear Kramers-Kronig (lin-KK) method embedded in the impedance.py Python package.[4,5] Furthermore, the modeling of EIS spectra using Randles' series R-CPE circuits is highlighted.

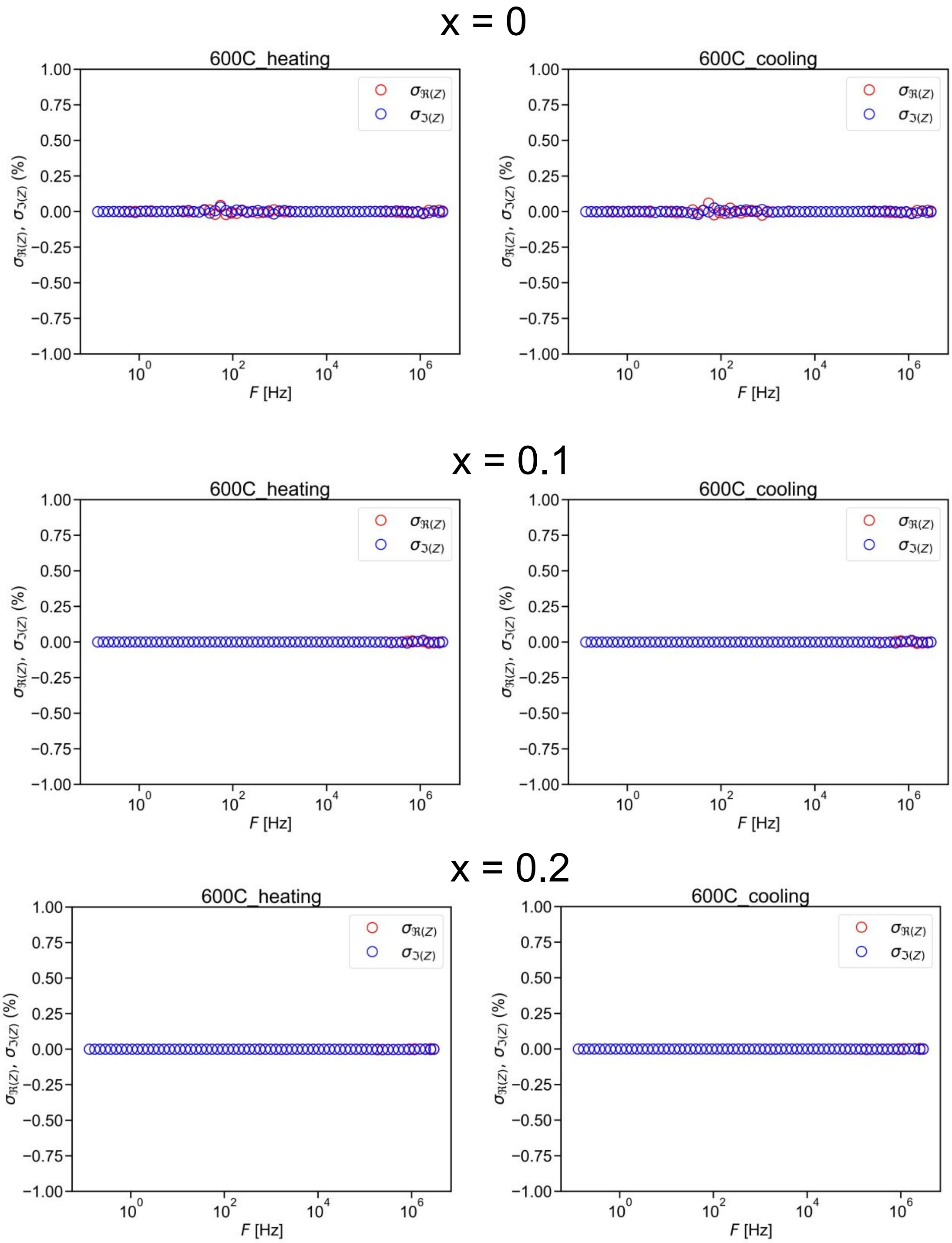

Figure S5. The lin-KK residual plots of the representative impedance data for the $Sr_3V_{2-x}Mo_xO_{8+x/2}$ series ($0 \leq x \leq 0.2$) measured at 600 °C under O_2 gas

Figure S5 and S15 illustrate the lin-KK residual plots of the representative impedance data of the $Sr_3V_{2-x}Mo_xO_{8+x/2}$ series ($0 \leq x \leq 0.2$) measured at 600 °C under O_2 gas. The residuals are randomly scattered across the frequency range of 100 mHz to 3 MHz, indicating that the collected impedance data are KK-compliant (i.e., valid) and can be modeled for data analysis.

The impedance spectra were modeled using two or three R-CPE equivalent circuit elements connected in series. From this modeling approach, distinct CPE values were extracted from the impedance spectra at different frequency regions, which were subsequently converted to permittivity (ε) to accurately assign the R-CPE semicircle to a phenomenon occurring in the electrolyte.

$$\varepsilon = \frac{t}{A} \times \frac{(R \times CPE)^{\frac{1}{n}}}{R} \quad \textit{Equation S 1}$$

where ε is the permittivity in F cm^{-1}, t is the thickness of the symmetrical cell in cm, A is the area of the cell in cm^2, R and CPE are the resistance (Ω) and the constant phase element of the R-CPE equivalent circuit, respectively, and n is the constant phase between 0 and 1.

For example, the impedance spectra at 600 °C under dry air exhibited ε values of ~5 $pFcm^{-1}$, and ~0.1 $nFcm^{-1}$ at frequencies of ~3 MHz (high frequency) and ~62 kHz (mid frequency), respectively. The high- and mid-frequency processes were assigned to bulk and grain boundary charge-transfer processes, respectively. This approach was carefully applied to all recorded impedance spectra. The evolution of these charge-transfer processes as a function of inverse temperature is illustrated in Figure S6.

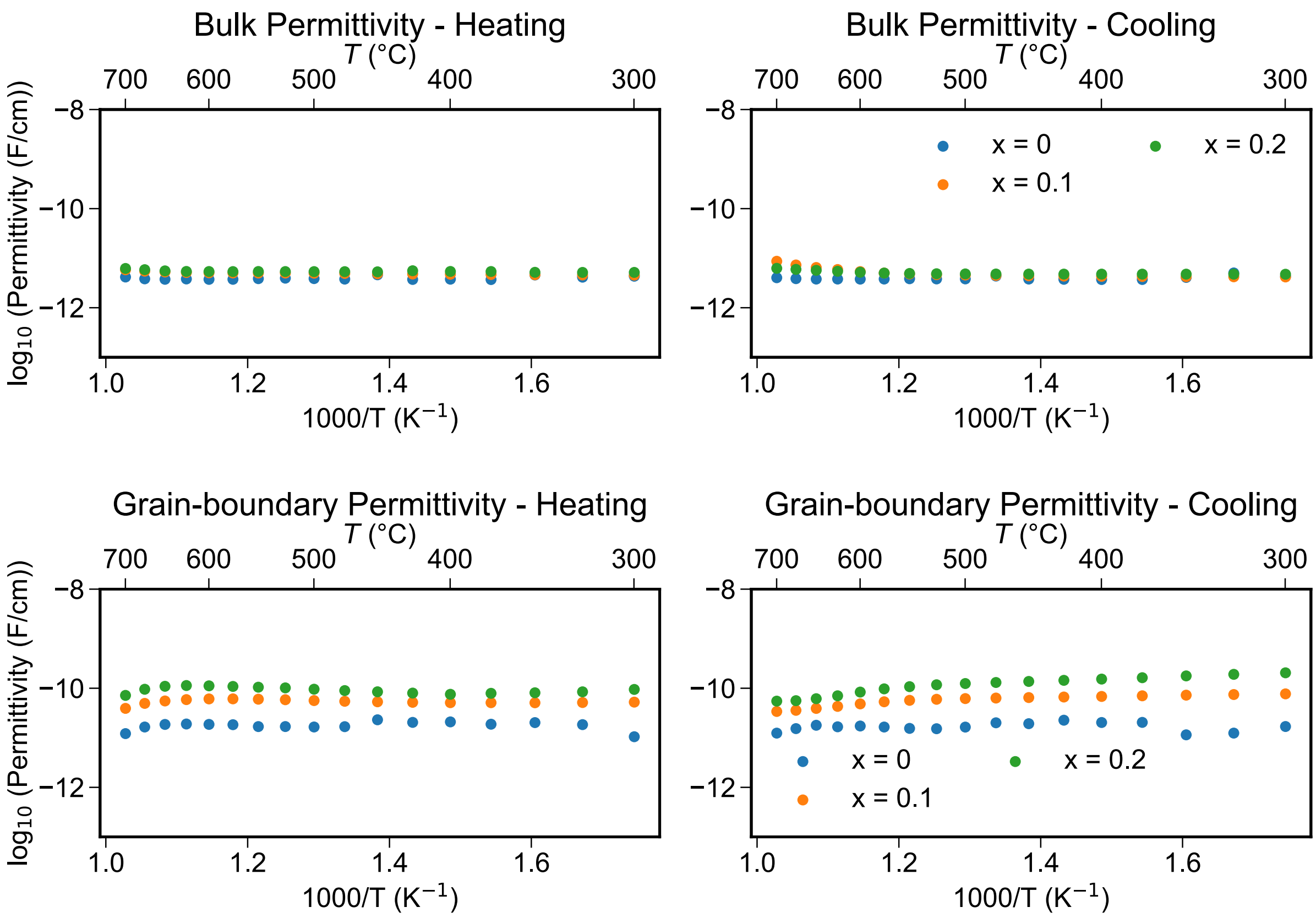

Figure S6. Evolution of bulk and grain-boundary permittivity of the $Sr_3V_{2-x}Mo_xO_{8+x/2}$ series ($0 \leq x \leq 0.2$) measured under O_2 gas as a function of inverse temperature.

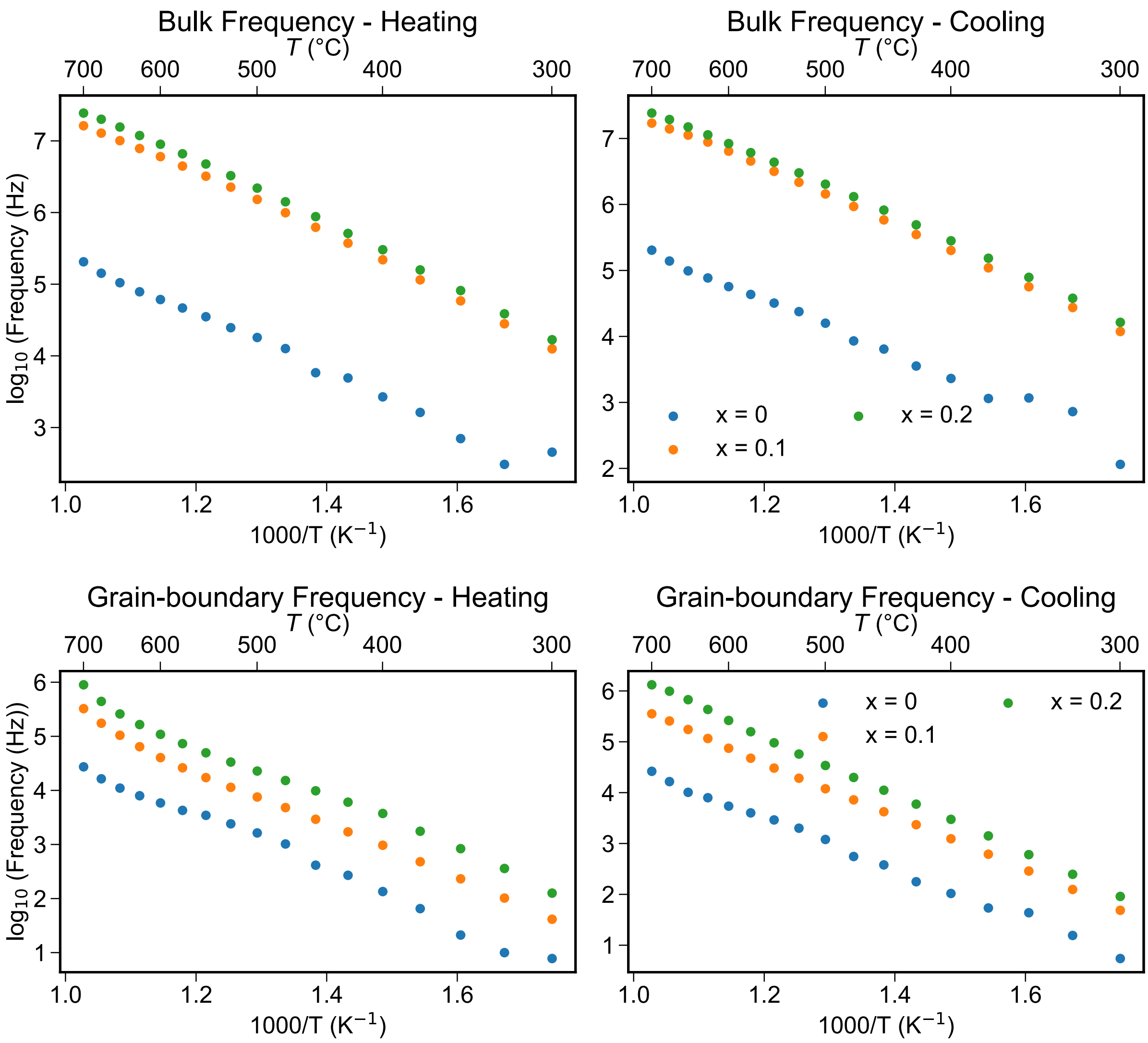

Figure S7. Evolution of bulk and grain-boundary RC-frequency of the $Sr_3V_{2-x}Mo_xO_{8+x/2}$ series ($0 \leq x \leq 0.2$) measured under O_2 gas as a function of inverse temperature.

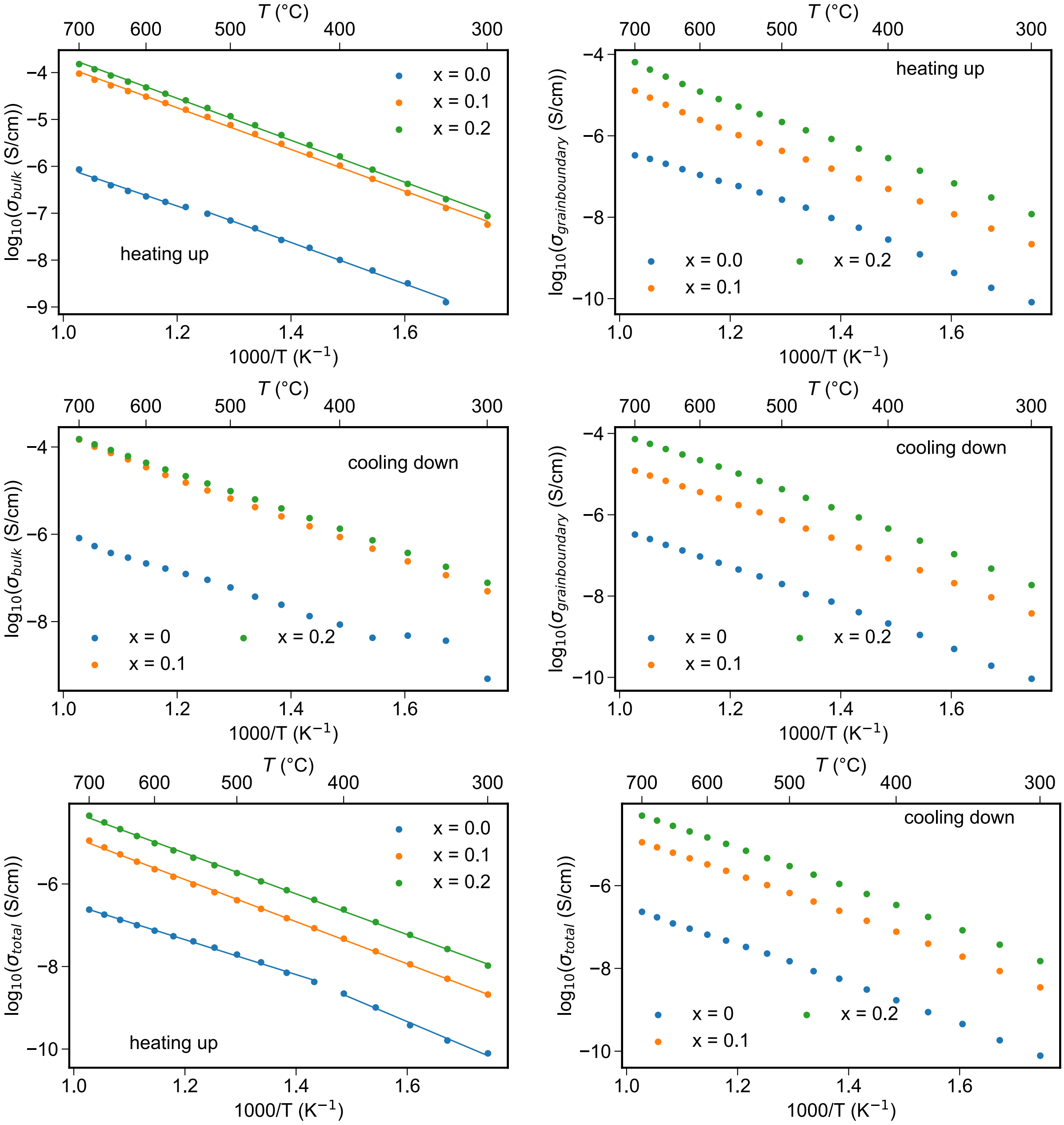

Figure S8. The bulk, grain-boundary, and total conductivities of the $Sr_3V_{2-x}Mo_xO_{8+x/2}$ series ($0 \leq x \leq 0.2$) under O_2 gas as a function of inverse temperature.

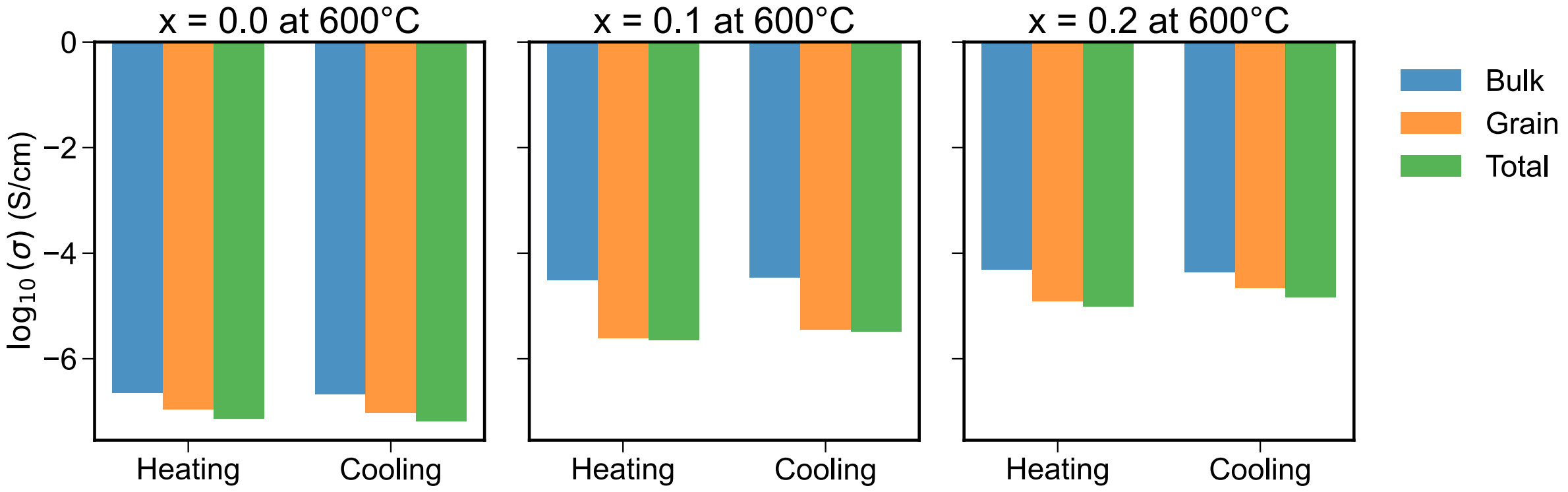

Figure S9. Comparison of the conductivities in the $Sr_3V_{2-x}Mo_xO_{8+x/2}$ series ($0 \leq x \leq 0.2$) series at 600 °C upon heating and cooling under O_2 gas.

Rietveld Refinement

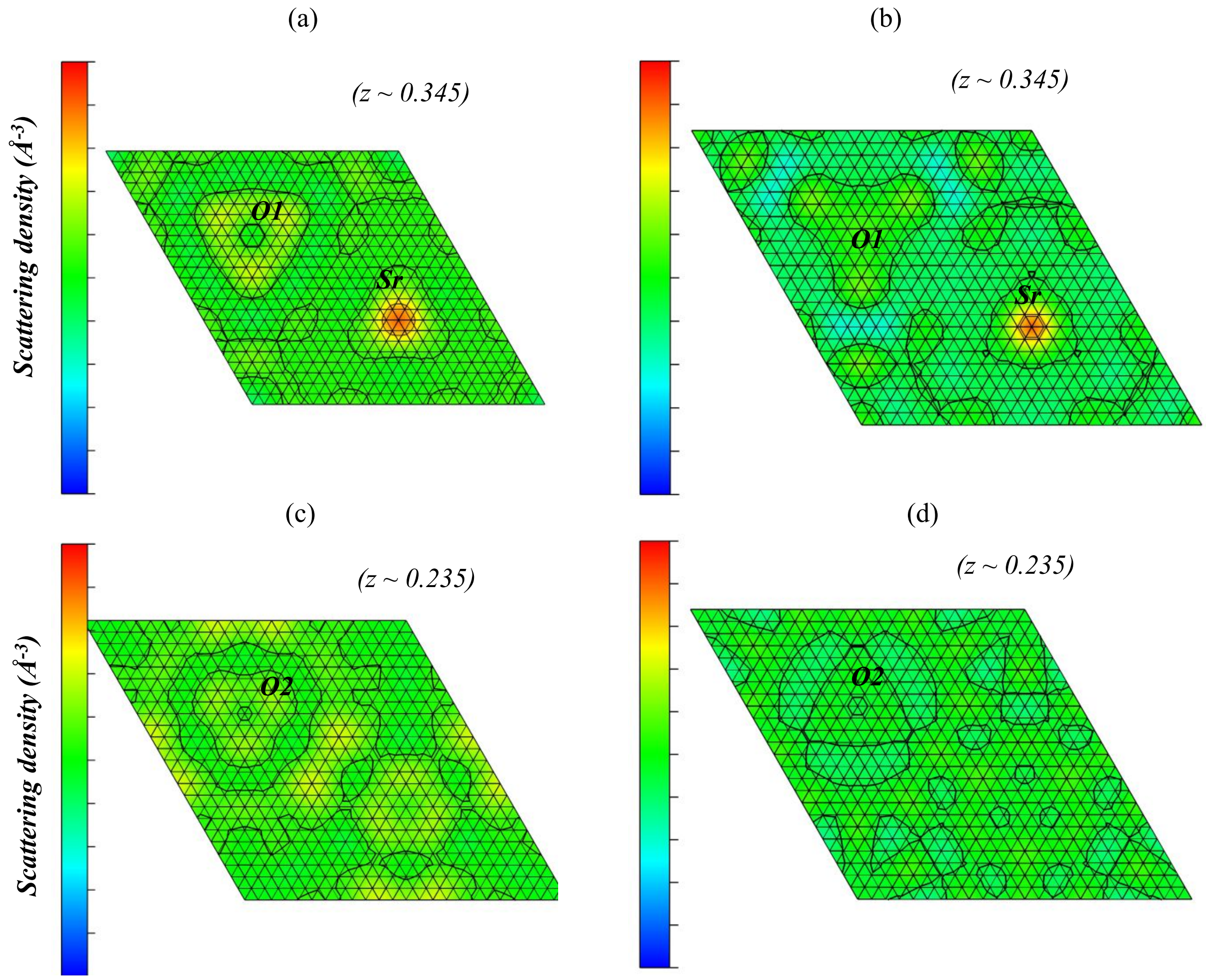

Figure S10. The difference Fourier map analysis illustrating the static disorder on O1 (a-b) and O2 (b-c) sites and the need to split the sites into multiple folds to minimize the high atomic displacement parameter imposed by this static disorder in $Sr_3V_2O_8$. The (a) and (c) show the maps before applying site split, while (b) and (d) show the maps after the application of site split.

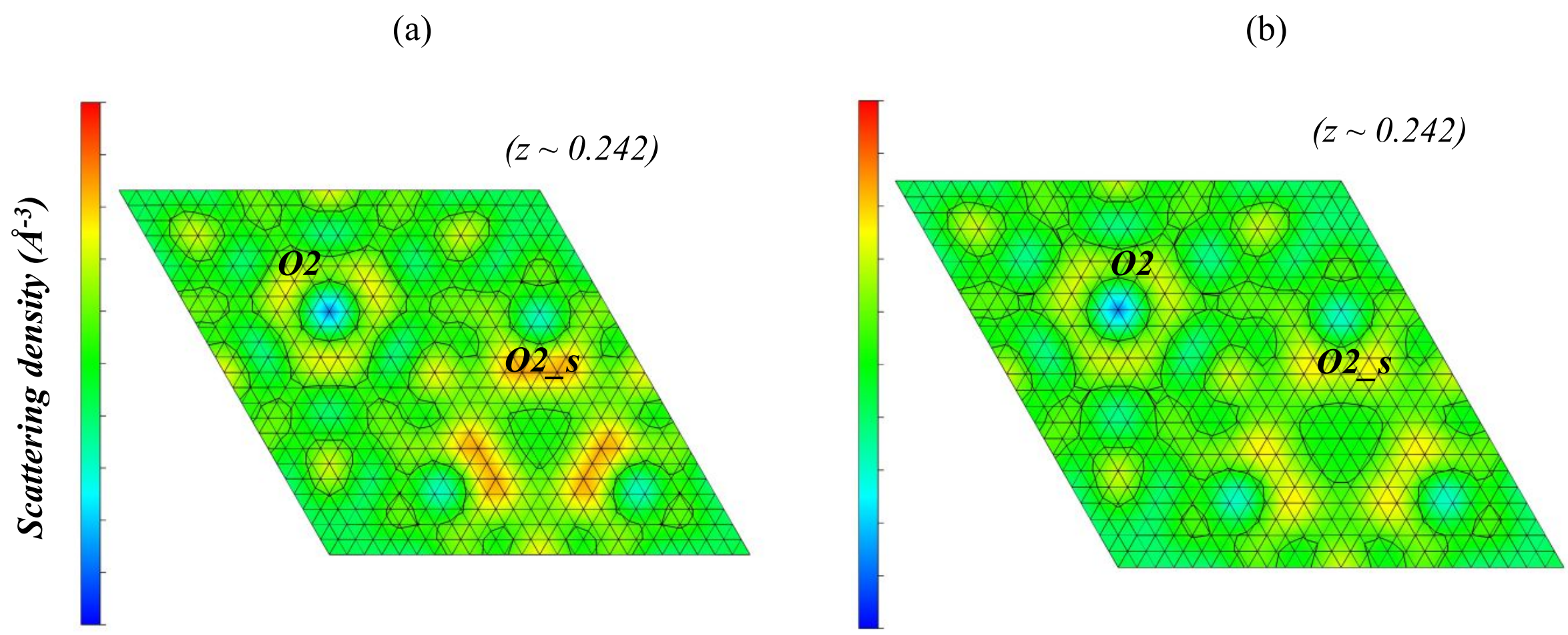

Figure S11. The observation of the O2_s site within the proximity of the O2 site (a-b) in $Sr_3V_{1.8}Mo_{0.2}O_{8.1}$. The (a) shows the map before accounting for the O2_s site in the crystallographic model, while (b) shows the map after accounting for the O2_s site in the crystallographic model.

Table S3. The crystallographic information of $Sr_3V_2O_8$ derived from the combined refinement of X-ray and neutron diffraction powder patterns at room temperature.

Space group	R-3m				X-ray	Neutron	
a,b (Å)	5.61998(7)	wR (%)			7.08	6.35	
c (Å)	20.1073(2)	overall combined weighted residual (Rwp) = 6.8 %					
α, β (°)	90						
γ (°)	120						
Volume ($Å^3$)	549.989(8)						
label	Element	Multiplicity	x	y	z	Frac.	U_{iso}
Sr1	Sr	6	0	0	0.202219(9)	1	0.0030(3)
Sr2	Sr	3	0	0	0	1	0.0079(5)
V1	V	6	0	0	0.40825(2)	1	0.014(2)
O1	O	36	0.043(1)	0.036(2)	0.3225(1)	0.167	0.0032(9)
O2	O	36	0.5267(5)	0.5295(4)	0.23171(9)	0.5	0.0062(4)

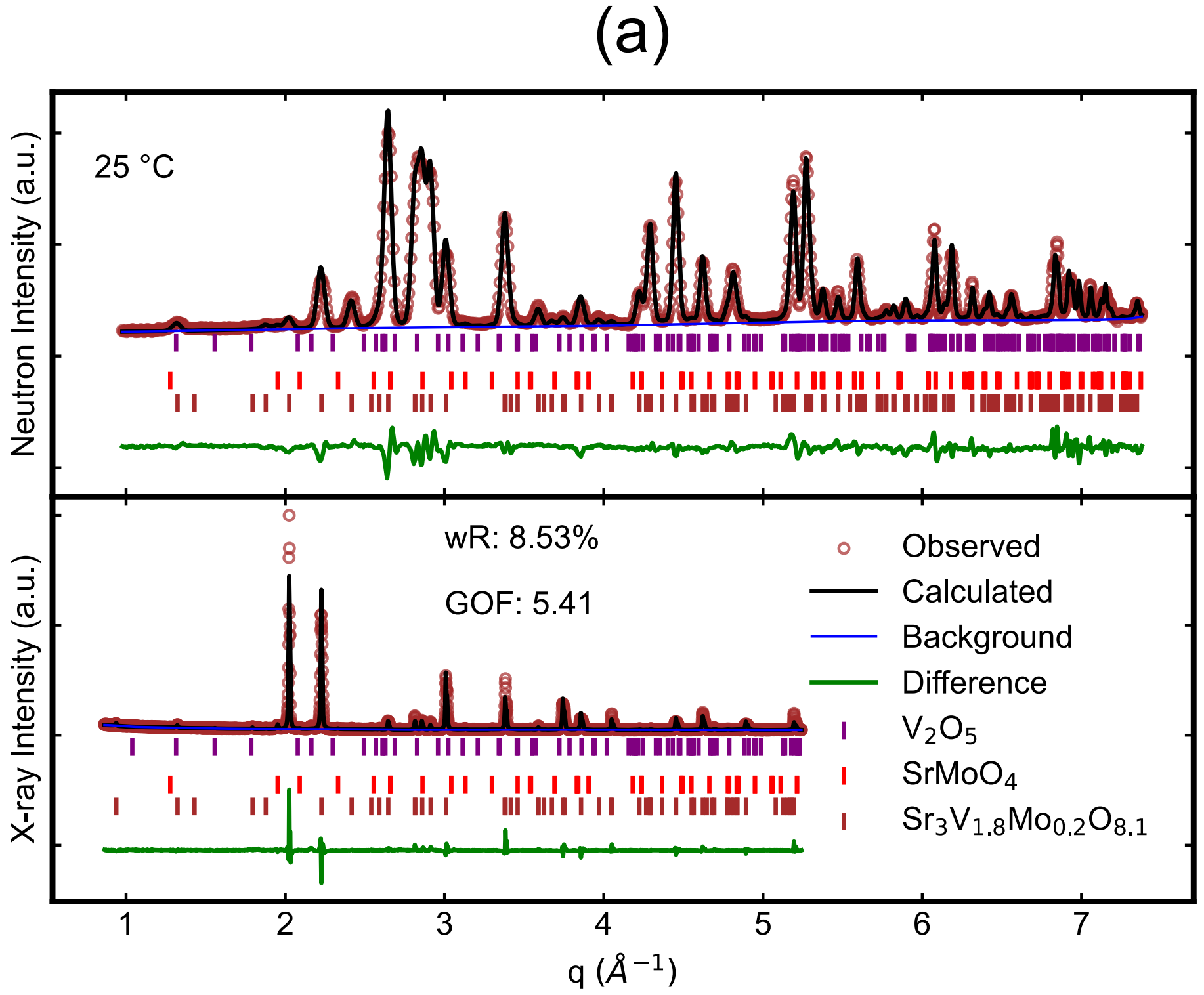

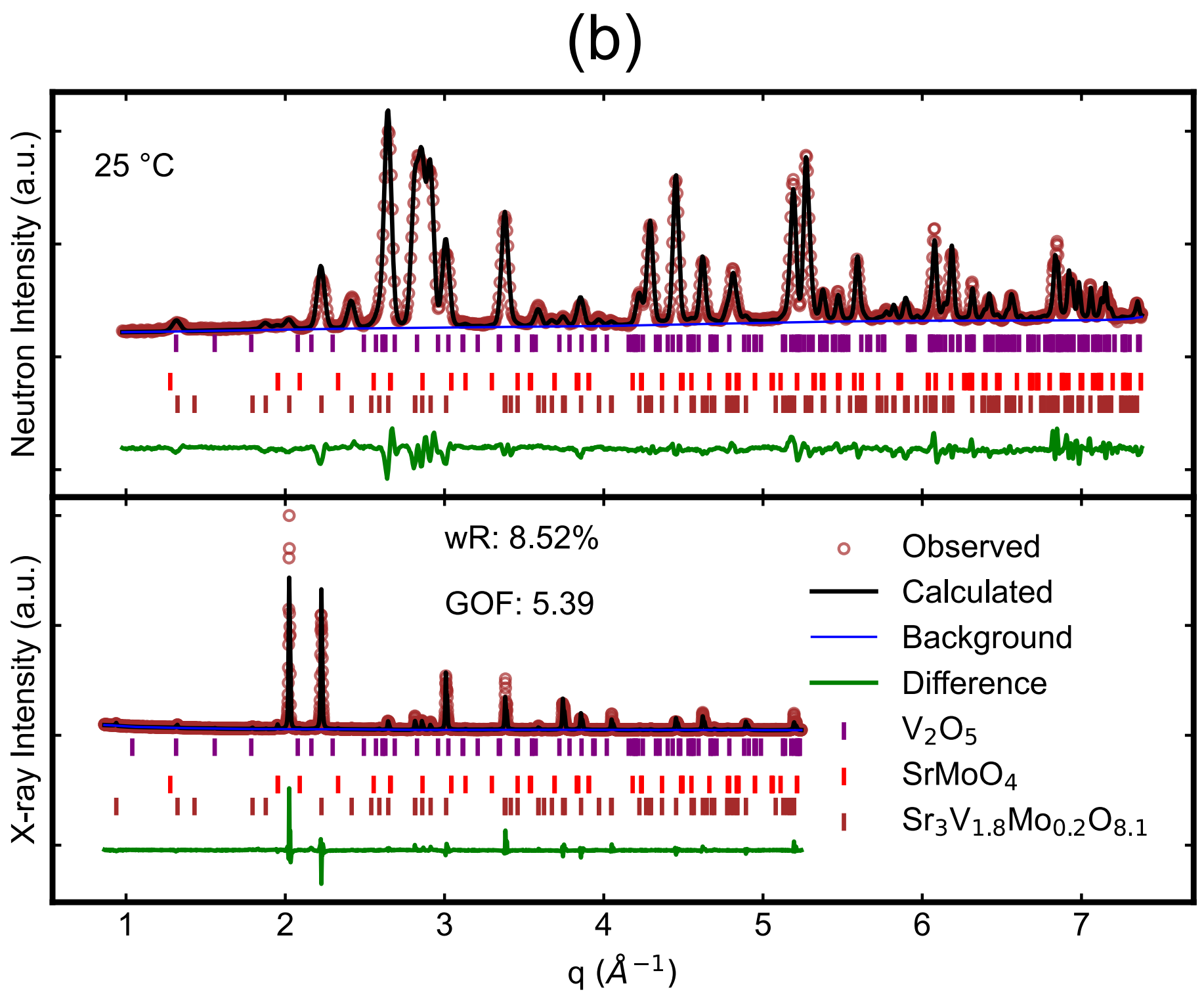

Figure S12. Combined refinement of the $Sr_3V_{1.8}Mo_{0.2}O_{8.1}$ neutron and X-ray diffraction patterns (a) without including the O2_s site, and (b) with the inclusion of the O3 site.

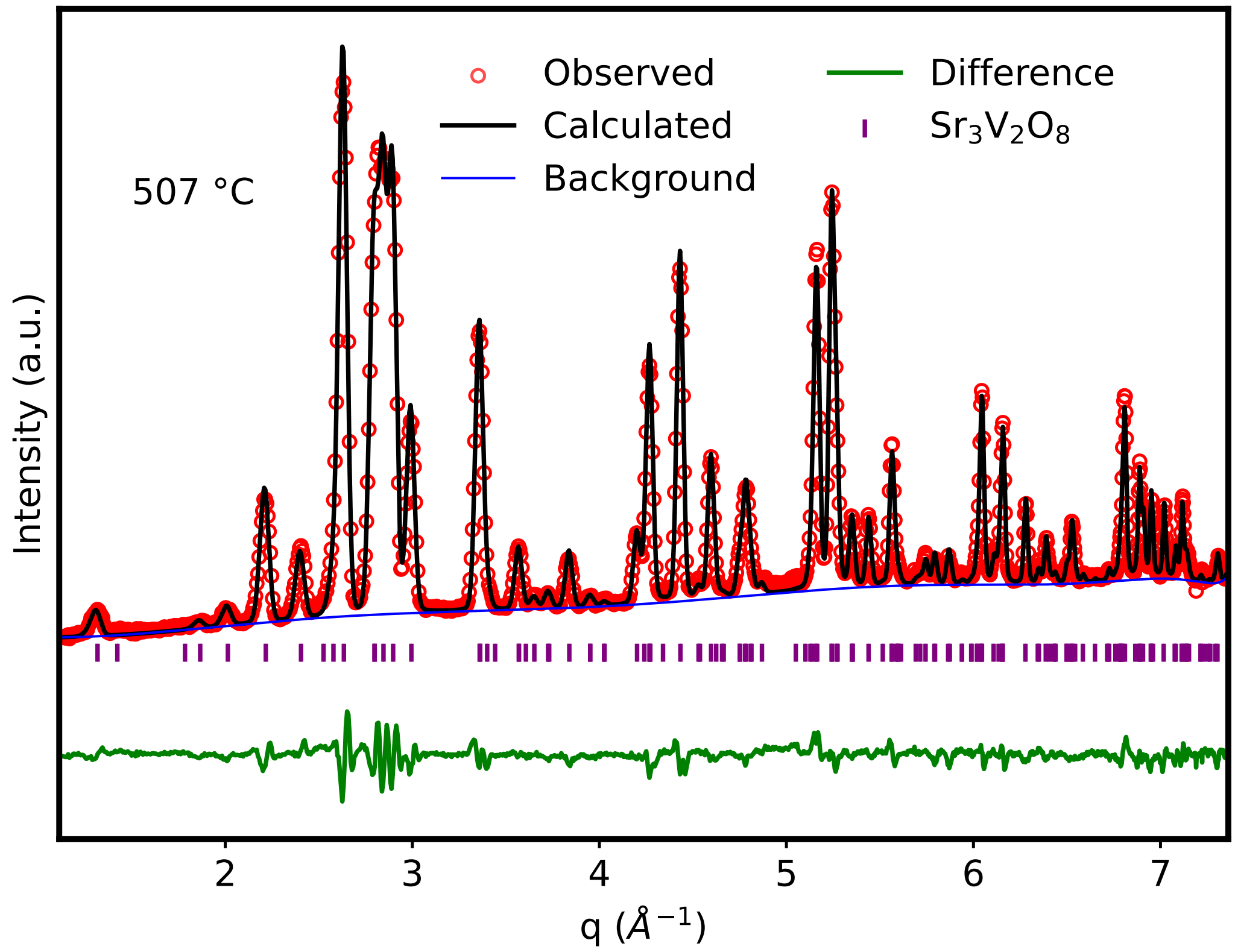

Figure S13. Rietveld refinement plot of the neutron pattern of $Sr_3V_2O_8$ recorded at 507 °C.

Table S4. The crystallographic information derived from the refinement of the neutron pattern of $Sr_3V_2O_8$ recorded at 507 °C.

Space group	R-3m				Neutron		
a,b (Å)	5.66928(3)			wR (%)	4.17		
c (Å)	20.2048(7)						
α, β (°)	90						
γ (°)	120						
Volume (Å³)	562.394(3)						
label	Element	Multiplicity	x	y	z	Frac.	Uiso
Sr1	Sr	6	0	0	0.20275(1)	1	0.0211(4)
Sr2	Sr	3	0	0	0	1	0.0211(4)
V1	V	6	0	0	0.4053(2)	1	0.0211(4)
O1	O	36	0.0561(1)	0.0081(2)	0.32284(2)	0.16(1)	0.0083(1)
O2	O	36	0.5271(6)	0.5321(6)	0.23255(1)	0.507(1)	0.0192(6)

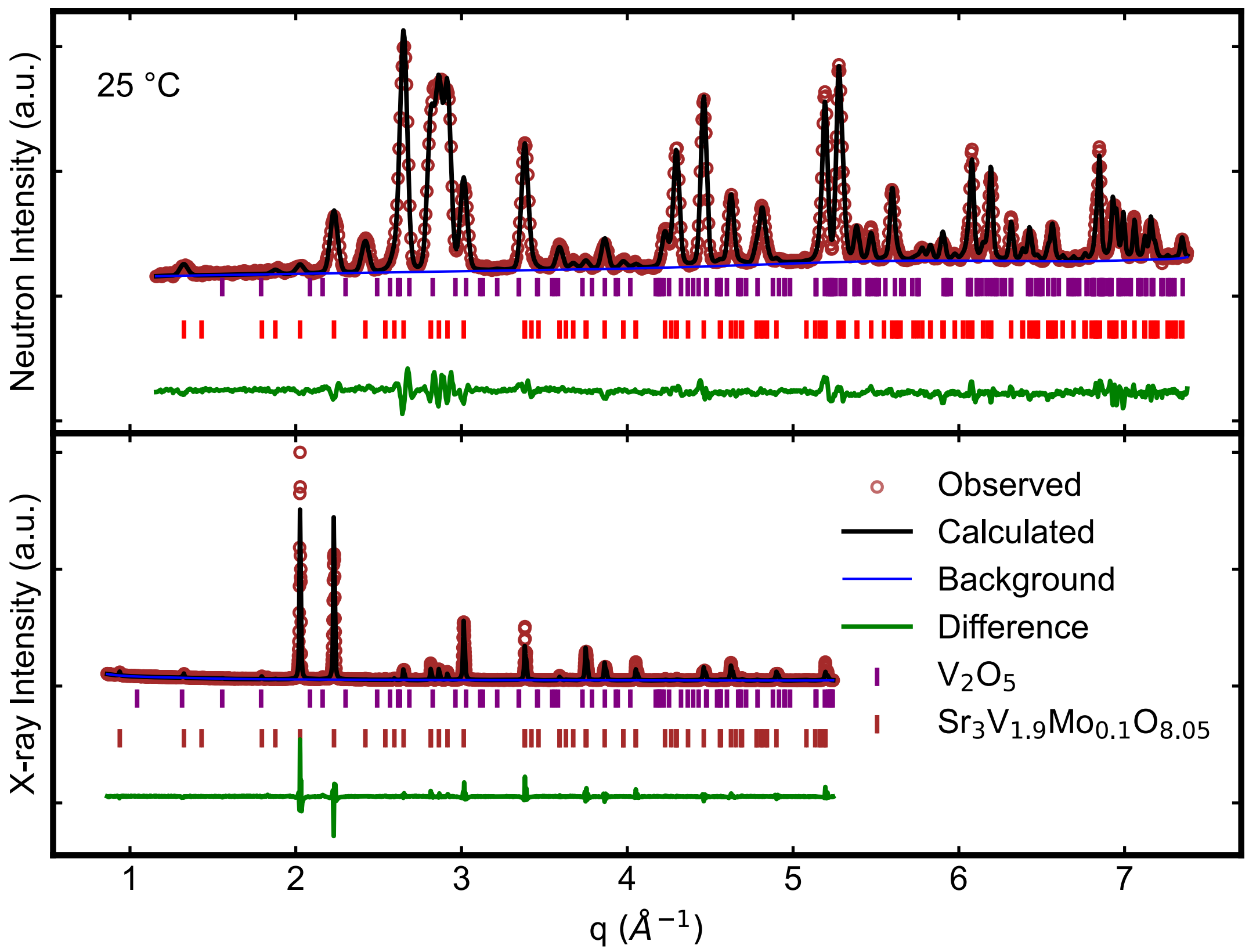

Figure S14. The Rietveld fit derived from the combined refinement of x-ray and neutron diffraction patterns of $Sr_3V_{1.9}Mo_{0.1}O_{8.05}$ recorded at room temperature.

Table S5. The crystallographic information of $Sr_3V_{1.9}Mo_{0.1}O_{8.05}$ derived from the combined refinement of X-ray and neutron diffraction powder patterns at room temperature.

Space Group	R-3m			X-ray	Neutron		
a,b (Å)	5.63346(2)		wR(%)	9.88	6.35		
c (Å)	20.0871(4)						
αβ (°)	90						
γ (°)	120						
Volume (Å^3)	552.075(2)						
Label	Element	Multiplicity	x	y	z	Frac.	Uiso
Sr1	Sr	6	0	0	0.20231(2)	1	0.007(5)
Sr2	Sr	3	0	0	0	1	0.007(5)
Mo1	Mo	6	0	0	0.40567(3)	0.05	0.0059(2)
V1	V	6	0	0	0.40567(3)	0.95	0.0059(2)
O1	O	36	0.043(3)	0.029(6)	0.32184(3)	0.16	0.007(7)
O2	O	36	0.5301(9)	0.5327(9)	0.23215(2)	0.511	0.007(7)
	$Sr_3V_{1.9}Mo_{0.1}O_{8.05}$	V_2O_5					
% composition	98.54	1.46					
% error	0.24	0.24					

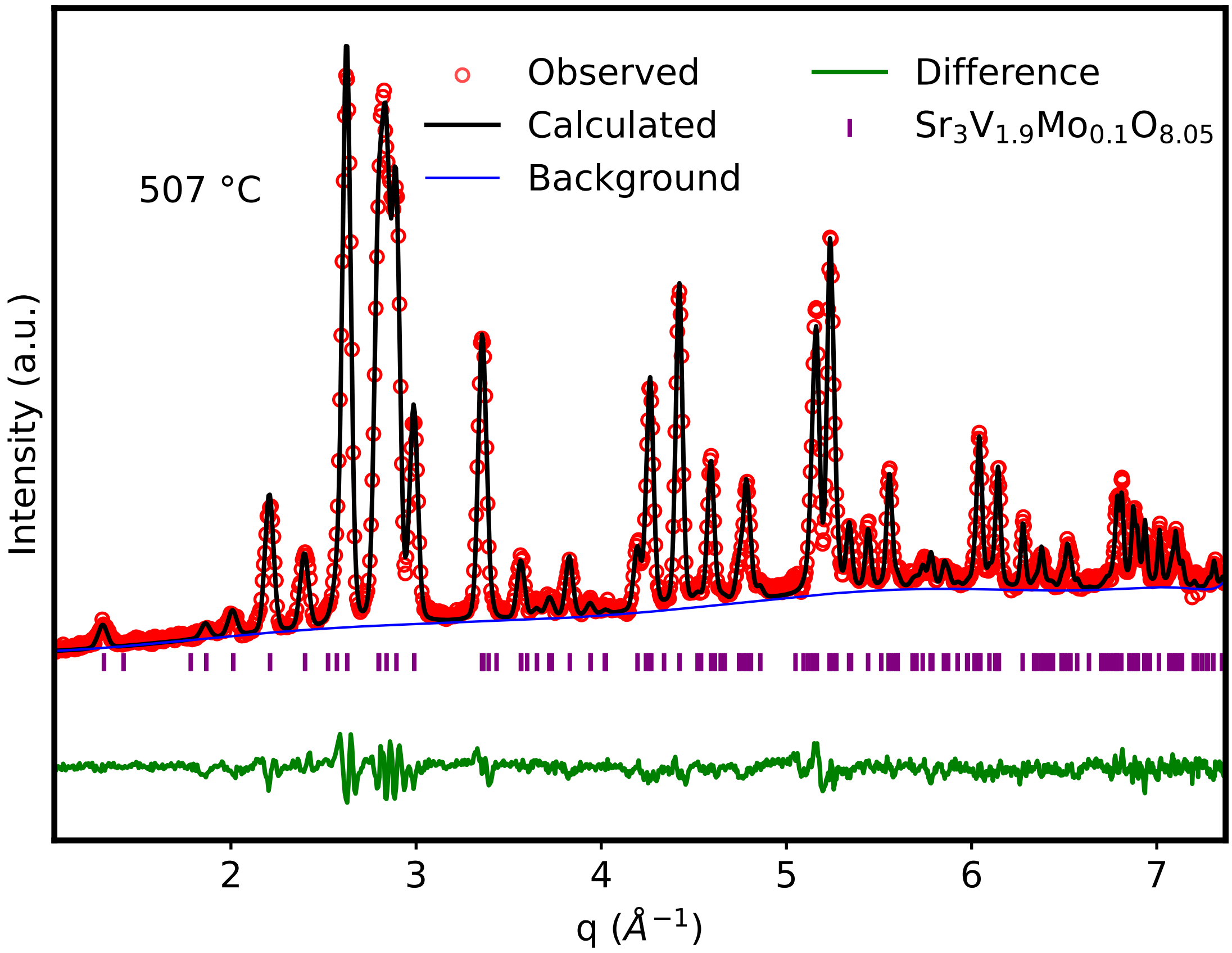

Figure S15. Rietveld refinement plot of the neutron pattern of $Sr_3V_{1.9}Mo_{0.1}O_{8.05}$ recorded at 507 °C.

Table S6. The crystallographic information derived from the refinement of the neutron pattern of $Sr_3V_{1.9}Mo_{0.1}O_{8.05}$ recorded at 507 °C.

Space group	R-3m					Neutron	
a,b (Å)	5.6828(4)				wR (%)	4.33	
c (Å)	20.1948(9)						
α, β (°)	90						
γ (°)	120						
Volume (Å^3)	564.80(4)						
label	Element	Multiplicity	x	y	z	Frac.	Uiso
Sr1	Sr	6	0	0	0.20303(2)	1	0.0226(5)
Sr2	Sr	3	0	0	0	1	0.0226(5)
Mo1	Mo	6	0	0	0.582(1)	0.05	0.0226(5)
V1	V	6	0	0	0.582(1)	0.95	0.0226(5)
O1	O	36	0.0607(1)	0.0508(2)	0.32252(2)	0.156 (2)	0.0035(2)
O2	O	36	0.5315(7)	0.5365(6)	0.23275(1)	0.515 (2)	0.0177(7)

Table S7. The crystallographic information of $Sr_3V_{1.8}Mo_{0.2}O_{8.1}$ derived from the combined refinement of X-ray and neutron diffraction powder patterns at room temperature with the inclusion of O2_s in the crystallographic model.

Space Group	R-3m				X-ray	Neutron	
a,b (Å)	5.64255(2)			wR(%)	9.7	6.63	
c (Å)	20.0799(5)						
αβ (°)	90						
γ (°)	120						
Volume ($Å^3$)	553.659(3)						
Label	Element	Multiplicity	x	y	z	Frac.	Uiso
Sr1	Sr	6	0	0	0.2018(2)	1	0.0116(6)
Sr2	Sr	3	0	0	0	1	0.015(1)
V1	V	6	0	0	0.4054(3)	0.9	0.003(2)
Mo1	Mo	6	0	0	0.4054(3)	0.1	0.003(2)
O1	O	36	0.044(3)	0.036(4)	0.3214(3)	0.156(2)	0.0084(6)
O2	O	36	0.5301(7)	0.5348(7)	0.2325(2)	0.500(3)	0.0083(7)
O2_s	O	36	0.70(1)	0.18(1)	0.249(3)	0.019(3)	0.0084(6)
	$Sr_3V_{1.8}Mo_{0.2}O_{8.1}$	V_2O_5	SrMoO4				
% composition	96.83	1.76	1.41				
% error	0.26	0.17	0.17				

Table S8. The crystallographic information of $Sr_3V_{1.8}Mo_{0.2}O_{8.1}$ was derived from the combined refinement of X-ray and neutron diffraction powder patterns at room temperature without the inclusion of O2_s in the crystallographic model.

Space Group	R-3m				X-ray	Neutron	
a,b (Å)	5.64255(2)			wR(%)	9.7	6.63	
c (Å)	20.0799(5)						
αβ (°)	90						
γ (°)	120						
Volume (Å^3)	553.659(3)						
Label	Element	Multiplicity	x	y	z	Frac.	Uiso
Sr1	Sr	6	0	0	0.2019(2)	1	0.0113(4)
Sr2	Sr	3	0	0	0	1	0.013(4)
V1	V	6	0	0	0.4049(3)	0.9	0.01 (fixed)
Mo1	Mo	6	0	0	0.4049(3)	0.1	0.01 (fixed)
O1	O	36	0.045(2)	0.03(1)	0.3216(2)	0.160(2)	0.0111(6)
O2	O	36	0.5339(7)	0.5339(7)	0.2323(1)	0.515(2)	0.0111(6)
	$Sr_3V_{1.8}Mo_{0.2}O_{8.1}$	V_2O_5	$SrMoO_4$				
% composition	96.89	1.69	1.42				
% error	0.26	0.25	0.09				

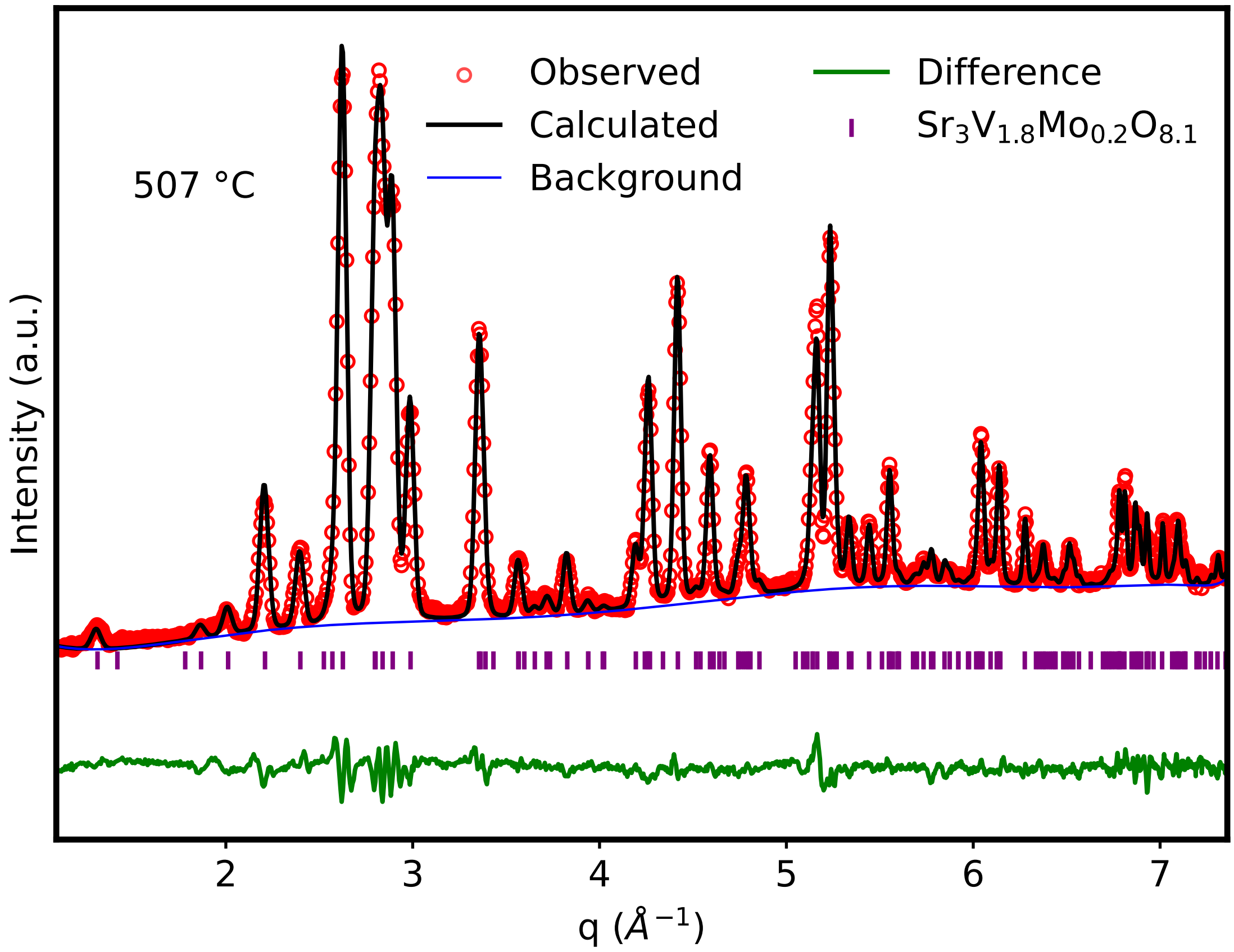

Figure S16. Rietveld refinement plot of the neutron pattern of $Sr_3V_{1.8}Mo_{0.2}O_{8.1}$ recorded at 507 °C.

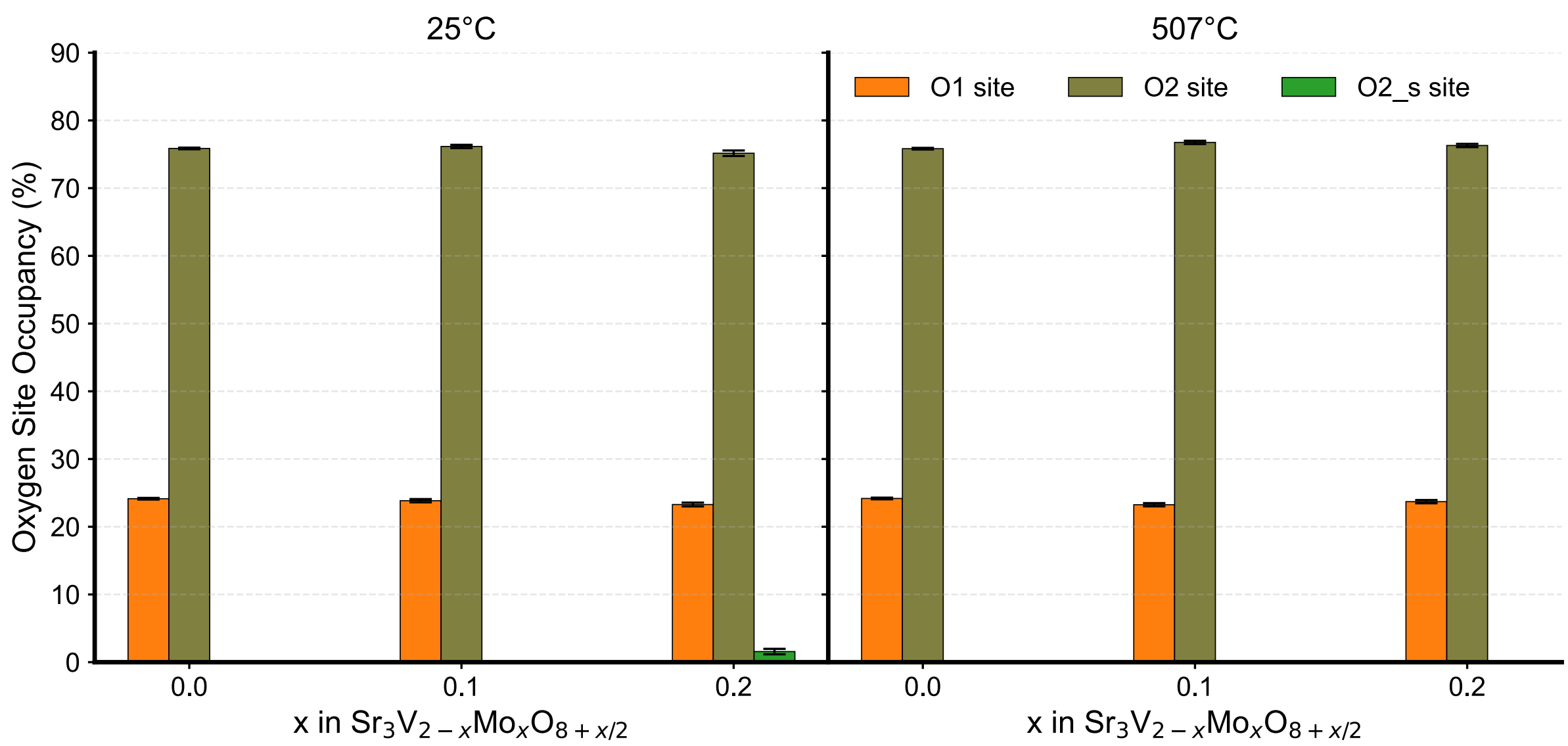

Figure S17. The evolution of the relative percentage occupancy of the oxide ion sites across the $Sr_3V_{2-x}Mo_xO_{8+x/2}$ series ($0 \leq x \leq 0.2$) with increasing x content and temperature.

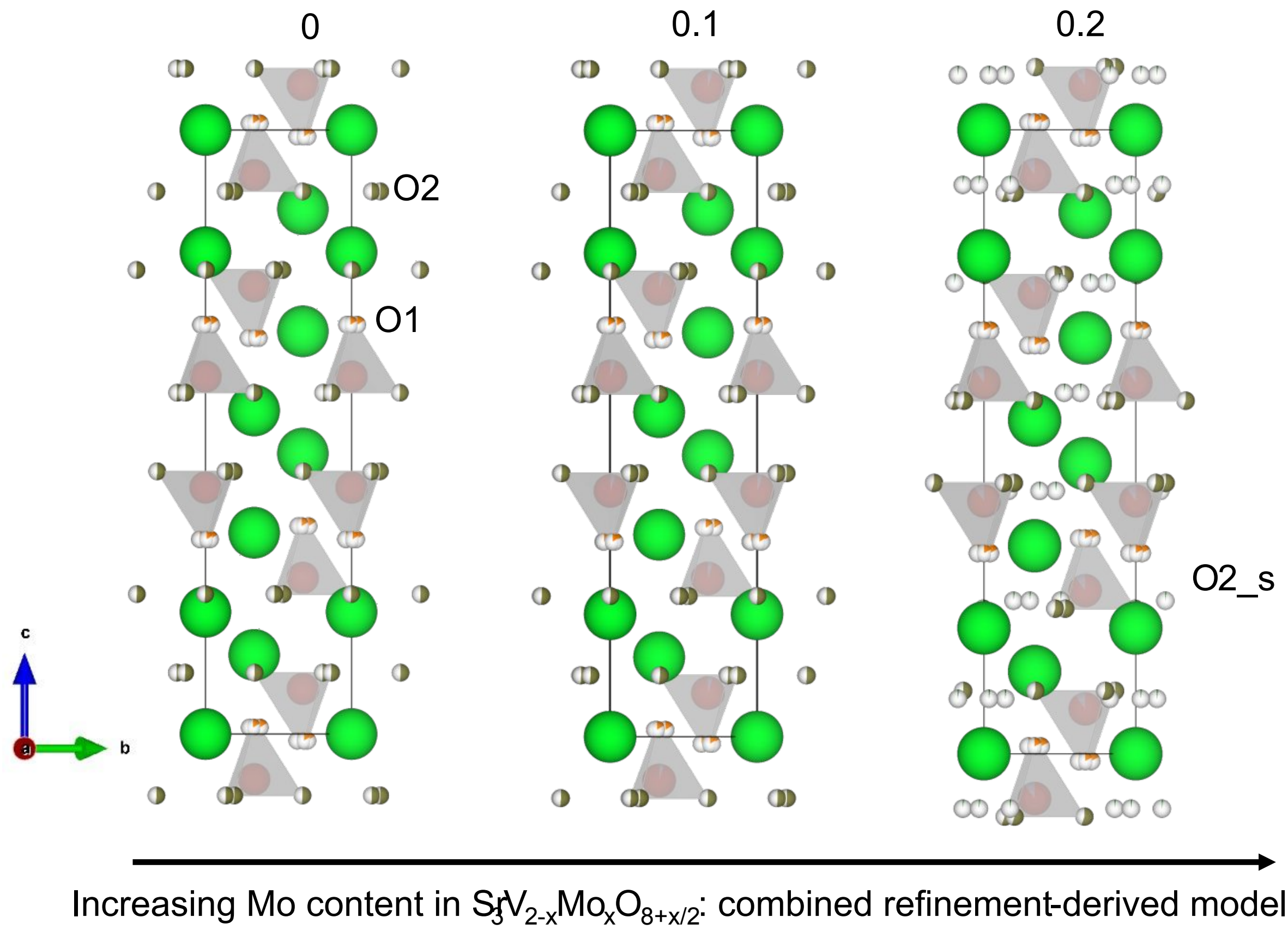

Figure S18. The refinement-derived crystallographic model of the $Sr_3V_{2-x}Mo_xO_{8+x/2}$ series ($0 \leq x \leq 0.2$).

Table S9. The crystallographic information derived from the neutron diffraction pattern of $Sr_3V_{1.8}Mo_{0.2}O_{8.1}$, recorded at 507 °C.

Space group	R-3m				Neutron		
a,b (Å)	5.6873(4)			wR (%)	4.27		
c (Å)	20.1875(8)						
α, β (°)	90						
γ (°)	120						
Volume (Å3)	565.49(4)						
label	Element	Multiplicity	x	y	z	Frac.	Uiso
Sr1	Sr	6	0	0	0.20284(2)	1	0.0233(6)
Sr2	Sr	3	0	0	0	1	0.0233(6)
V1	V	6	0	0	0.38	0.9	0.0233(6)
Mo1	Mo	6	0	0	0.38	0.1	0.0233(6)
O1	O	36	0.0613(1)	0.0491(2)	0.32266(2)	0.1589(2)	0.0059(2)
O2	O	36	0.5309(6)	0.5359(6)	0.23272(1)	0.536(5)	0.0205(7)

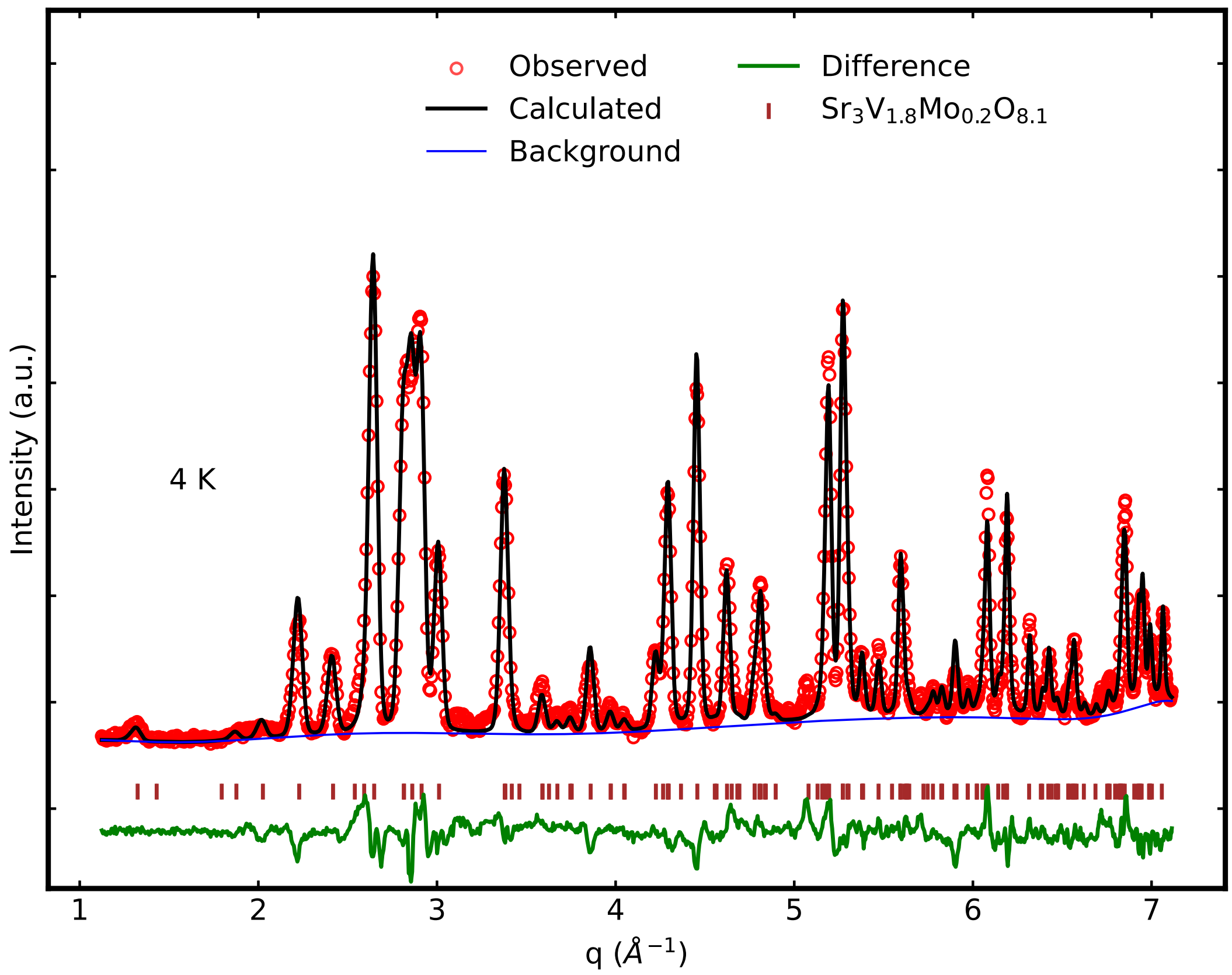

Figure S19. Refinement of $Sr_3V_{1.8}Mo_{0.2}O_{8.1}$ neutron diffraction pattern at 4 K.

Table S10. The crystallographic information derived from the neutron diffraction pattern of $Sr_3V_{1.8}Mo_{0.2}O_{8.1}$, recorded at 4 K.

Space group	R-3m				Neutron		
a,b (Å)	5.6384(5)			wR (%)	7.07		
c (Å)	20.084(1)						
α, β (°)	90						
γ (°)	120						
Volume (Å3)	552.96(5)						
label	Element	Multiplicity	x	y	z	Frac.	Uiso
Sr1	Sr	6	0	0	0.2024(2)	1	0.0074(6)
Sr2	Sr	3	0	0	0	1	0.0074(6)
V1	V	6	0	0	0.389(4)	0.9	0.0074(6)
Mo1	Mo	6	0	0	0.389(4)	0.1	0.0074(6)
O1	O	6	0	0	0.3214(2)	1	0.016(1)
O2	O	36	0.5364(8)	0.5387(7)	0.2318(2)	0.508(0)	0.0027(8)

^{51}V Solid-state Nuclear Magnetic Resonance

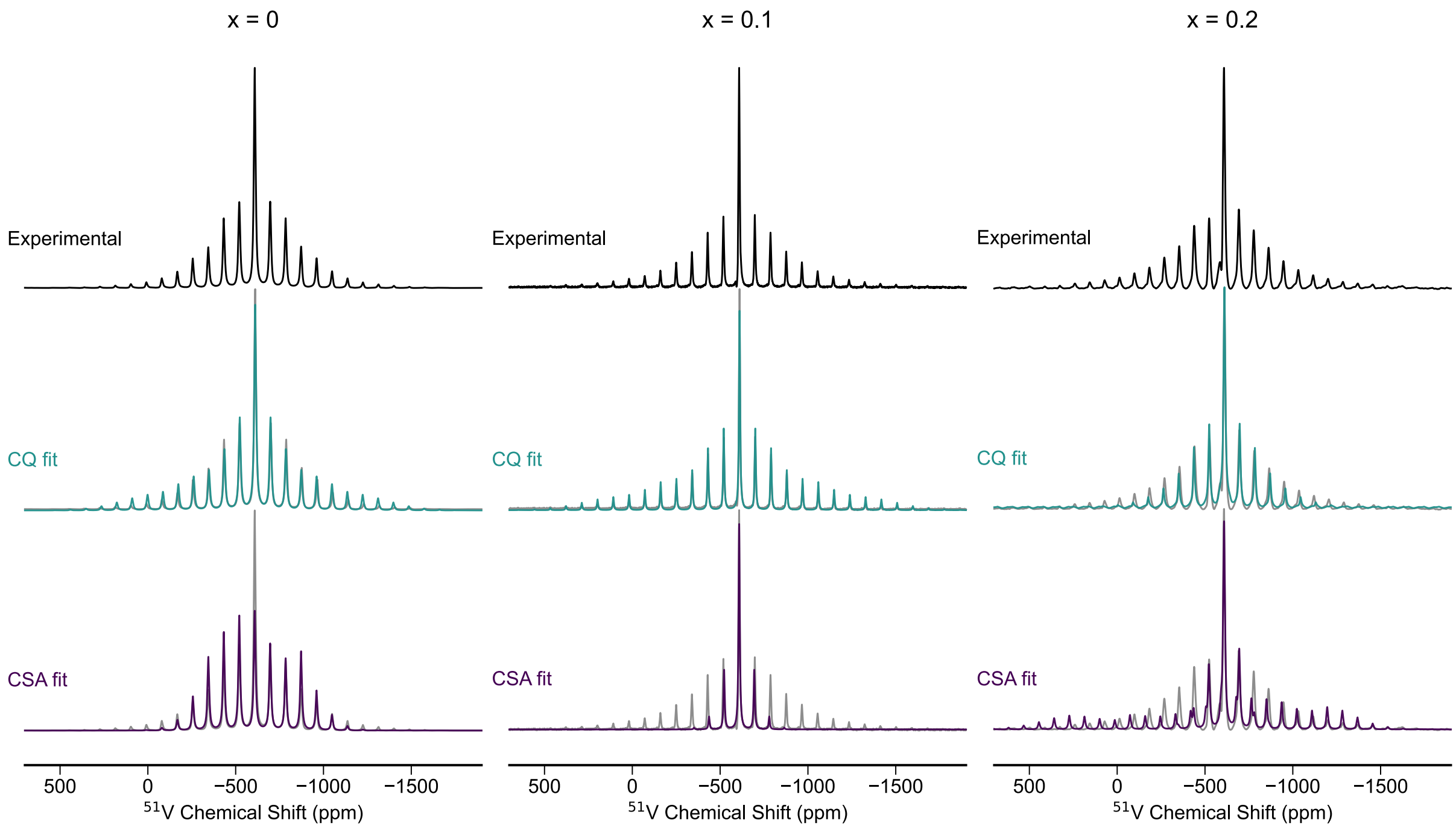

Figure S20. Simulation of the ^{51}V MAS NMR spectra of the $Sr_3V_{2-x}Mo_xO_{8+x/2}$ series with CSA and CQ models. The simulations were performed using the MRsimulator Python package.[6]

Table S11. A table of the optimized parameters obtained from the modeling of the [51]V MAS NMR spectra across the $Sr_3V_{2-x}Mo_xO_{8+x/2}$ series ($0 \leq x \leq 0.2$).

	Quadrupolar Coupling Model		
x	δ_{app} *(ppm)*	C_Q *(MHz)*	η_{CQ}
0	*-611*	*0.5717(6)*	*0.955(2)*
0.1	*-611*	*0.7499(7)*	*0.987(1)*
0.2	*-611*	*0.3833(8)*	*0.348(8)*

Table S12. Estimation of the percentage area of the two apparent chemical shifts observed in the center band of the [51]V MAS NMR spectra of the $Sr_3V_{2-x}Mo_xO_{8+x/2}$ series ($0.1 \leq x \leq 0.2$) in comparison with the percentage phase fractions and % total V obtained from the combined refinement of X-ray and neutron diffraction patterns.

x		*wt% (Rietveld)*	*% of total V*	*% area ([51]V)*
0.1	*palmierite*	*98.54 ± 0.24*	*95.9 ± 0.67*	*99.24 ± 1.35*
	V_2O_5	*1.46 ± 0.24*	*4.10 ± 0.67*	*0.76 ± 0.55*
0.2[a]	*palmierite*	*96.89 ± 0.26*	*94.91 ± 0.75*	*94.42 ± 2.58*
	V_2O_5	*1.69 ± 0.25*	*5.09 ± 0.75*	*5.58 ± 1.12*

[a] The refined weight fractions sum up to 98.58 wt% due to the presence of V-free $SrMoO_4$ phase

The [51]V MAS NMR spectra across the $Sr_3V_{2-x}Mo_xO_{8+x/2}$ series ($0 \leq x \leq 0.2$) could be well modeled with the first-order quadrupolar interaction (CQ), in contrast to the CSA parameters, where the modeling failed to reproduce the intensities of the center band and spinning sidebands, as well as the span of the sidebands (Figure S20). The fitted parameters are listed in Table S11. An empirical correlation between CSA parameters and the coordination number of vanadium in oxyvanadates has been established by Lapina *et al.*[7,8] Based on this correlation, we deduce that vanadium is in an isolated tetrahedral environment. This deduction is consistent with the reported average structure of the palmierite derivative, which comprises isolated VO_4 polyhedra stacked along the c-axis.[9,10] Moreover, this result is similar to that reported in a study that gauged the local coordination of vanadium in vanadium-substituted 7H hexagonal perovskite ($Ba_7Nb_{3.9-x}V_xMo_{1.1}O_{20.05}$).[11]

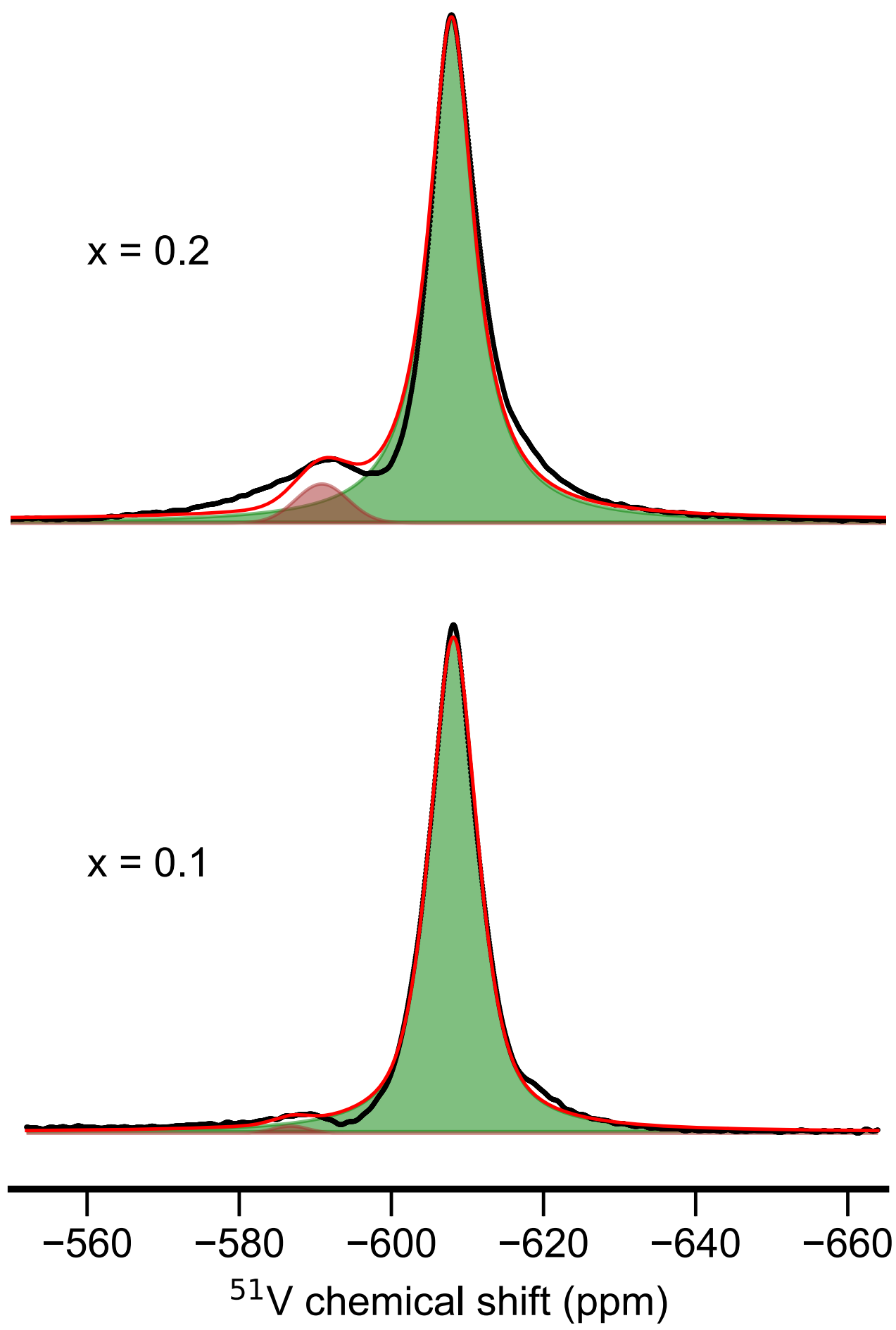

Figure S21. The ^{51}V MAS NMR spectra of $Sr_3V_{2-x}Mo_xO_{8+x/2}$ series (x = 0.1 and 0.2) zoomed in on the center band, illustrating two apparent chemical shifts at approximately -586 (brown) and 608 ppm (green), respectively. The peaks were deconvoluted using IonHop web-based NMR lineshape analysis software.[12]

^{17}O Solid-state Nuclear Magnetic Resonance

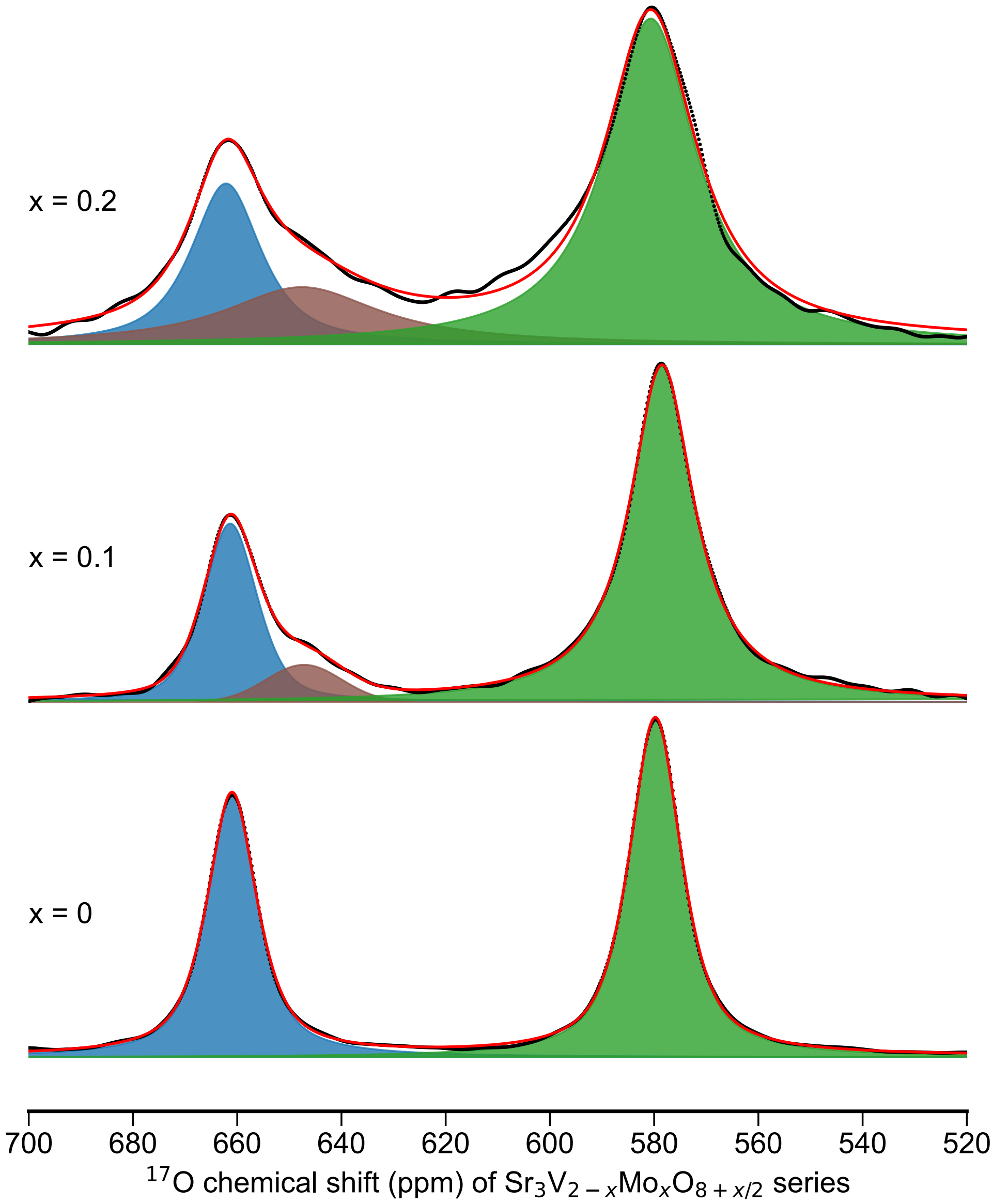

Figure S22. The evolution of ^{17}O MAS NMR spectra of the $Sr_3V_{2-x}Mo_xO_{8+x/2}$ series ($0 \leq x \leq 0.2$) with increasing x at 11.7 T. It is worth noting that the plot illustrates the center band of the respective NMR spectrum. The peaks were deconvoluted using IonHop web-based NMR lineshape analysis software.[12]

Table S13. ^{17}O NMR peak parameters for $Sr_3V_{2-x}Mo_xO_{8+x/2}$ series ($0 \le x \le 0.2$) at 11.7 T.

x	Peak position (ppm)	Width (Hz)	Lorentz-Gauss η	Area (%)
0	661	773 ± 2	0.73 ± 0.01	43 ± 1
	580	810 ± 2	0.73 ± 0.00	57 ± 1
0.1	661	831 ± 5	0.47 ± 0.02	25 ± 1
	647	1152 ± 35	0.00 ± 0.08	6 ± 1
	580	1014 ± 3	1.00 ± 0.01	69 ± 1
0.2	662	1088 ± 41	0.79 ± 0.06	21 ± 2
	648	2451 ± 198	1.00 ± 0.20	18 ± 5
	581	1435 ± 9	0.98 ± 0.02	61 ± 4

Table S14. ^{17}O NMR peak parameters for $Sr_3V_{2-x}Mo_xO_{8+x/2}$ series ($0 \le x \le 0.2$) at 20 T.

x	Peak position (ppm)	Width (Hz)	Lorentz-Gauss η	Area (%)
0	660	610 ± 5	0.54 ± 0.02	43 ± 2
	581	623 ± 4	0.58 ± 0.02	57 ± 2
0.1	660	1692 ± 50	0.40 ± 0.07	23 ± 3
	646	2350 ± 290	1.00 ± 0.28	10 ± 4
	580	2047 ± 18	0.93 ± 0.02	67 ± 4
0.2	660	2033 ± 46	0.44 ± 0.07	20 ± 2
	642	3296 ± 214	1.00 ± 0.20	16 ± 4
	579	2518 ± 19	1.00 ± 0.03	61 ± 4
	553	2054 ± 344	1.00 ± 0.44	3 ± 1

Table S15. Field dependence analysis of the Gauss-Lorentz-fitted lineshapes in the $Sr_3V_{2-x}Mo_xO_{8+x/2}$

Composition	***δ (ppm)***	***Δv, 11.7 T (Hz)***	***Δv, 20 T (Hz)***	***Ratio***
x = 0	*661*	*773*	*610*	*0.79*
x = 0	*580*	*810*	*623*	*0.77*
x = 0.1	*660*	*831*	*1692*	*2.04*
x = 0.1	*647*	*1152*	*2350*	*2.04*
x = 0.1	*580*	*1014*	*2047*	*2.02*
x = 0.2	*662*	*1088*	*2033*	*1.87*
x = 0.2	*580*	*1435*	*2518*	*1.75*

Bond Valence Site Energy Calculations

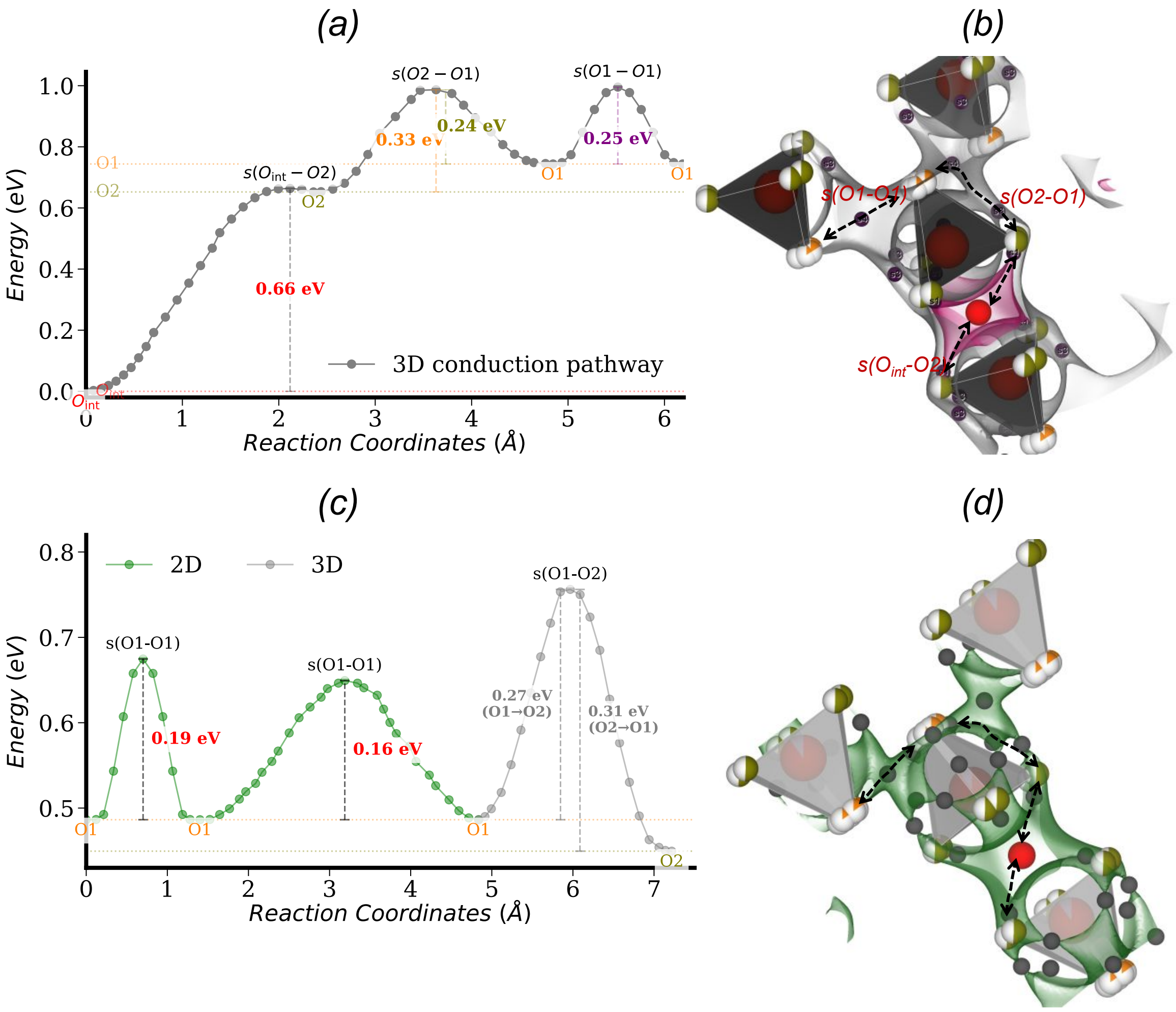

Figure S23. $Sr_3V_2O_8$ (a) Bond-valence site energy landscape and (b) conduction map. $Sr_3V_{1.8}Mo_{0.2}O_{8.1}$ (c) BVSE migration energy landscape in the unconstrained model and (d) the conduction map.

For the $Sr_3V_2O_8$ conduction pathways, it is worth noting that the calculations introduce an interstitial O^{2-} site (O_{int}) at 3b Wyckoff with coordinates (0, 0, 1/2), which is not present in the refinement-derived model. The computed O^{2-} migration pathway involves three distinct saddle points associated with inter- and intra-polyhedral oxygen jumps, namely s(O_{int}-O2), s(O2-O2), and s(O1-O1). The inter-polyhedral migration between O2 sites of vertically adjacent VO_4 polyhedra proceeds via the O_{int} site (Figure S23a,b), traversing the s(O_{int}-O2) saddle point with the highest barrier of 0.66 eV, indicating that this step likely constitutes the rate-limiting process for O^{2-} transport between VO_4 moieties stacked along the c-axis. In contrast, migration between apical O1 sites of neighboring VO_4 units occurs via the s(O1-O1) saddle point with a comparatively low barrier of 0.25 eV, suggesting that long-range O^{2-} migration may preferentially propagate within the ab-plane through successive O1-O1 hops. Intra-polyhedral O^{2-} jumps within a single VO_4 unit proceed through the s(O1-O1) saddle point, where an O2 ion migrates toward the apical O1 position via a two-step process with barriers of 0.33 eV (O2→s(O2-O1)) and 0.24 eV (s(O2-O1)→O1). Together, these migration mechanisms are consistent with the proposed “cog-wheel” mechanism, in which oxygen transport is facilitated by cooperative rotation of VO_4 and oxygen-deficient VO_3 units, accompanied by transient V_2O_7 dimer formation through continuous bond breaking and reformation.[9,13]

For the uncontrained $Sr_3V_{1.8}Mo_{0.2}O_{8.1}$ calculation, the 2D O1-O1 pathway exhibits saddles with 0.19 and 0.162 eV, where the connectivity of O1 to O2 goes via asymmetric barriers of 0.27 and 0.31 eV (Figure S23c,d). The s(O1-O2) barriers are relatively comparable to their equivalence in the BVSE landscape of the pristine $Sr_3V_2O_8$ (Figure S23a). The O3-mediated 2D pathway observed in the AIMD trajectories at 600 K is not accessed without the rigid-lattice constraint on O2; constraining O2 in line with the AIMD finding is therefore required for the BVSE map to be consistent with the AIMD observation.

BVSE migration barriers are computed for a single elementary hop on a static lattice and are well established to differ from DFT-NEB and experimental values; the absolute numbers should therefore be treated qualitatively, while the relative ranking of pathways is robust. Two further observations from the BVSE calculations are nevertheless directly informative for the experimental comparison of Ea. First, the intra-polyhedral O2-O1 barrier is essentially unchanged between pristine $Sr_3V_2O_8$ and $Sr_3V_{1.8}Mo_{0.2}O_{8.1}$ (~0.3 eV in both compositions) (Figure S23), confirming that this framework-intrinsic hop is not modulated by Mo substitution. Second, the 0.66 eV s(O_{int}–O2) cog-wheel step that dominates the rate-limiting pathway in pristine $Sr_3V_2O_8$ is no longer in the rate-limiting route for the constrained $Sr_3V_{1.8}Mo_{0.2}O_{8.1}$, because the new 2D O1-O3 pathway (~0.16 eV) bypasses it. These two findings support the similarity in Arrhenius slopes across the series: the macroscopic Ea is set by whichever framework-intrinsic hop is the bottleneck for percolation across the polycrystalline pellet, and that hop is the same across the series. And the incorporation of Mo does not alter it; instead, it adds carriers via charge compensation and introduces a parallel, low-barrier in-plane connectivity between the O1 and O3 sites, both of which increase conductivity.

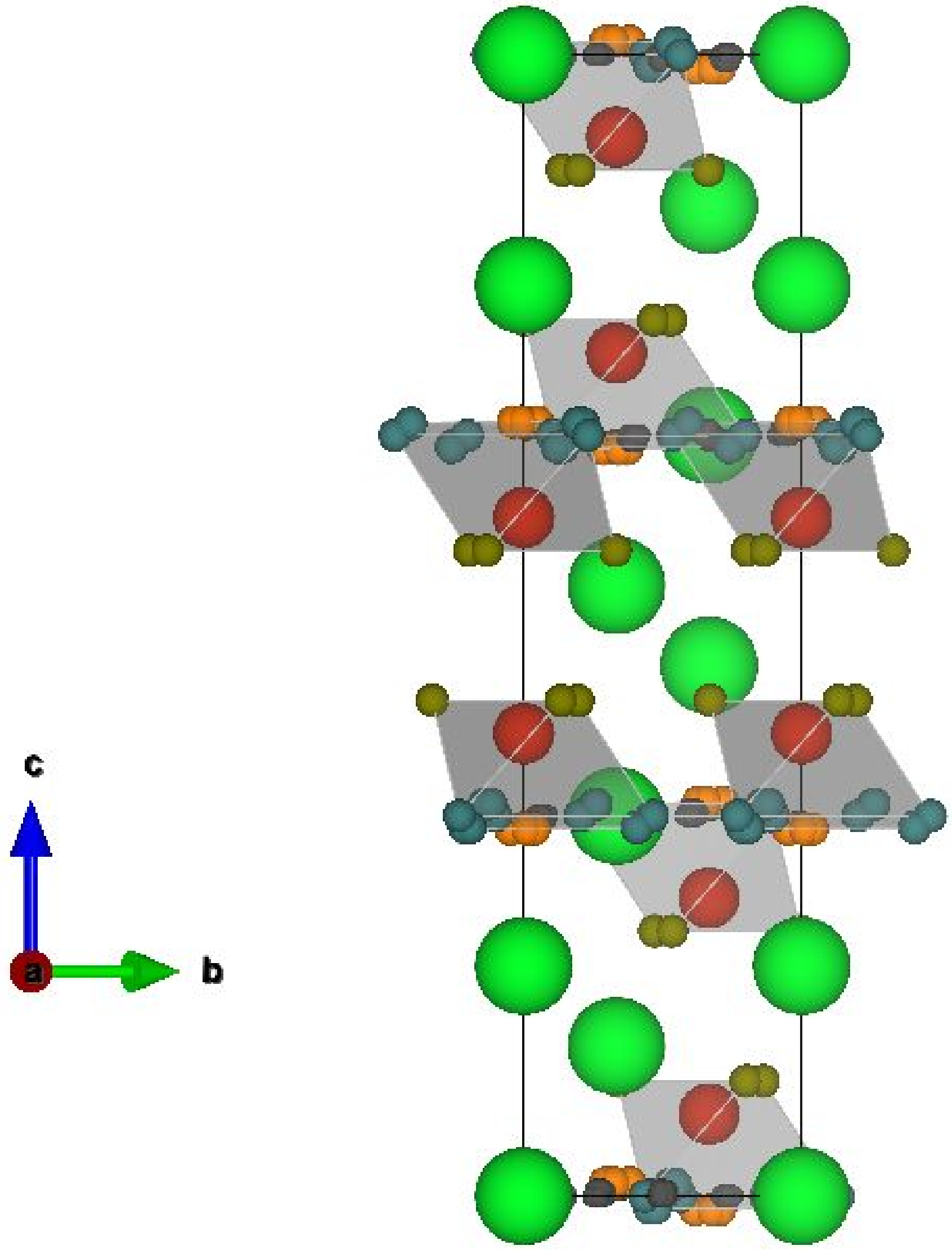

Figure S24. BVSE-derived crystallographic structure illustrating how the interstitial sites transformed the isolated VO_4 units into isolated corner-sharing polyhedra with mixed coordination geometries separated by octahedral M2 vacancies.

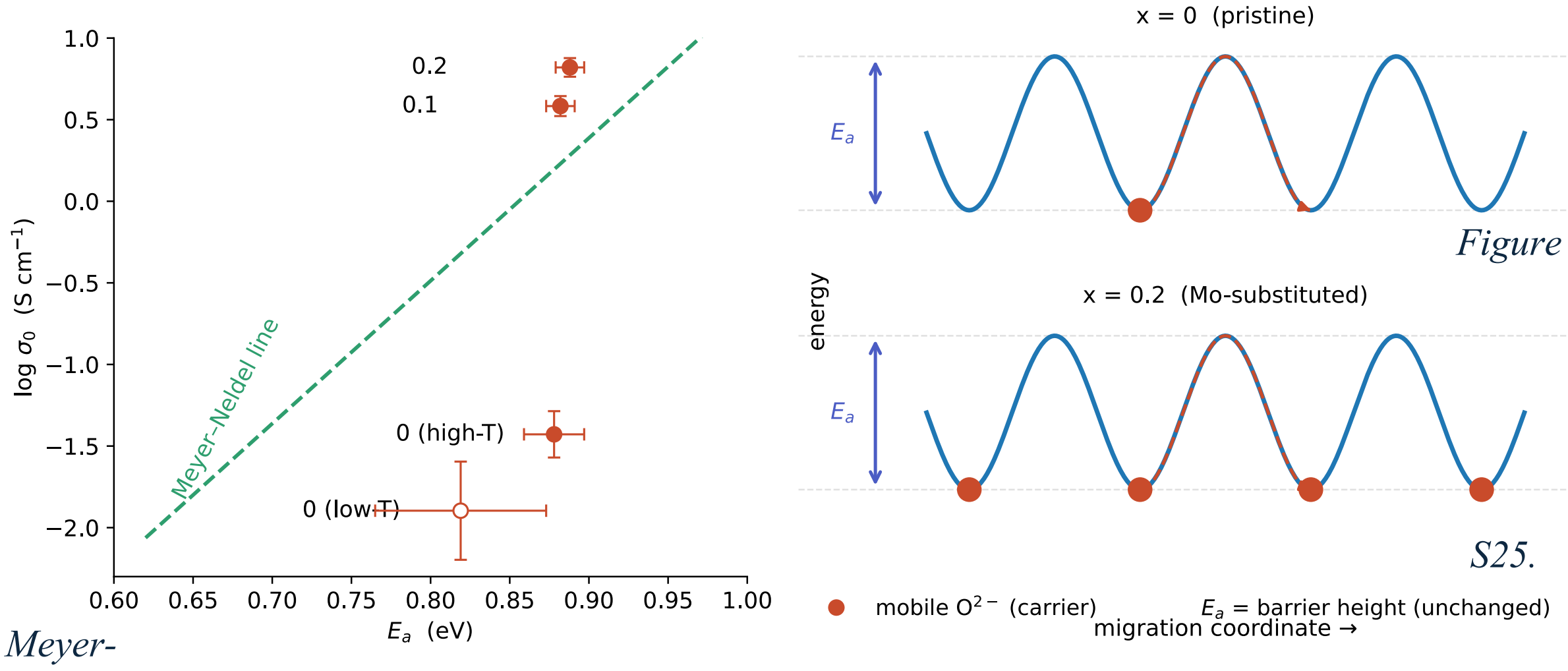

Figure S25. Meyer-Nédel (M-N) compensation plot contrasting a schematic M-N relationship (green line) with experimental data (brown data points). An energy landscape diagram illustrating where the migration barrier height remains constant across different compositions, while the pre-exponential factor varies with carrier density and available pathways rather than with barrier geometry. The carrier density is slightly overestimated, as incorporating x amount of Molybdenum into the palmarite structure introduces x/2 charge-compensating oxygen per formula unit ($Sr_3V_{2-x}Mo_xO_{8+x/2}$).

References

1. Bastow, T. J., Dirken, P. J., Smith, M. E. & Whitfield, H. J. Factors Controlling the 17O NMR Chemical Shift in Ionic Mixed Metal Oxides. *J. Phys. Chem.* **100**, 18539–18545 (1996).
2. Oldfield, E. *et al.* 17O nuclear-magnetic-resonance spectroscopic study of high-*Tc* superconductors. *Phys. Rev. B* **40**, 6832–6849 (1989).
3. Kim, N. & Stebbins, J. F. Sc2(WO4)3 and Sc2(MoO4)3 and Their Solid Solutions: 45Sc, 17O, and 27Al MAS NMR Results at Ambient and High Temperature. *Chem. Mater.* **21**, 309–315 (2009).
4. Schönleber, M., Klotz, D. & Ivers-Tiffée, E. A Method for Improving the Robustness of linear Kramers-Kronig Validity Tests. *Electrochimica Acta* **131**, 20–27 (2014).
5. Murbach, M., Gerwe, B., Dawson-Elli, N. & Tsui, L. impedance.py: A Python package for electrochemical impedance analysis. *J. Open Source Softw.* **5**, 2349 (2020).
6. Srivastava, D. J., Giammar, M., Venetos, M. C., McCarthy-Carney, L. & Grandinetti, P. J. MRSimulator: A cross-platform, object-oriented software package for rapid solid-state NMR spectral simulation and analysis. *J. Chem. Phys.* **161**, 212501 (2024).
7. Lapina, O. B., Mastikhin, V. M., Shubin, A. A., Krasilnikov, V. N. & Zamaraev, K. I. 51V Solid state NMR studies of vanadia based catalysts. *Prog. Nucl. Magn. Reson. Spectrosc.* **24**, 457–525 (1992).
8. Lapina, O. B., Khabibulin, D. F., Shubin, A. A. & Terskikh, V. V. Practical aspects of 51V and 93Nb solid-state NMR spectroscopy and applications to oxide materials. *Prog. Nucl. Magn. Reson. Spectrosc.* **53**, 128–191 (2008).
9. Fop, S. *et al.* Proton and Oxide Ion Conductivity in Palmierite Oxides. *Chem. Mater.* **34**, 8190–8197 (2022).

10. Skakle, J. M. S., Coats, A. M. & Marr, J. The crystal structures of Ba2R2/3V2O8 (R = La, Nd) and Sr2La2/3V2O8; palmierite derivatives. *J. Mater. Sci.* **35**, 3251–3256 (2000).

11. Olatunbosun Biffo, A., Famprikis, T. & B. Groszewicz, P. The role of vanadium substitution in the oxygen sublattice disorder of Ba 7 Nb 4 MoO 20 -based hexagonal perovskite oxide-ion conductors. *J. Mater. Chem. A* **12**, 30082–30095 (2024).

12. IonHop: NMR lineshape and relaxation analysis. *IonHop* https://www.ionhop.com/.

13. Watson, V. *et al.* Enhanced Oxide Ion Diffusion by Lanthanum Substitution in the Palmierite Sr3–3xLa2xV2O8 via Increased Tetrahedral Distortion and Cation Vacancies. *Chem. Mater.* https://doi.org/10.1021/acs.chemmater.5c01856 (2025) doi:10.1021/acs.chemmater.5c01856.
